# An Alkyne‐Metathesis‐Based Approach to the Synthesis of the Anti‐Malarial Macrodiolide Samroiyotmycin A

**DOI:** 10.1002/anie.202105732

**Published:** 2021-07-20

**Authors:** Ektoras Yiannakas, Mark I. Grimes, James T. Whitelegge, Alois Fürstner, Alison N. Hulme

**Affiliations:** ^1^ School of Chemistry University of Edinburgh Joseph Black Building, David Brewster Road Edinburgh EH9 3FJ UK; ^2^ Department of Organometallic Chemistry Max-Planck-Institut für Kohlenforschung 45470 Mülheim/Ruhr Germany

**Keywords:** alkynes, hydrostannation, metathesis, natural products, total synthesis

## Abstract

We report the first total synthesis of samroiyotmycin A (**1**), a C_2_‐symmetric 20‐membered anti‐malarial macrodiolide isolated from Streptomyces sp. The convergent synthetic strategy orchestrates bisalkyne fragment‐assembly using an unprecedented Schöllkopf‐type condensation on a substituted β‐lactone and an ambitious late‐stage one‐pot alkyne cross metathesis–ring‐closing metathesis (ACM–RCAM) reaction. The demanding alkyne metathesis sequence is achieved using the latest generation of molybdenum alkylidynes endowed with a tripodal silanolate ligand framework. Subsequent conversion to the required E‐alkenes uses contemporary hydrometallation chemistry catalysed by tetrameric cluster [{Cp*RuCl}_4_].

## Graphical abstract

Forging of the 20‐membered macrocylic core of samroiyotmycin A can be readily achieved by combining a Mo‐catalysed one‐pot alkyne cross‐metathesis ring‐closing metathesis of bisalkyne building blocks and Ru‐catalysed hydrometallation chemistry. These key transformations have enabled the first total synthesis of this anti‐malarial *C*_2_‐symmetric macrodiolide.

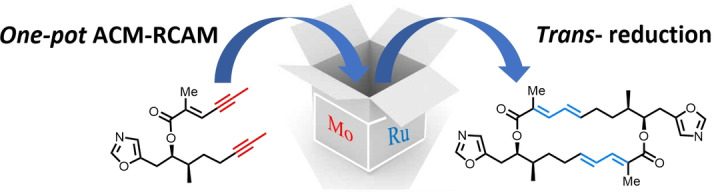

In 2013, Pittayakhajonwut and co‐workers reported that the crude extracts of *Streptomyces* sp. BCC33756 exhibited potent activity against a multi‐drug resistant (MDR) malarial strain.^[1]^ A *C_2_*‐symmetric macrodiolide from this extract, samroiyotmycin A (**1**), was shown to be active against both the MDR malarial strain *P. falciparum* K1 (IC_50_: 3.65 μg mL^−1^) and lung carcinoma cell line NCI‐H187 (IC_50_: 24.14 μg mL^−1^).^[1]^ We envisaged rapid access to this promising scaffold using a one‐pot alkyne cross metathesis–ring‐closing alkyne metathesis (ACM–RCAM) approach. Our previous work on the macrodiolide disorazole C_1_ ^[2]^ suggested this would allow rapid construction of the macrodiolide core with control over both the orientation of subunits and ring size.

A priori, the initial disconnection for this ACM–RCAM approach can be implemented across either the *E*‐configured C(5)−C(6)/C(21)−C(22) di‐substituted alkenes, or the C(3)−C(4)/C(19)−C(20) *E*‐enoates (black and grey routes, respectively, Scheme 1 (i)). Comparing end‐games: in the former strategy, conversion of bis(enyne) **2** to the *E*‐configured alkenes in the absence of protic groups proximal to the acetylene is likely to be challenging;[^3^, ^4^, ^5^] whilst in the second strategy, conversion of bis(ynoate) precursor **3** to samroiyotmycin A via ynoate–dienoate isomerisation,[^6^, ^7^, ^8^, ^9^, ^10^, ^11^, ^12^, ^13^] bisiodination,^[14]^ and double Stille/Negishi coupling[^15^, ^16^] has better precedent. Examining the ACM–RCAM step, although both ACM and RCAM reactions of ynoates have been explored in isolation previously, the number of successful examples is small,[^17^, ^18^, ^19^, ^20^, ^21^, ^22^, ^23^] likely because electron‐deficient triple bonds in general are poorly reactive and ynoates in particular were beyond reach of the classical catalysts.[^24^, ^25^] To our knowledge there are no examples of a one‐pot ynoate ACM–RCAM, as required for the assembly of the 20‐membered core of samroiyotmycin A (**1**). With this in mind, we devised a unified strategy for construction of the bisalkyne building blocks, which would allow us to explore both routes. This strategy centred on desulfonylation of 5‐substituted 4‐tosyloxazoles **7** and esterification of the resultant secondary alcohols **6** to give bisalkyne fragments **4** and **5**. Influenced by prior work,^[26]^ we hypothesised that formation of linchpin fragments **7** (Scheme 1 (ii)) could be achieved through a Schöllkopf‐type condensation of 4‐substituted β‐lactones **8** with anionic TosMIC.[^27^, ^28^, ^29^] If successful, this ambitious strategy would allow simultaneous installation of the 1,3‐oxazole unit and stereochemistry at C(10). Furthermore, we envisaged that construction of the diastereomerically pure β‐lactones **8** would be possible via an asymmetric ketene cycloaddition to α‐methyl aldehydes **9**.^[30]^ Our synthetic approach to exploring these two proposed one‐pot ACM–RCAM strategies is outlined below.

**Scheme 1:**
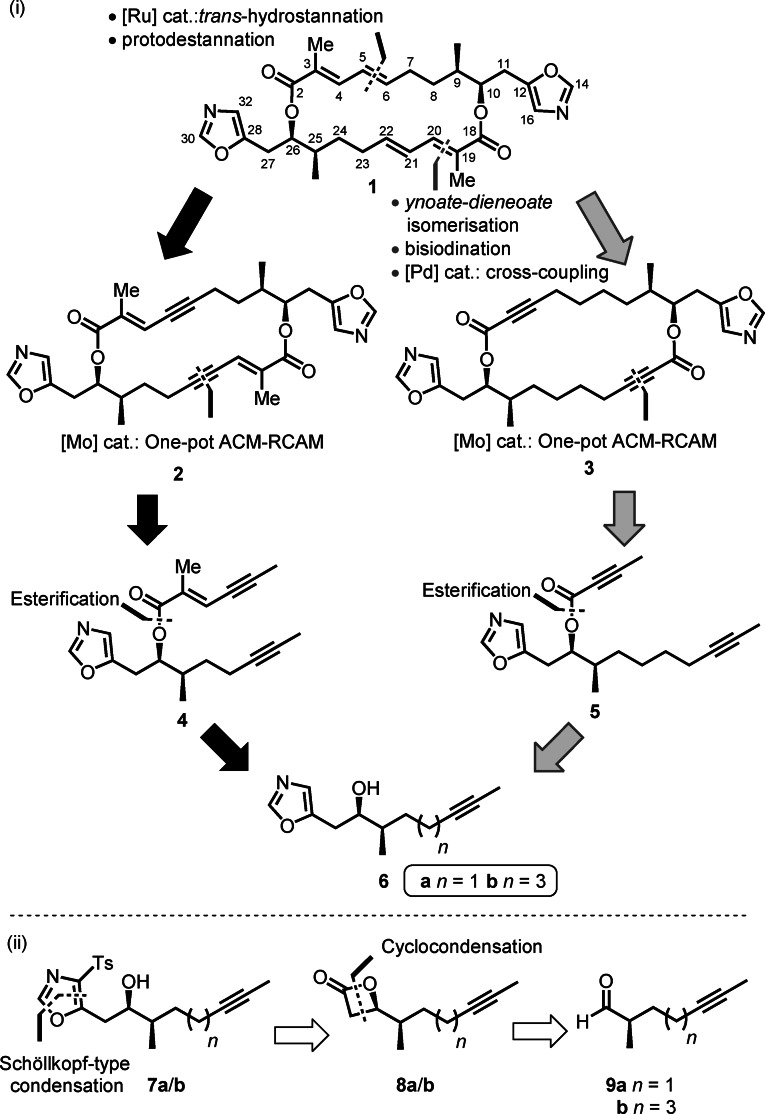
Convergent one‐pot ACM–RCAM approaches to samroiyotmycin A. (i) Ring deconstruction via routes based on C(5)−C(6)/C(21)−C(22) or C(3)−C(4)/C(19)−C(20) disconnections to key intermediate **6**; (ii) Installation of C(9)/C(10) and C25)/C(26) stereochemistry via cyclocondensation onto α‐chiral aldehyde **9**.

To explore our novel synthetic approach to setting the C(9)−C(10) stereochemistry required generation of α‐chiral aldehydes **9 a**/**b**. Our initial approach focused on the diastereoselective alkylation of *N*‐acylated oxazolidinones (Scheme 2 (i)). Due to competing elimination reactions observed when alkylating the propionate derivative, diastereoselective methylation of the oxazolidinone derivatives **10 a**/**b** (see SI for preparation) was the preferred route. This reaction delivered the *N*‐acyl derivatives **11 a**/**b** on a multi‐gram scale in good to excellent yield (77–98 %) and with high selectivity (>20:1 dr).^[31]^ Hydrolytic cleavage of the chiral auxiliary in the presence of H_2_O_2_ gave the corresponding acids **12 a**/**b** in almost quantitative yield (95 %). Conversion to the configurationally stable Weinreb amides **13 a**/**b** was accompanied by a very slight erosion of enantiomeric purity, as observed by HPLC on a chiral stationary phase. DIBAL‐H reduction (Scheme 3 (i)) provided the sensitive α‐methyl aldehydes **9 a**/**b**, primed for chain elongation by an acetate aldol equivalent.^[32]^ The asymmetric ketene–aldehyde cycloaddition catalysed by the Lewis base *O*‐TMS quinine **14** was chosen for this purpose due to high levels of stereochemical control exerted by this cinchona alkaloid catalyst in intramolecular nucleophile‐catalysed aldol lactonisation (NCAL) reactions of α‐ or β‐substituted aldehyde acids,^[33]^ and in intermolecular NCAL reactions featuring aldehydes with remote substitution.^[34]^ This reaction converted crude aldehyde **9 a** into the thermally unstable β‐lactone **8 a** with excellent diastereocontrol over the newly formed stereocentre at C(10) (>99:1 dr). To the best of our knowledge, this is the first example of an intermolecular NCAL reaction with double diastereodifferentiation, where the catalyst control dominates over the influence of an α‐chiral centre on the aldehyde. Immediate low‐temperature Schöllkopf‐type condensation of **8 a** with anionic TosMIC furnished chiral alcohol **7 a** in excellent overall yield (35 % from oxazolidinone **11 a**, following a five‐step telescoped sequence) and with high diastereoselectivity (96:4 dr). When repeated with the longer‐chain oxazolidinone **11 b**, the same five‐step sequence yielded alcohol **7 b** in 43 % overall yield (>99:1 dr). In both cases, the configuration at C(10)OH was confirmed by conversion to Mosher esters **15** and **16** (see SI for details).

**Scheme 2:**
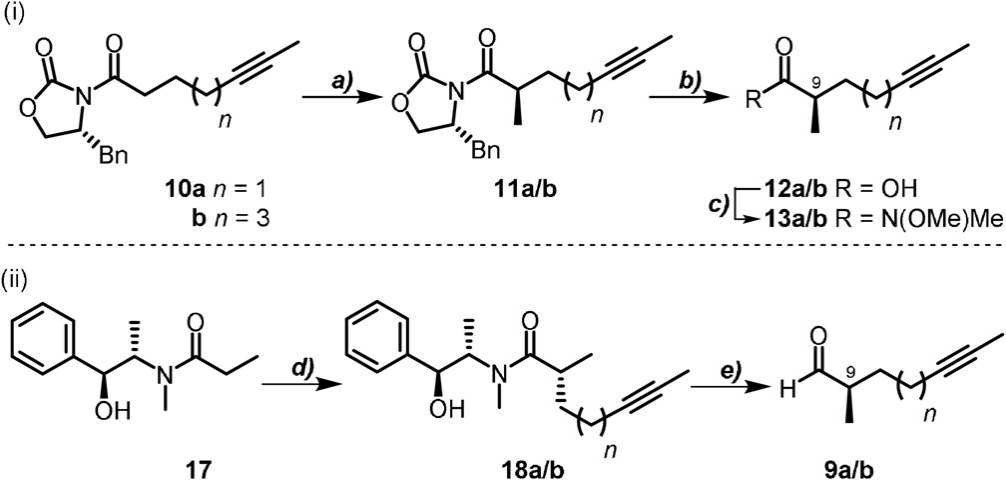
Setting C(9) stereochemistry via alkylation of an acylated Evans’ oxazolidinone (i), or Myers’ pseudoephedrine (ii) auxiliary. Reagents and Conditions: a) MeI, NaHMDS, THF, −78 °C, 4.5 h, (**11 a**, 77 %, >20:1 dr; **11 b**, 98 %, >20:1 dr); b) LiOH, H_2_O_2_, THF/H_2_O (1:1), 0 °C→rt, (**12 a**, 95 %, 93 % *ee*; **12 b**, 94 % *ee*); c) CDI, HN(OMe)Me⋅HCl, CH_2_Cl_2_, 0 °C→rt, 18 h, (**13 a**, 93 % *ee*; **13 b**, 82 % from **11 b**, 92 % *ee*); d) LDA, LiCl, 5‐iodopent‐2‐yne *or* 7‐iodohept‐2yne, −78→0 °C, 2 h, (**18 a**, 79 %, 75:1 dr; **18 b**, 98 %, 71:1 dr); e) LiAlH_4_, EtOAc, THF/isohexanes (4:3), −78 °C→0 °C, 1 h.

**Scheme 3:**
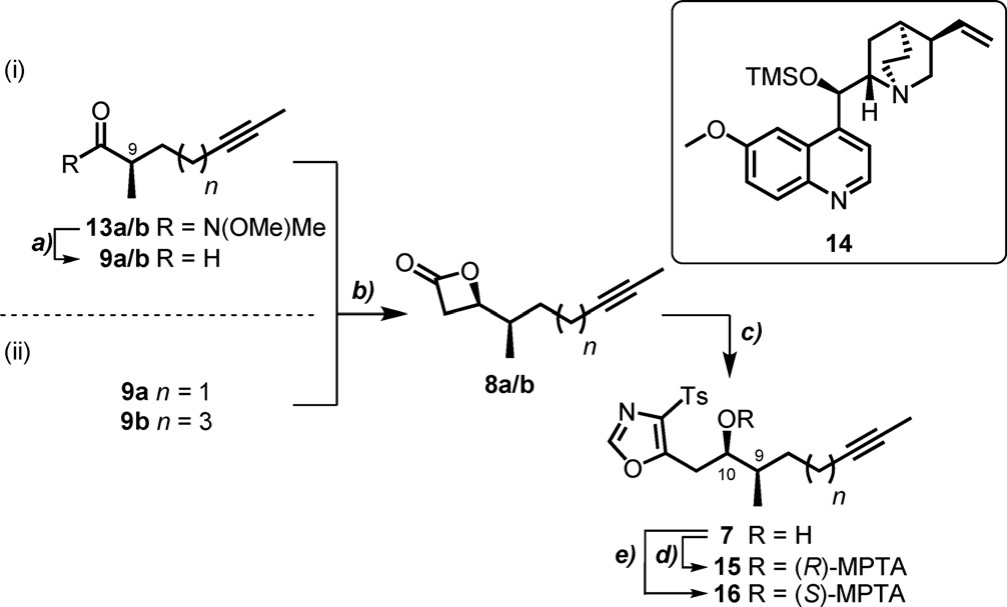
Introduction of C(10)OH and 4‐tosyloxazole functionalities. Reagents and Conditions: a) DIBAL‐H, CH_2_Cl_2_, −78 °C, 1 h; b) **14** (10 mol %), LiClO_4_, AcCl, DIPEA, CH_2_Cl_2_/Et_2_O (1:1–2:1), −78 °C, 18–24 h, >99:1 dr; c) TosMIC, ^*n*^BuLi, THF, −78 °C→rt, 1 h (**7 a**, 35 %, 96:4 from **11 a**, or 52 %, >97:3 dr from **18 a**; **7 b** 43 %, >20:1 dr from **11 b**, or 24 %, >20:1 dr from **18 b**); d) (*S*)‐(+)‐MTPACl, DMAP (10 mol %), TEA, CH_2_Cl_2_, rt, 18 h, (**15 a**, 77 %; **15 b**, 85 %), e) (*R*)‐(−)‐MTPACl, DMAP (10 mol %), TEA, CH_2_Cl_2_, rt, 18 h (**16 a**, 72 %; **16 b**, 88 %).

For the total synthesis of samroiyotmycin A, we streamlined our route by switching to the use of Myers’ acylated pseudoephedrine auxiliary **17**, since the amide formed upon alkylation can be directly converted to the enantioenriched α‐methyl aldehyde (Scheme 2 (ii)). Diastereoselective alkylation of the lithium enolate of **17** using 5‐iodopent‐2‐yne afforded multigram quantities of amide **18 a** in very good yield (79 %, 75:1 dr). Reductive cleavage of the auxiliary using LiAl(OEt)_3_H generated in situ at low temperature gave aldehyde **9 a**,[^35^, ^36^] which was used directly in the cyclocondensation catalysed by *O*‐TMS quinine **14** to afford β‐lactone **8 a** (>99:1 dr). Immediate condensation of crude **8 a** with anionic TosMIC provided chiral alcohol **7 a** in excellent yield (52 %, over a three‐step telescoped sequence) and with high diastereoselectivity (97:3 dr) on multi‐gram scale. 4‐Tosyloxazole **7 b** was also obtained by adaptation of this synthetic route; asymmetric alkylation of the acylated auxiliary with 7‐iodohept‐2‐yne to give **18 b**, reduction to aldehyde **9 b**, and elaboration to diastereomerically pure β‐lactone **8 b**. Careful optimisation of the cycloaddition reaction conditions showed that use of an equivolume mixture of Et_2_O and CH_2_Cl_2_ as solvent gave the highest conversion and diastereoselectivity for this substrate. Subsequent condensation of the crude lactone **8 b** with TosMIC delivered fragment **7 b** in good yield (24 % over a three‐step telescoped sequence) and similarly high diastereoselectivity (>99:1 dr).

A number of methods were initially evaluated for the reductive desulfonylation of advanced intermediates **7 a** and **7 b**.[^37^, ^38^, ^39^] To our surprise, sonication of fragment **7 a** in THF/EtOH with Na(Hg) amalgam delivered the fragile chiral alcohol **6 a**, which was found to be prone to degradation even on silica.^[38]^ Gratifyingly, EDC‐mediated esterification with acid **19** (see SI for preparation) furnished the stable bisalkyne **4** and set the stage for the envisaged one‐pot ACM–RCAM reaction (Scheme 4). Our synthetic plan for bisalkyne **5** was to follow this route via tosyl group removal to give **6 b** and esterification with 2‐butynoic acid **20**. Unfortunately, sonication of both fragment **7 b**, or its TBS ether **21 b**, in THF/EtOH with Na(Hg) amalgam (2.0–10.0 equiv, 10 mol % Na) either failed completely, or led to instantaneous decomposition. Even the use of SmI_2_ with DMPU, reported to be a particularly mild alternative,^[39]^ only led to the recovery of starting material. This unforeseen subtlety rendered the ynoate–yne one‐pot ACM–RCAM strategy non‐viable and enforced an adjustment to our final synthetic plan.

**Scheme 4:**
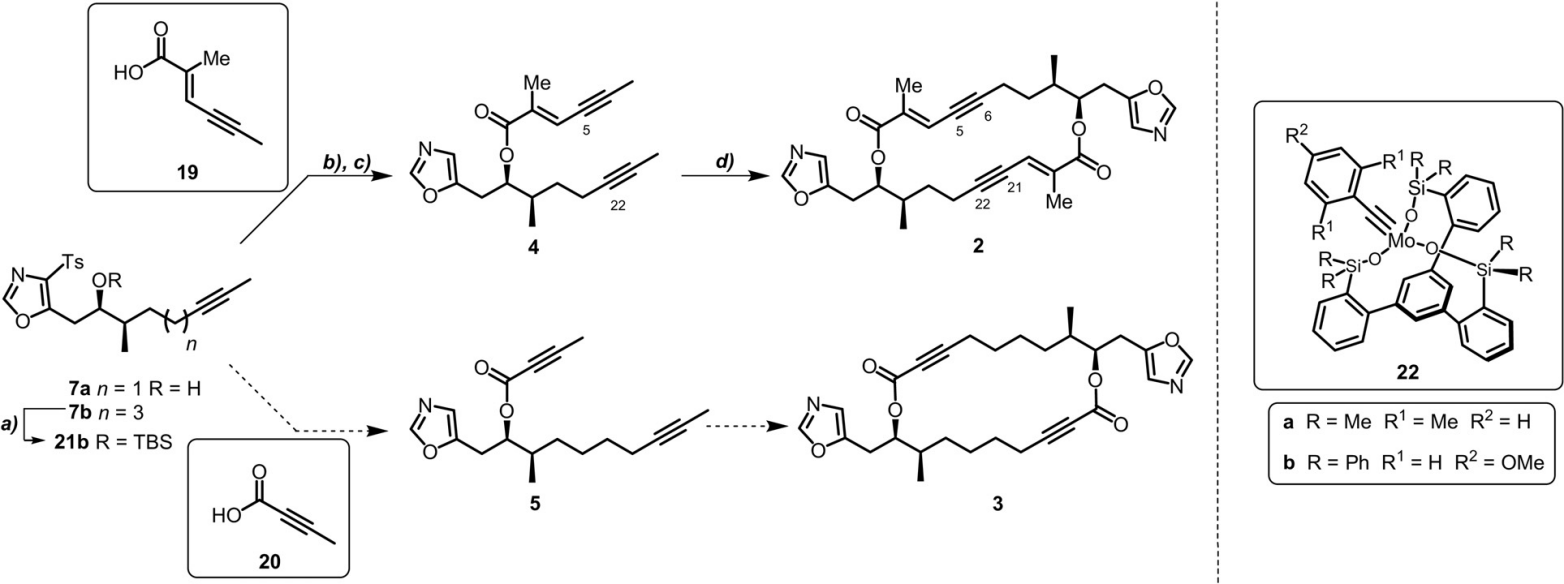
Synthesis of bisalkyne building blocks and bisalkyne cyclic dimers. Reagents and Conditions: a) TBSOTf, 2,6‐lutidine, CH_2_Cl_2_, 0 °C→rt, 18 h, 73 % (**17 b**); b) Na(Hg) (10 mol % Na), Na_2_HPO_4_, THF/EtOH (1:1), 4 h; c) EDC⋅HCl, acid **19**, DMAP (10 mol %), CH_2_Cl_2_, 0 °C→rt, 18 h, 70 % over two steps (**4**); d) see Table 1.

For our one‐pot ACM–RCAM investigation, we employed the two latest‐generation molybdenum alkylidynes (**22 a**/**b**) endowed with the privileged tripodal silanolate ligand framework.[^40^, ^41^] Based on prior work from our group, we treated a solution of bisalkyne **4** in toluene (50 mm) with molybdenum alkylidyne complex **22 a** (10 mol %) at ambient temperature in the presence of molecular sieves (5 Å) to scavenge the released 2‐butyne.^[2]^ Following the reaction by reverse‐phase LC–MS (*λ*=254 nm) indicated no conversion even after four hours at room temperature. However, full conversion to cyclobis(enyne) **2** was attained within two hours by raising the temperature to 60 °C (Table 1, entry 1). Routine chromatography gave the cross‐dimer **2** in analytically pure form in 53 % yield. Although no linear dimers, higher‐order oligomers, nor the corresponding homo‐dimeric isomer were discernible by LC–MS, we nonetheless suspected competing concentration‐induced polymerisation. This undesired pathway, which seems to be independent of the initial catalyst loading (Table 1, entry 2), can be effectively suppressed by performing the reaction at a higher dilution. Indeed, treatment of a more dilute solution of monomer **4** (30 mm) with complex **22 a** (20 mol %) gave a significantly improved yield (Table 1, entry 3). Higher catalyst loading (Table 1, entry 4) and further dilution (Table 1, entry 5) gave no additional improvements. With complex **22 b**, full conversion to the desired one‐pot ACM–RCAM product **2** was only achieved after overnight heating at 80 °C (Table 1, entry 6) and isolation of the product was hindered by fragmentation of the catalyst ligand. These results highlight the remarkable functional group tolerance and robustness of catalyst **22 a**,^[40]^ which activates even the most demanding triple bonds under mild conditions in the presence of basic heterocycles. We suggest that the rigidified backbone and greater steric encumbrance of the coordination sphere of the podand complex **22 b** are responsible for its reduced reactivity, particularly for electron‐deficient substrates such as 1,3‐enynes, which are known to be less reactive.^[41]^

**Table 1:** Optimisation of the one‐pot ACM–RCAM of bisalkyne **4**.

Entry	Catalyst loading [mol %]	*T* [°C]	Molarity [mm]	*t* [h]	Isolated yield of **2** [%]
1^[a]^	10	60	50	2	53
2^[a]^	20	60	50	2	55
**3^[a]^**	**20**	**60**	**30**	**1**	**74**
4^[a]^	30	60	30	1	71
5^[a]^	30	60	15	3	71
6^[b]^	20	80	30	18	50

[a] Reaction conditions: catalyst: complex **22 a**, solvent: toluene, additive: 5 Å MS. [b] Reaction conditions: catalyst: complex **22 b**, solvent: toluene, additive: 5 Å MS.

With the desired 20‐membered macrocyclic framework in hand, our two end game steps could be tackled. Inspired by the recent formal synthesis of the lichen‐derived macrolide (+)‐aspicilin,^[18]^ we envisioned that *trans*‐reduction of the newly formed alkynes to the required *E*‐configured alkenes would be achieved via a bis(*trans*‐hydrostannation–protodestannation) sequence. However, these key steps bore substantial risk, since electron‐rich arenes and 1,3‐enynes often act as catalyst poisons in the *trans*‐hydrostannation reaction.^[42]^ This stems from the ability of these substrates to bind to the [Cp*Ru] fragment through kinetically stable η^4^ or η^6^ complexation, halting further *trans*‐hydrometallation of the alkyne triple bond (**A** and **B**, Scheme 5 (i)).^[43]^ Furthermore, neighbouring group coordination often facilitates both the productive binding of the [Cp*Ru] and the selective proximal delivery of SnBu_3_ to the triple bond (**C** and **D**, Scheme 5 (ii)).^[43]^ In the absence of directing groups, we anticipated that a double hydrostannation reaction would lead to a statistical mixture of proximal and distal stannylated products (**E**, **F** and **G**, Scheme 5 (iii)), all of which derive from a double *trans*‐hydrometallation process and can effectively deliver the desired *E*‐configured alkenes upon protodestannation. In practice, hydrostannation of bis(enyne) **2** with the aid of tetrameric ruthenium catalyst **23** proceeded smoothly at ambient temperature to deliver an ill‐defined mixture of stannylated products, which was not separable by flash chromatography on silica or reverse‐phase HPLC. Therefore, the overall *trans*‐selectivity of the double hydrostannation reaction was only determined after protodestannation had taken place.

**Scheme 5:**
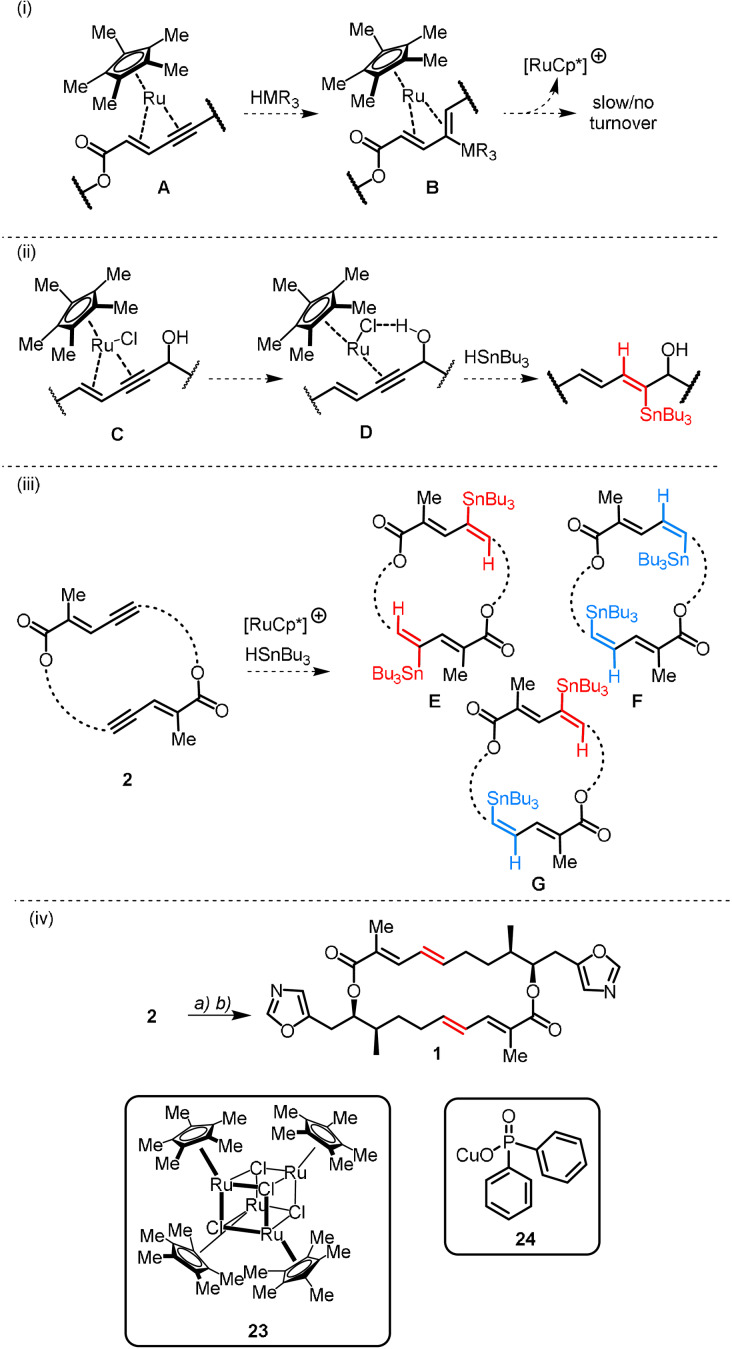
*Trans*‐hydrometallation and end game steps: (i) Unproductive 1,3‐enyne hydrometallation; (ii) Protic‐group‐assisted *trans*‐hydrostannation; (iii) Expected products from the double *trans*‐hydrostannation of bis(enyne) **2**; (iv) Reagents and Conditions: a) tetrameric complex **23** (10 mol %), Bu_3_SnH, CH_2_Cl_2_, rt, 0.5 h; b) **24**, DMF/MeOH (10:1), rt, 2 h, 30 % over two steps after preparative HPLC.

Despite the ease of forging and subsequent elaboration of the macrocyclic core, protodestannation of the alkenylstannanes was particularly taxing. Treatment of the mixture of stannanes with TFA, silica, TBAF or even in situ generated HF, in both coordinating and non‐coordinating media, led either to no conversion or to decomposition. Confronted by this impasse, we resorted to a different approach. Given that most synthetic methodologies available for the downstream functionalisation of alkenylstannanes rely on the reversible transmetalation of tin to copper in polar solvents, we considered protodestannation via the formation of an organocopper species in the presence of a tin scavenger and an exogeneous proton source. After extensive experimentation we found that employing a mixture of DMF/MeOH (10:1) in the presence of copper(I) salt **24** at ambient temperature led to successful protodestannation (Scheme 5 (iv)), furnishing the desired (5*E*,21*E*) isomer (**1**) as the major stereoisomer (crude HPLC ratio 78:22, (**1**):*E*/*Z* isomers). To our satisfaction, the analytical and spectroscopic data of the synthetic sample of **1** matched those of the natural product.

In summary, we have completed the first total synthesis of the anti‐malarial macrodiolide samroiyotmycin A (**1**) (9 steps longest linear sequence, with an overall yield of 6 %). This convergent and scalable route showcases an initial asymmetric alkylation of commercial **17**, followed by a three‐step telescoped sequence to set the C(10)OH stereochemistry and install the 1,3‐oxazole unit, utilising an unprecedented Schöllkopf‐type condensation on enantioenriched β‐lactones. Forging the rigid macrocyclic core was achieved by an efficient one‐pot ACM–RCAM reaction using the latest generation of alkyne metathesis catalysts, while the final elaboration to the desired *C*_2_‐symmetric macrodiolide was possible via a delicate *trans*‐hydrostannation–protodestannation sequence. These results demonstrate that, when combined with contemporary hydrometallation chemistry, the one‐pot ACM–RCAM reaction can be a powerful manifold for the assembly of complex molecular architectures. We anticipate that this synthetic route will provide a solid base for future exploration of other members of this family, and the design of analogues with improved application profiles.

## Conflict of interest

The authors declare no conflict of interest.

## Supporting information

As a service to our authors and readers, this journal provides supporting information supplied by the authors. Such materials are peer reviewed and may be re‐organized for online delivery, but are not copy‐edited or typeset. Technical support issues arising from supporting information (other than missing files) should be addressed to the authors.

Supporting Information
