# Supplementary material for: An Alkyne‐Metathesis‐Based Approach to the Synthesis of the Anti‐Malarial Macrodiolide Samroiyotmycin A
Source: Angew Chem Int Ed Engl. 2021 Jul 20;60(34):18504–8. doi: 10.1002/anie.202105732 (PMC8456858; doi:10.1002/anie.202105732)
Supplement: Supplementary file 1 — Supporting Information [file ANIE-60-18504-s001.pdf]

Angewandte Chemie
Eine Zeitschrift der Gesellschaft Deutscher Chemiker

Supporting Information

An Alkyne-Metathesis-Based Approach to the Synthesis of the Anti-Malarial Macrodiolide Samroiyotmycin A

*Ektoras Yiannakas, Mark I. Grimes, James T. Whitelegge, Alois Fürstner, and Alison N. Hulme**

anie_202105732_sm_miscellaneous_information.pdf

Author Contributions

E.Y. Investigation: Lead; Methodology: Lead; Writing—original draft: Lead
M.G. Investigation: Supporting; Writing—review & editing: Supporting
J.W. Investigation: Supporting; Writing—review & editing: Supporting
A.F. Supervision: Supporting; Writing—review & editing: Supporting
A.H. Conceptualisation: Lead; Funding acquisition: Lead; Supervision: Lead; Writing—review & editing: Lead.

Table of Contents

1. **6-Heptynoic acid (S1)** **3**
2. **5-Iodopent-2-yne (S2)** **3**
3. **7-iodohept-2-yne (S3)** **3**
4. **Non-7-ynoic acid (S4)** **4**
5. **(1*S*,2*S*,4*S*,5*R*)-2-((*R*)-(6-methoxyquinolin-4-yl)((trimethylsilyl)oxy)methyl)-5-vinylquinuclidine (14)** **5**
6. **(*E*)-2-methylhex-2-en-4-ynoic acid (19)** **6**
7. **(*R*)-4-benzyl-3-(hept-5-ynoyl)oxazolidin-2-one (10a)** **6**
8. **(*R*)-4-benzyl-3-((*R*)-2-methylhept-5-ynoyl)oxazolidin-2-one (11a)** **9**
9. **(2*R*,3*R*)-3-methyl-1-(4-tosyloxazol-5-yl)oct-6-yn-2-ol (7a)** **12**
10. **(*R*)-2-methylhept-5-ynoic acid (12a)** **12**
11. **(R)-N-nethoxy-N,2-diemthylhept-5-ynamide (13a)** **17**
12. **(*R*)-*N*-((1*S*,2*S*)-1-hydroxy-1-phenylpropan-2-yl)-*N*,2-dimethylhept-5-ynamide (18a)** **25**
13. **5-((2*R*,3*R*)-3-methyl-2-((*R*)-2,2,2-trifluoro-1-methoxy-1-phenylethoxy)oct-6-yn-1-yl)-4-tosyloxazole (15a)** **33**
14. **5-((2R,3R)-3-methyl-2-((S)-2,2,2-trifluoro-1-methoxy-1-phenylethoxy)oct-6-yn-1-yl)-4-tosyloxazole (16a)** **33**
15. **(*R*)-*N*-((1*S*,2*S*)-1-hydroxy-1-phenylpropan-2-yl)-*N*,2-dimethylnon-7-ynamide (18b)** **39**
16. **(*R*)-4-benzyl-3-((*R*)-2-methylnon-7-ynoyl)oxazolidin-2-one (11b)** **48**
17. **(2*R*,3*R*)-3-methyl-1-(4-tosyloxazol-5-yl)dec-8-yn-2-ol (7b)** **51**
18. **(2*R*,3*R*)-3-methyl-1-(4-tosyloxazol-5-yl)dec-8-yn-2-yl-(R)-3,3,3-trifluoro-2-methoxy-2-phenyl propanoate (15b)** **65**
19. **(2*R*,3*R*)-3-methyl-1-(4-tosyloxazol-5-yl)dec-8-yn-2-yl-(*S*)-3,3,3-trifluoro-2-methoxy-2-phenyl propanoate (16b)** **65**
20. **Table S-2. Mosher ester analysis of (2*R*,3*R*)-3-methyl-1-(4-tosyloxazol-5-yl)dec-8-yn-2-ol 7b, arbitrary numbering scheme as shown in the insert.** **70**
21. **5-((2*R*,3*R*)-2-((*tert*-butyldimethylsilyl)oxy)-3-methyldec-8-yn-1-yl)-4-tosyloxazole (21b)** **70**
22. **(2*R*,3*R*)-3-methyl-1-(oxazol-5-yl)oct-6-yn-2-yl (*E*)-2-methylhex-2-en-4-ynoate (4):** **73**
23. **(3*E*,9*R*,10*R*,13*E*,19*R*,20*R*)-3,9,13,19-tetramethyl-10,20-bis(oxazol-5-ylmethyl)-1,11-dioxacycloicosa-3,13-dien-5,15-diyne-2,12-dione (2):** **76**
24. **Samroiyotmycin A (1)** **79**
25. **Table S-4. Assignment of ^{1}H & ^{13}C NMR spectra of synthetic *Samroiyotmycin A*.** **79**
26. **Figure S-3. Evidence for the selectivity of observed during the bis (trans-reduction) of macrocycle 2** **80**
27. **Table S-5. Comparison of NMR data between the isolate and synthetic *Samroiyotmycin A*.** **81**
28. **References** **89**

GENERAL

Unless stated otherwise, all reactions were carried out under Ar in flame-dried glassware. The solvents used were purified by distillation over the drying agents indicated and were transferred under Ar: THF, Et_2O (Mg/anthracene), CH_2Cl_2, DMF (CaH_2), toluene (Na/K). Flash chromatography on silica gel (FC): Merck silica gel 60 (230–400 mesh). NMR: Spectra were acquired on Bruker AvanceIII 300, 400, 500 MHz and an Avance Neo 600 MHz NMR spectrometers in the solvents indicated; chemical shifts (δ) are given in ppm relative to TMS, coupling constants (J) in Hz. The solvent signals were used as references and the chemical shifts converted to the TMS scale ($CDCl_3$: δ_C = 77.0 ppm; residual $CHCl_3$ in $CDCl_3$: δ_H = 7.26 ppm; CD_3OD: δ_C = 49.00 ppm, residual CD_2HOD in CD_3OD: δ_H = 3.31 ppm; $(CD_3)_2SO$: δ_C = 39.52 ppm, residual CD_2HSOCD_3 in $(CD_3)_2SO$: δ_H = 2.50 ppm). IR: Spectrum One (Perkin-Elmer) spectrometer, wavenumbers ($\tilde{\nu}$) in cm^{-1}. MS (EI): Finnigan MAT 8200 (70 eV), ESI-MS: ESQ3000 (Bruker), accurate mass determinations: Bruker APEX III FT-MS (7 T magnet) or Mat 95 XP (Finnigan). All commercially available compounds (Fluka, Lancaster, Aldrich, Fluorochem, Alfar Aeser, TCI, ChemPUR, Santa Cruz Biotechonology) were used as received, unless stated otherwise. The molecular sieves used in this investigation were dried for 24 h at 150°C (sand bath) under vacuum prior to use and were stored and transferred under argon atmosphere. Data acquired on the AVneo 600 MHz NMR spectrometer was acquired with a Bruker BBO CryoProbe, which significantly reduced the measurement time of most of the spectra, especially the 1D ^{13}C NMR data. Optical rotations were measured with a PerkinElmer Model 343 polarimeter. Melting points were recorded on a Buchi B-540 melting point apparatus. LC-MS analyses were conducted on a Shimadzu instrument (pump LC-20AD, autosampler SIL-20AC, column oven CTO-20AC, diode array detector SPD-M20A, controller CBM-20A, ESI detector and software Labsolutions) with an ZORBAX Eclipse Plus C_{18} (1.8 µm, 3.0 or 4.6 mm ID × 50 mm) (Agilent). A binary gradient of MeCN or MeOH in water or aq. ammonium bicarbonate buffer (20 mmol. pH 9) was used at a flow rate of 0.5 (3.0 mm ID) or 0.8 (4.6 mm ID) mL/min. The oven temperature was kept at 35 °C and the detection wavelength at 254 nm. Preparative LC was performed with a Shimadzu LC-20A prominence system (pump LC-20AP, column oven CTO-20AC, diode array detector SPD-M20A, fraction collector FRC-10A, controller CBM-20A and software LC-solution); conditions for each compound are specified below.

6-Heptynoic acid (S1)

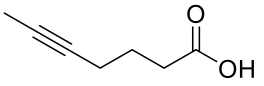

A two-neck round round-bottomed flask equipped with a magnetic stir bar, a reflux condenser and a gas inlet connected to an argon-vacuum manifold, was charged with solution of 6-heptynoic acid (6.4 g, 48.0 mmol) in DMSO (89 mL) and KO^{t}Bu (11.3 g, 101 mmol, 2.1 equiv.). The reaction mixture was heated at 85 °C for 0.5 h. After that, the reaction mixture was allowed to cool to rt and then quenched with HCl (100 mL, 1 M, aq). The resulting mixture was extracted with Et_2O (3 x 100 mL). The combined organic extracts were dried over $MgSO_4$, filtered and the concentrated under reduced pressure. The residue obtained was purified by flash column chromatography (SiO_2, 100 g, 90 mm ⌀, 0-50% EtOAc/iso-Hexane , ca. 20 mL fractions). This gave the product as white crystalline solid (4.6 g, 76 %). ^{1}H NMR (400 MHz, $CDCl_3$): δ = 11.0 (brs, 1H), 2.49 (t, J = 7.4 Hz, 2H), 2.22 (tq, J = 7.4, 2.5 Hz, 2H), 1.86 – 1.79 (m, 2H), 1.77 (t, J = 2.5 Hz, 3H); ^{13}C NMR (101 MHz, $CDCl_3$) δ 178.0, 77.7, 77.2, 33.9, 23.9, 18.1, 3.4. The spectroscopic data are in agreement with those reported in the literature.[1]

5-Iodopent-2-yne (S2)

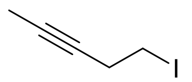

A tin-foil covered one-neck round-bottomed flask equipped with a stir bar, was charged with a solution of 4-pentyn-1-ol (5.0 g, 59.4 mmol) in Et_2O/MeCN (3:1, 300 mL), imidazole (6.1 g. 89.2 mmol, 1.5 equiv.) and PPh_3 (23.4 g, 89.2 mmol, 1.5 equiv.). The resulting mixture was cooled to 0 °C and iodine (22.6 g, 89.2 mmol, 1.5 equiv) was added in the small portions over 5 mins. The reaction mixture was then allowed to warm to rt and stirred for 2 h. The reaction mixture was then diluted with Et_2O (240 mL) and quenched with $NaHCO_3$ (150 mL, sat. aq.). The aqueous phase was then extracted with with Et_2O (2 x 150 mL) and then combined organic extracts were washed with $Na_2S_2O_3$ (200 mL, sat.aq.), dried over $MgSO_4$, filtered and then concentrated under reduced pressure (300 mbar) at rt in the abscence of light. The residue obtained was purified by flash column chromatography (300 g, SiO_2, 110 mm Ø, pentane). This gave the desired product as a pale yellow oil (10.5 g, 91 %). ^{1}H NMR (400 MHz, $CDCl_3$): δ = 3.20 (t, J = 7.4 Hz, 2H), 2.72 (tq, J = 7.4, 2.5 Hz, 2H), 1.78 (t, J = 2.5 Hz, 3H); ^{13}C NMR (101 MHz, $CDCl_3$) δ 77.93, 77.82, 24.14, 3.55, 2.58. The spectroscopic data are in agreement with those reported in the literature.[2]

7-iodohept-2-yne (S3)

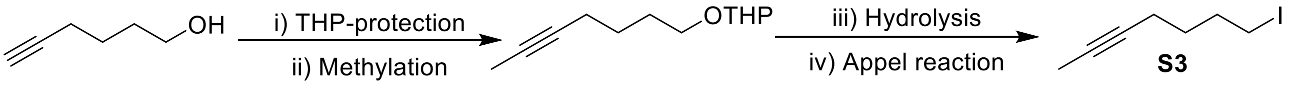

<u>Step 1 (THP-protection):</u>

A three-neck round bottom flask equipped with a stir bar, an argon bridge and a rubber septum, was charged with TsOH (184 mg, 1.0 mmol, 1.0 mol%) and a solution of 5-hexyn-1-ol (10.0 g, 96.8 mmol) in CH_2Cl_2 (125 mL). The flask was cooled to 0 °C over an ice-bath and 3,4-DHP (10.0 mL, 106 mmol, 1.1 equiv.) was added dropwise. The resulting mixture was allowed to warm to rt and then stirred overnight. After that the reaction mixture was directly passed through a silica plug (SiO_2, 100 g, 120 mm Ø, CH_2Cl_2, ca. 75 mL fractions). Fractions containing the desired protected alcohol were combined and concentrated under reduced pressure. This gave the THP-ether as a colourless oil (17.1 g). The crude THP-ether was used in the next step without any further purification.

<u>Step 2 (Methylation):</u>

A three-neck round bottom flask equipped with a stir bar, an argon bridge and a pressure equalising dropping funnel, was charged with a solution of the crude THP-ether (17.1 g, 96.8 mmol) in THF (345 mL). The reaction mixture was cooled to - 78 °C and ^{n}BuLi (79.2 mL, 127 mmol, 1.6 M, 1.05 equiv., in *n*-hexanes) was added dropwise. After stirring at - 78 °C for 1 h, MeI (9.0 mL, 145 mmol, 1.5 equiv.) was

added dropwise and the resulting mixture was allowed to warm to rt overnight. After that the reaction mixture was quenched with brine (1000 mL) and then extracted with Et_2O (3 x 250 mL). The combined organic extracts were dried over $MgSO_4$, filtered and then concentrated under reduced pressure. This gave the desired product as a pale orange oil (13.8 g), which was utilized in the next step without purification.

Step 3 (Hydrolysis):

A two-neck round bottom flask equipped with an argon bridge, a stir bar and a rubber septum, was charged with a solution of the crude THP-protected alcohol in MeOH (150 mL) and TsOH (368 mg, 2.0 mmol, 2.0 mol%). The reaction mixture was stirred overnight at rt. After that the reaction mixture was quenched with $NaHCO_3$ and then diluted with H_2O (850 mL). The resulting mixture was extracted with Et_2O (3 x 250 mL) and the combined organic extracts were dried over $MgSO_4$, filtered and then concentrated under reduced pressure. This afforded the alcohol as a colourless oil (10.0 g), which was used in the next step without purification.

Step 4 (Appel Reaction):

A tin-foil wrapped round bottom flask equipped with a stir bar was charged with a solution of the alcohol in Et_2O/MeCN (3:1, 400 mL) was added along with PPh_3 (38.1 g, 145 mmol, 1.5 equiv.) and imidazole (9.8 g, 145 mmol, 1.5 equiv.). The mixture was cooled to 0 ^oC over an ice-bath and iodine (36.8 g, 145 mmol, 1.5 equiv.) was added portion wise. After that, the cooling bath was removed and resulting mixture was stirred at rt for 2 h. The reaction mixture was then diluted with Et_2O (600 mL), quenched with $NaHCO_3$ (100 mL, sat. aq.) and washed twice with $Na_2S_2O_3$ (2 x 200 mL, sat. aq.). The organic phase was then dried over $MgSO_4$, filtered and then concentrated under reduced pressure at rt. The residue obtained was then passed through a silica plug (SiO_2, 100 g, 120 mm Ø, *n*-pentane, ca. 75 mL fractions). This afforded alkyl iodide **21** as a colourless oil (20.5 g, 95 % over four steps). ^{1}H NMR (400 MHz, $CDCl_3$): δ = 3.14 (t, *J* = 7.8 Hz, 2H), 2.09 (tp, *J* = 7.8, 2.6 Hz, 2H), 1.92 – 1.81 (m, 2H), 1.71 (t, *J* = 2.6 Hz, 3H), 1.57 – 1.45 (m, 2H); ^{13}C NMR (101 MHz, $CDCl_3$): δ = 78.8, 76.6, 32.9, 30.2, 18.2, 6.8, 3.9. The spectroscopic data are in agreement with those reported in the literature.[2]

Non-7-ynoic acid (S4)

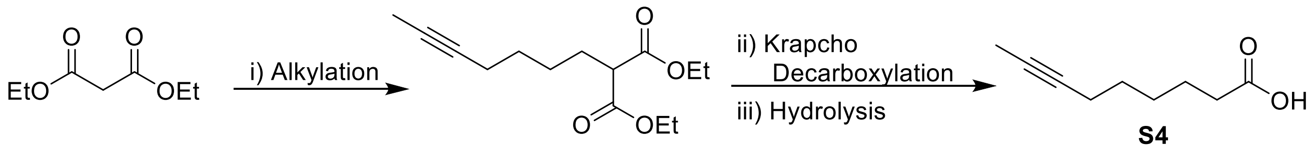

S4

Step 1 (Alkylation):

A two-neck round bottom flask equipped with a stir bar, rubber septum and an argon bridge was charged with NaH (813 mg, 33.9 mmol, 1.05 equiv.) and DMF (89 mL). The resulting suspension was cooled to 0°C over an ice-bath. Diethyl malonate (5.3 g, 32.3 mmol) was added, and the resulting mixture was stirred for 0.5 h at 0 °C. After that 7-iodohept-2-yne **S3** (7.5 g, 33.9 mmol, 1.1 equiv.) was added and reaction mxture was allowed to warm to rt and then stirred overnight. The reaction mixture was quenched with brine (400 mL) and then extracted with Et_2O (4 x 125 mL). The combined organic extracts were then dried over $MgSO_4$, filtered and then concentrated under reduced pressure. The yellow oil obtained was carried forward without any purification.

Step 2 (Krapcho Decarboxylation):

A round bottom flask equipped with a reflux condenser and a stir bar was charged with a solution of the crude diethyl 2-(hept-5-yn-1-yl)malonate in DMSO (57 mL), water (232 uL, 12.8 mmol, 0.4 equiv.) and LiCl (3.0 g, 71.3 mmol, 2.2 equiv.). The resulting mixture was heated at 160 °C overnight. Upon cooling to rt, the reaction mixture was diluted with brine (600 mL) and extracted with Et_2O (3 x 150 mL). The combined organic extracts were dried over $MgSO_4$, filtered and then concentrated under reduced pressure. The brown oil obtained was then carried foward without any purification.

Step 3 (Hydrolysis):

A round bottom flask equipped with a stir bar was charged with a solution of the crude ethyl non-7-ynoate in THF (115 mL) and the flask was cooled to 0 °C. A solution of LiOH (2.3 g, 96.8 mmol, 3.0 equiv.) in water (115 mL) was added and the resulting mixture was allowed to warm to rt and then stirred overnight. The THF was removed under reduced pressure and the aqueous fraction obtained was acidified to pH 1 with conc. HCl. The resulting mixture was extracted with EtOAc (3 x 100 mL) and the combined organic extracts were dried over $MgSO_4$, filtered and then concentrated under reduced pressure. This gave the acid **S4** as a white solid (4.0 g, 80 % over three steps). ^1H NMR (400 MHz, $CDCl_3$): δ = 2.36 (t, *J* = 7.5 Hz, 2H), 2.17 – 2.09 (m, 2H), 1.77 (t, *J* = 2.5 Hz, 3H), 1.69 – 1.60 (m, 2H), 1.54 – 1.38 (m, 2H); ^{13}C NMR (101 MHz, $CDCl_3$): δ = 180.7, 79.4, 76.1, 34.4, 29.1, 28.6, 24.7, 18.9, 3.9; mp: 50-51 °C; (IR (film): $\tilde{\nu}$ = 2936, 2907, 2858, 1688, 1468, 1439, 1410, 1298, 1275,1252, 1206, 1198, 918 cm^{-1}; HRMS-EI (*m/z*): calc'd. for $C_9H_{14}O_2[M]^{+\cdot}$, 155.1066; found, 155.1064. The spectroscopic data are in agreement with those reported in the literature.[3]

(1*S*,2*S*,4*S*,5*R*)-2-((*R*)-(6-methoxyquinolin-4-yl)((trimethylsilyl)oxy)methyl)-5-vinylquinuclidine (14)

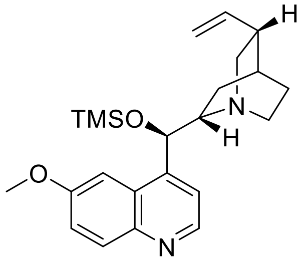

A flame dried two neck round bottomed flask equipped with with a magnetic stir bar and a gas inlet connected to an argon-vacuum manifold, was charged with a solution of quinine (3.0 g, 9.2 mmol) in CH_2Cl_2 (90 mL) and TEA (3.8 mL, 27.5 mmol, 3.0 equiv.) . TMSCl (1.4 mL, 11.0 mmol, 1.2 equiv.) was added dropwise and the resulting mixture was stirred overnight. The reaction mixture was then quenched with $NaHCO_3$ (40 mL, sat. aq.) and the aqueous layer was extracted with CH_2Cl_2 (3 x 30 mL). The combined organic extracts were then dried over $MgSO_4$, filtered and then concetrated under reduced pressure. The residue obtained was purified by flash column chromatography (SiO_2, 100 g, 55 mm ø, 0-30 % MeOH/EtOAc, ca. 20 mL). This gave the product as a white powdery solid (3.0 g, 83 %). 1H NMR (400 MHz, DMSO): δ = 8.67 (s, 1H), 7.93 (d, *J* = 9.2 Hz, 1H), 7.62 – 7.20 (m, 2H), 5.83 (d, *J* = 17.3 Hz, 1H), 5.48 (s, 1H), 5.07 – 4.80 (m, 2H), 3.89 (s, 3H), 3.25 – 2.61 (m, 2H), 2.40 – 2.05 (m, 2H), 1.93 – 1.07 (m, 5H), -0.05 (s, 9H). 13C NMR (101 MHz, DMSO): δ = 173.4, 157.9, 147.4, 144.8, 143.7, 141.7, 131.8, 127.0, 121.8, 118.8, 114.5, 101.4, 73.5, 59.1, 56.5, 55.6, 42.4, 39.6, 27.74, 27.72, 27.5, 24.3, 9.0. The spectroscopic data are in agreement with those reported in the literature.[4]

(*E*)-2-methylhex-2-en-4-ynoic acid (19)

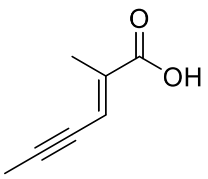

A two-neck round round-bottomed flask equipped with a magnetic stir bar with a magnetic stir bar, a glass stopper and a gas inlet connected to an argon-vacuum manifold equipped with a bubbler containing aqueous KOH, was charged with solution of 2-butyn-1-ol (4.0 mL, 52.0 mmol) in CH_2Cl_2 (350 mL) and aq. pH 8.6 buffer (14.7 g of $NaHCO_3$ and 2.4 g of K_2CO_3 in 350 mL of H_2O). TBACl (1.5 g, 5.2 mmol, 10 mol%), TEMPO (826 mg, 5.2 mmol, 10 mol%) and NCS (11.4 g, 82.3 mmol, 1.6 equiv.) were successively added. The resulting biphasic mixture was vigorously stirred at rt for 7 h. The aqueous layer was extracted with CH_2Cl_2 (300 mL), the combined organic layers were washed with brine, dried over $MgSO_4$ and then concentrated under reduced pressure (300 mbar) at rt. This gave but-2-ynal as a light orange liquid (1.75 g, 50 %), which was directly engaged in the next step. ^{1}H NMR (400 MHz, CD_2Cl_2): δ = 9.13 (q, *J* = 1.0 Hz, 1H), 2.07 (d, *J* = 1.0 Hz, 3H). The spectroscopic data are in agreement with those reported in the literature. [5]

A three-neck jacketed vessel equipped with a magnetic stir bar, a gas inlet connected to an argon-vacuum manifold, a ground-glass joint thermometer adapter connected to low temperature thermometer and a rubber septum was charged with THF (45 mL). The reaction vessel was cooled to – 60 °C and ^{n}BuLi (9.2 mL, 14.6 mmol, 2.0 equiv.) was added. A solution of 2-(diethoxyphosphoryl)propanoic acid (1.5 g, 7.8 mmol, 1.0 equiv.) in THF (7 mL) was added dropwise and the mixture was stirred at – 60 °C for 0.5 h. A solution of the crude but-2-ynal in THF (10 mL) was added and the resulting mixture was stirred at – 60 °C for 0.5 h, before it was warmed to rt and then stirred overnight. The reaction mixture was then quenched with dilute HCl (20 mL, 1.0 M, aq.) and extracted with Et_2O (3 x 100 mL). The combined organic yellow slurry obtained was then diluted with water (100 mL) and extracted with EtOAc (3 x 150 mL). The combined organic layers were dried over $MgSO_4$, filtered and then concentrated under reduced pressure. The residue was purified by flash column chromatography (SiO_2, 100 g, 55 mm ø, 0-40% EtOAc/iso-Hexanes, ca. 20 mL). This gave the product as a white solid (573 mg, 63 %).^{1}H NMR (400 MHz, $CDCl_3$): δ = 9.81 (brs, 1H), 6.70 (qt, *J* = 2.5, 1.3 Hz, 1H), 2.09 (dd, *J* = 2.5, 0.7 Hz, 3H), 2.04 (dd, *J* = 1.3, 0.7 Hz, 3H); ^{13}C NMR (101 MHz, $CDCl_3$): δ = 171.6, 136.9, 122.9, 100.8, 77.2, 14.9, 5.1. The spectroscopic data are in agreement with those in the literature.[6]

(*R*)-4-benzyl-3-(hept-5-ynoyl)oxazolidin-2-one (10a)

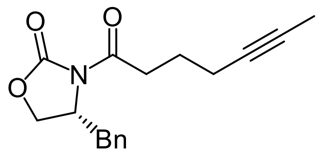

A three-neck jacketed glass reaction vessel equipped with a magnetic stir bar, a pressure equalizing addition funnel and a gas inlet connected to an argon-vacuum manifold, was charged with solution of 6-heptynoic acid **S1** (4.6 g, 36.5 mmol) in THF (100 mL) and TEA (13.3 mL, 38.7 mmol, 1.1 equiv.). The reaction mixture was then cool to - 10°C and PvCl (4.4 mL, 35.8 mmol, 1.1 equiv.) dropwise. The white slurry immediately formed was stirred at this temperature for 1 h. Then, LiCl (1.7 g, 39.9 mmol, 1.2 equiv.) and (R)-4-(phenylmethyl)-2-oxazolidinone (6.2 g, 34.1 mmol) were added in one portion. The resulting mixture was allowed to warm to rt and then stirred overnight. The reaction mixture was quenched with $NaHCO_3$ (200 mL, sat. aq.) and extracted with EtOAc (3 x 150 mL). The combined organic layers were washed with brine (150 mL), dried $MgSO_4$, filtered and then concentrated under reduced pressure. The residue was purified by flash column chromatography (SiO_2, 100 g, 55 mm ø, 0-40% EtOAc/iso-Hexanes, ca. 20 mL). This gave the product as a pale yellow oil (7.0 g, 72 %).^{1}H NMR (400 MHz, $CDCl_3$): δ = 7.36 – 7.31 (m, 2H), 7.30 – 7.25 (m, 1H), 7.23 – 7.17 (m, 2H), 4.67 (ddt, *J* = 9.6, 7.4, 3.4 Hz, 1H), 4.26 – 4.13 (m, 2H), 3.30 (dd, *J* = 13.3, 3.4 Hz, 1H), 3.16 – 2.97 (m, 2H), 2.77 (dd, *J* = 13.3, 9.6 Hz, 1H), 2.26 (tq, *J* = 7.4, 2.5 Hz, 2H), 1.93 – 1.82 (m, 2H), 1.78 (t, *J* = 2.5 Hz, 3H);^{13}C NMR (101 MHz, $CDCl_3$): δ = 173.3, 153.9, 135.8, 129.9, 129.4, 127.8, 78.5, 76.9, 66.5, 55.8, 38.4, 34.9, 23.9, 18.6, 3.9; $[\alpha]_D^{20}$= – 59.1 (c = 1.13, $CHCl_3$); IR (film): $\tilde{\nu}$ = 3029, 2919,1779,1699, 1604, 1631, 1496, 1480, 1453, 1386, 1352, 1324, 1290, 1251, 1210, 1110, 1076, 1052, 1012, 932, 843, 762, 748, 703, 666. 671, 621, 595, 506, 438, 416 cm-1; HRMS-ESI+ (*m/z*): calc'd. for $C_{17}H_{20}N_1O_3$ [M+H]$^{+,}$ 286.1436; found, 286.1437.

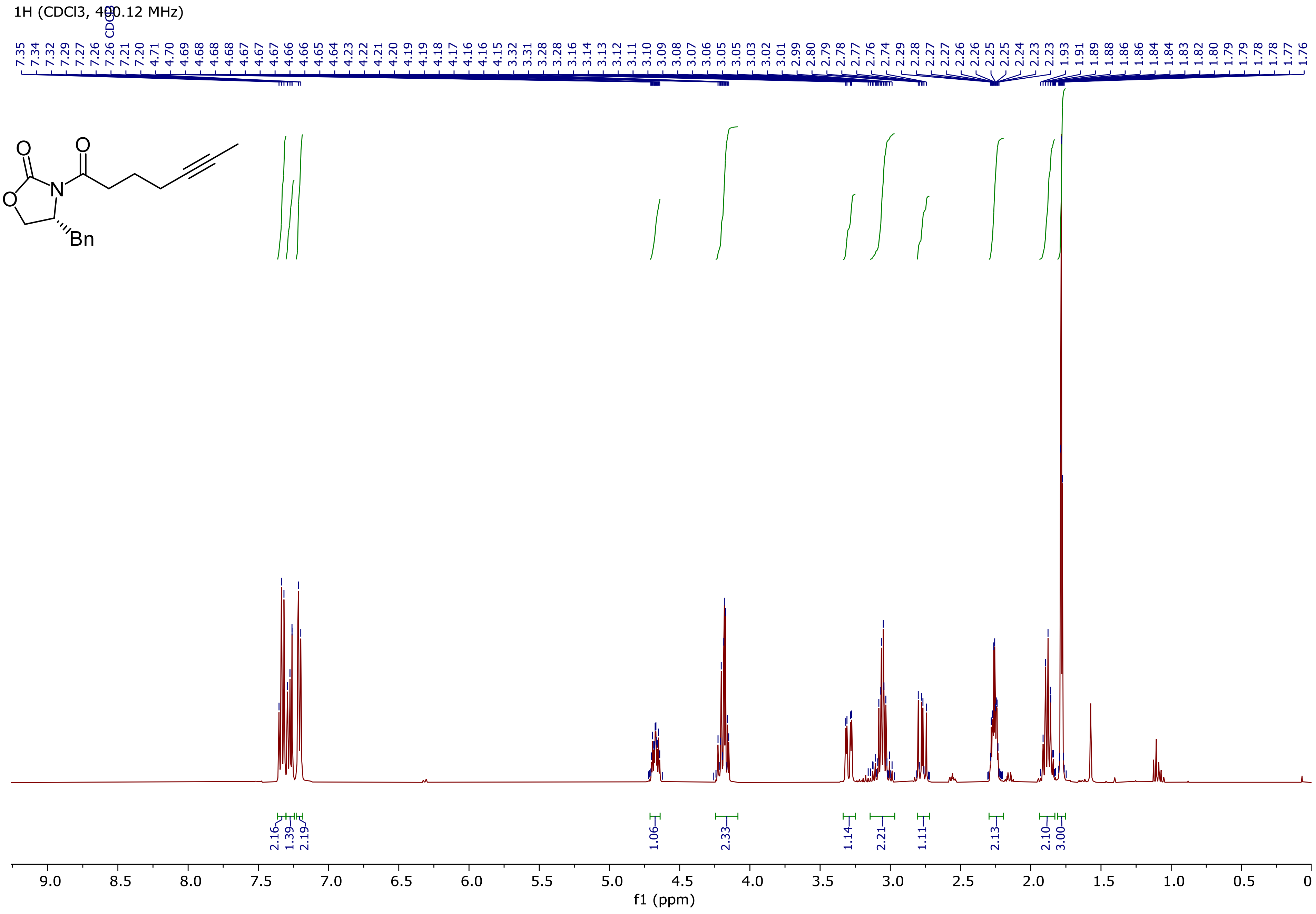
1H (CDCl3, 400.12 MHz)
CDCl3
7.35 7.34 7.32 7.29 7.27 7.26 7.21 7.20 4.71 4.70 4.69 4.68 4.68 4.68 4.67 4.67 4.66 4.66 4.65 4.64 4.23 4.22 4.21 4.20 4.19 4.19 4.18 4.17 4.16 4.16 4.15 3.32 3.31 3.28 3.16 3.14 3.13 3.12 3.11 3.10 3.09 3.08 3.07 3.06 3.05 3.05 3.03 3.02 3.01 2.99 2.80 2.79 2.78 2.77 2.76 2.74 2.29 2.28 2.27 2.27 2.26 2.26 2.25 2.25 2.24 2.23 2.23 1.93 1.91 1.89 1.88 1.86 1.84 1.84 1.83 1.82 1.80 1.79 1.78 1.78 1.77 1.76
O
O
N
O
Bn
2.16 1.39 2.19 1.06 2.33 1.14 2.21 1.11 2.13 2.10 3.00
9.0 8.5 8.0 7.5 7.0 6.5 6.0 5.5 5.0 4.5 4.0 3.5 3.0 2.5 2.0 1.5 1.0 0.5 0
f1 (ppm)

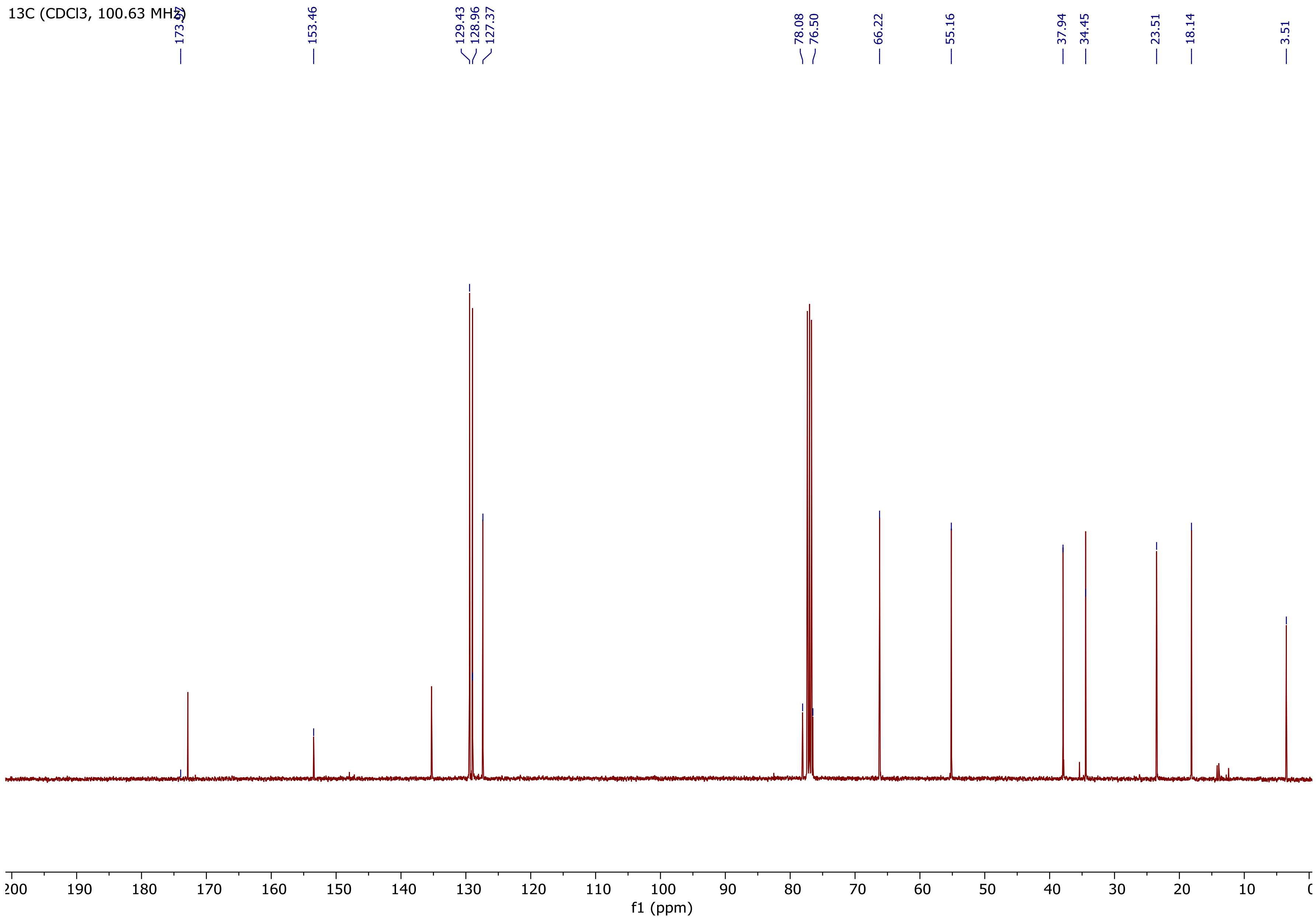
13C (CDCl3, 100.63 MHz)
173.?
153.46
129.43
128.96
127.37
78.08
76.50
66.22
55.16
37.94
34.45
23.51
18.14
3.51
f1 (ppm)

(*R*)-4-benzyl-3-((*R*)-2-methylhept-5-ynoyl)oxazolidin-2-one (11a)

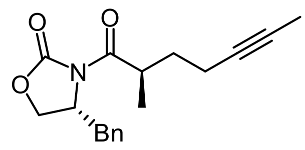

A three-neck jacketed vessel equipped with a stir bar, a gas inlet connected to an argon-vacuum manifold, a ground-glass joint thermometer adapter connected to low temperature thermometer and a rubber septum was charged with a solution of (*R*)-4-benzyl-3-(hept-5-ynoyl)oxazolidin-2-one **10a** (2.0 g, 7.0 mmol) in THF (40 mL). The reaction mixture was cooled to –78 °C. A solution of NaHMDS (7.1 mL, 10.5 mmol, 1.5 equiv., 1.5 M solution in THF) was added dropwise. The resulting pale yellow mixture was stirred for 1 h at - 78 °C. The reaction mixture was then treated with MeI (2.2 mL, 35.0 mmol, 5.0 equiv.) and stirred for additional 4.5 h at -78 °C. The yellow reaction was quenched with AcOH (4.0 mL) and warmed up to room temperature. The yellow slurry obtained was then diluted with water (100 mL) and extracted with EtOAc (3 x 150 mL). The combined organic layers were dried over MgSO4, filtered and then concentrated under reduced pressure. The residue was purified by flash column chromatography (SiO_2, 100 g, 55 mm ø, 0-40% EtOAc/iso-Hexanes, ca. 20 mL). This gave the product as a colourless oil (2.27 g, 77 %). ^{1}H NMR (400 MHz, $CDCl_3$) δ 7.36 – 7.31 (m, 2H), 7.30 – 7.26 (m, 1H), 7.24 – 7.19 (m, 2H), 4.68 (ddt, *J* = 9.6, 7.4, 3.2 Hz, 1H), 4.25 – 4.13 (m, 2H), 3.93 – 3.80 (m, 1H), 3.26 (dd, *J* = 13.3, 3.2 Hz, 1H), 2.77 (dd, *J* = 13.3, 9.6 Hz, 1H), 2.25 – 2.08 (m, 2H), 1.97 (ddt, J = 13.3, 7.4, 6.9 Hz, 1H), 1.75 (t, *J* = 2.5 Hz, 3H), 1.68 – 1.56 (m, 1H), 1.33 – 1.16 (m, 3H); ^{13}C NMR (101 MHz, $CDCl_3$) δ 176.7, 153.0, 135.3, 129.5, 128.9, 127.4, 78.1, 76.4, 66.1, 55.3, 37.9, 36.9, 32.3, 17.5, 16.6, 3.5; $[\alpha]_D^{20}$= – 47.3 (*c* = 1.02, $CHCl_3$); IR (film): $\tilde{\nu}$ = 3030, 2971, 2920, 2857, 2857, 1779, 1696, 1604, 1481, 1481, 1455, 1386, 1350, 1290, 1247; 1212, 1105, 1075, 1049, 1015, 974, 924, 762, 746, 703, 507 cm^{-1}; HRMS-EI (*m/z*): calc'd. for $C_{18}H_{21}NO_3$ $[M]^{+\cdot}$, 299.1515; found, 299.1515.

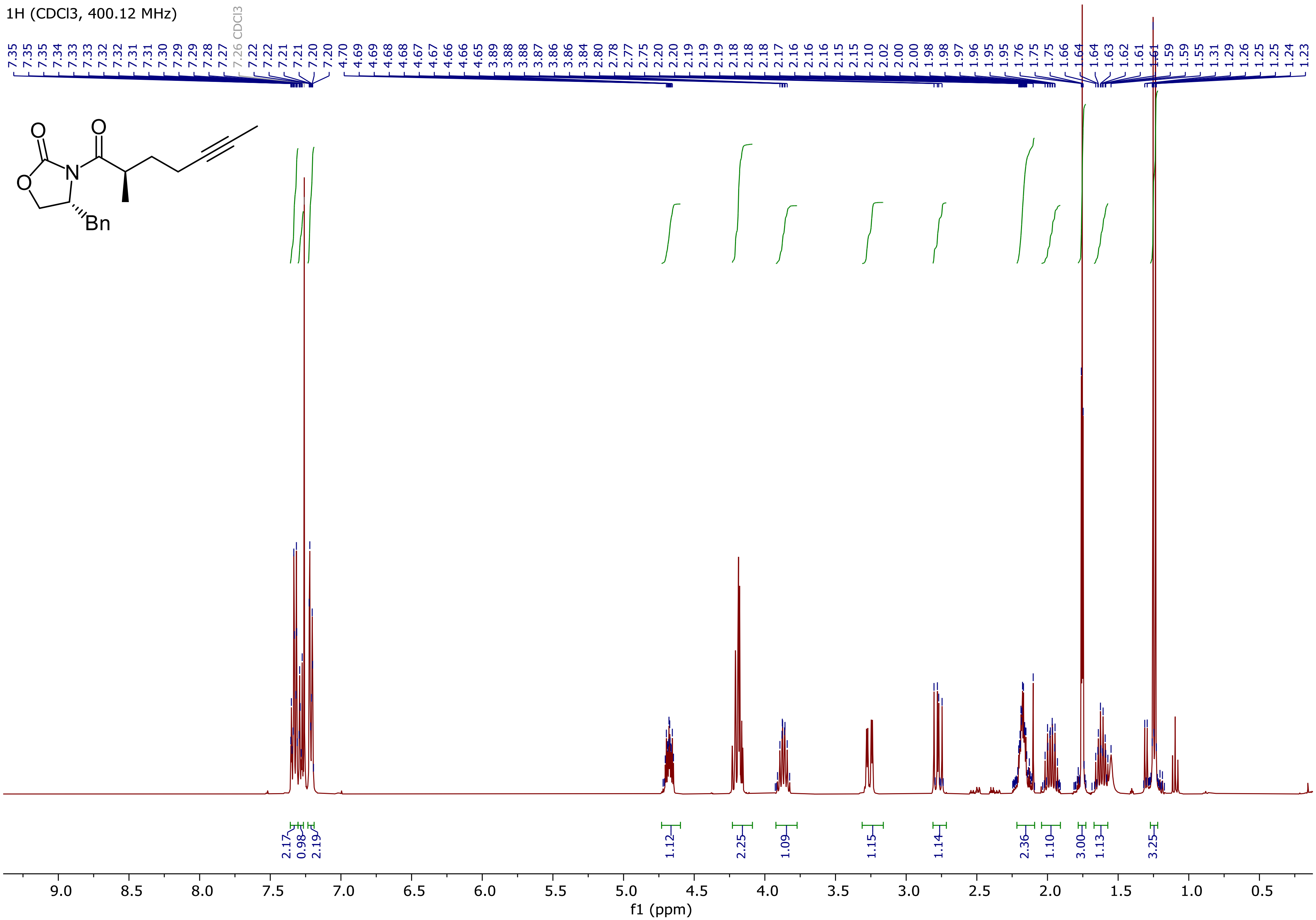
1H (CDCl3, 400.12 MHz)
7.35 7.35 7.35 7.34 7.33 7.33 7.32 7.32 7.31 7.31 7.30 7.29 7.29 7.28 7.27 7.26 CDCl3 7.22 7.22 7.21 7.21 7.20 7.20 4.70 4.69 4.69 4.68 4.67 4.67 4.66 4.65 3.89 3.88 3.88 3.87 3.86 3.86 3.84 2.80 2.78 2.77 2.75 2.20 2.20 2.19 2.19 2.19 2.18 2.18 2.18 2.17 2.16 2.16 2.16 2.15 2.15 2.10 2.02 2.00 1.98 1.98 1.97 1.96 1.95 1.95 1.76 1.75 1.75 1.66 1.64 1.64 1.63 1.62 1.61 1.61 1.59 1.59 1.55 1.31 1.29 1.26 1.25 1.25 1.24 1.23
O
O
N
O
Bn
2.17 0.98 2.19 1.12 2.25 1.09 1.15 1.14 2.36 1.10 3.00 1.13 3.25
9.0 8.5 8.0 7.5 7.0 6.5 6.0 5.5 5.0 4.5 4.0 3.5 3.0 2.5 2.0 1.5 1.0 0.5
f1 (ppm)

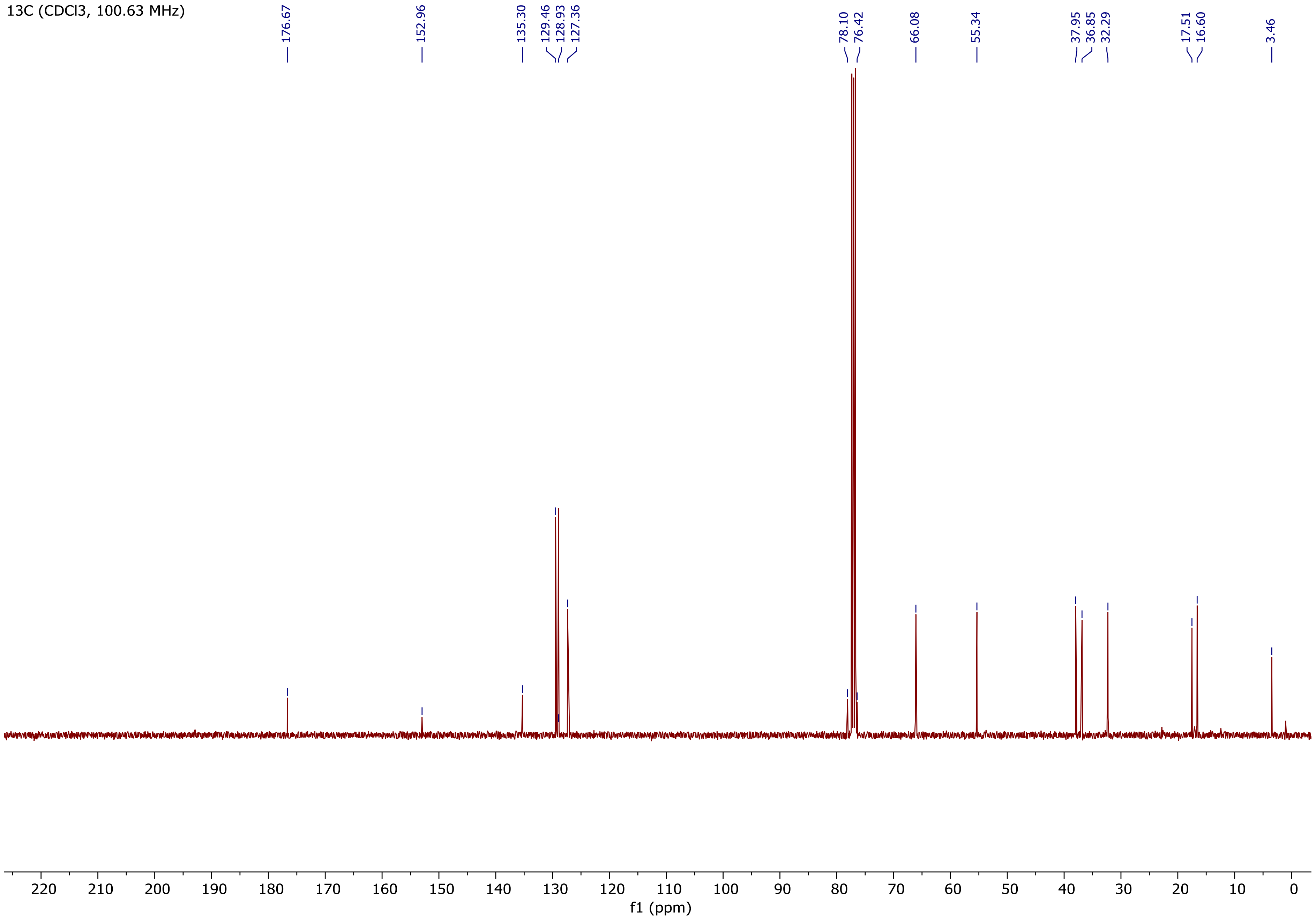

13C (CDCl3, 100.63 MHz)
176.67
152.96
135.30
129.46
128.93
127.36
78.10
76.42
66.08
55.34
37.95
36.85
32.29
17.51
16.60
3.46
220 210 200 190 180 170 160 150 140 130 120 110 100 90 80 70 60 50 40 30 20 10 0
f1 (ppm)

(2*R*,3*R*)-3-methyl-1-(4-tosyloxazol-5-yl)oct-6-yn-2-ol (7a)

(2*R*,3*R*)-3-methyl-1-(4-tosyloxazol-5-yl)oct-6-yn-2-ol **7a** was prepared by a five-step sequence starting from (*R*)-4-benzyl-3-((*R*)-2-methylhept-5-ynoyl)oxazolidin-2-one **11a** .

Step 1 (Hydrolysis):

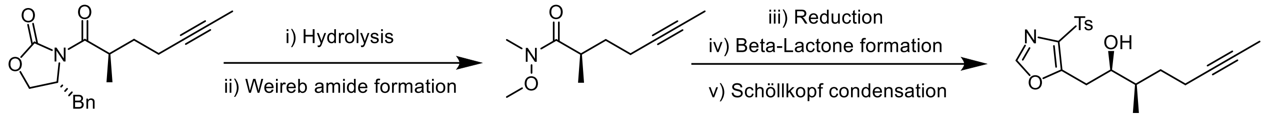

11a **13a** **7a**

(*R*)-2-methylhept-5-ynoic acid (12a)

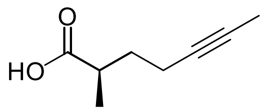

A one-neck round-bottomed flask equipped with a stir bar, was charged with a solution of (*R*)-4-benzyl-3-((*R*)-2-methylhept-5-ynoyl)oxazolidin-2-one **11a** (3.6 g, 12.0 mmol) in THF-H_2O (50 mL, 1:1). The reaction mixture was cooled to 0 °C, before H_2O_2 (4.7 mL, 48.0 mmol, 4.0 equiv., 35 % w/w, aq.) and LiOH (574 mg, 24.0 mmol, 2.0 equiv.) were added. The resulting mixture was allowed to warm to rt and then stirred overnight. After that the THF was evaporated under reduced pressure and the aqueous fraction obtained was extracted with CH_2Cl_2 (2 x 50 mL). The aqueous solution was then acidified with dilute HCl (1 M ,aq) to pH ~1 and re-extracted with CH_2Cl_2 (4 x 50 mL). The combined organic extracts were dried over $MgSO_4$, filtered and then concentrated under reduced pressure. This gave the product as a colourless oil (1.6 g, 95 %). ^{1}H NMR (400 MHz, $CDCl_3$): δ = 2.72 – 2.58 (m, 1H), 2.21 (tp, *J* = 7.4, 2.4 Hz, 2H), 1.91 (dtd, *J* = 13.4, 7.4, 6.9 Hz, 1H), 1.77 (t, *J* = 2.4 Hz, 3H), 1.59 (dtd, *J* = 13.4, 7.4, 6.5 Hz, 1H), 1.21 (d, *J* = 7.4 Hz, 3H). ^{13}C NMR (101 MHz, $CDCl_3$): δ = 182.4, 78.3, 76.9, 38.5, 32.8, 17.10, 17.06, 3.9. $[\alpha]_D^{20}$= −51.8 (c = 1.05, $CHCl_3$); IR (film): $\tilde{\nu}$ = 2974, 2921, 2660, 1702, 1465, 1418, 1379, 1287, 1244, 1203, 1181, 1125, 1061, 939, 801, 638, 538, 467, 409 cm^{-1}; HRMS-CI (*m/z*): calc'd. for $C_8H_{13}O_2$ $[M+H]^+$, 141.0910; found, 141.0909.

The optical purity of was determined (93 %ee) by HPLC on a crude sample of the acid **12a** using a chiral stationary phase (Chiralcel OD-3, 3 µm, 150 mm, ∅ 4.6 mm). The racemate of acid **12a** was prepared via three step sequence consisting of initial alkylation of diethyl methyl malonate with 5-iodopent-2-yne **S2**, followed by Krapcho decarboxylation reaction and saponification.

Operator : Hi
Sample Name : YIE-YA-330-00
Vial # : 1
Injection Volume : 1 uL
Data File Name : YIE-YA-330-00-03.lcd
Method File Name : Yiannakas.lcm

1 µl YIE-YA-330-00 (in 0.5 ml 2-Propanol)
150 mm Chiralcel OD-3, 4.6 mm i.D., Säule 1
n-Heptan / 2-Propanol = 95:5
1.0 mL/min, 9.2 MPa, 298 K
UV, 220 nm

Säulenauswahlventil
<<Oven>>
Valve L : 2
Valve R : 0

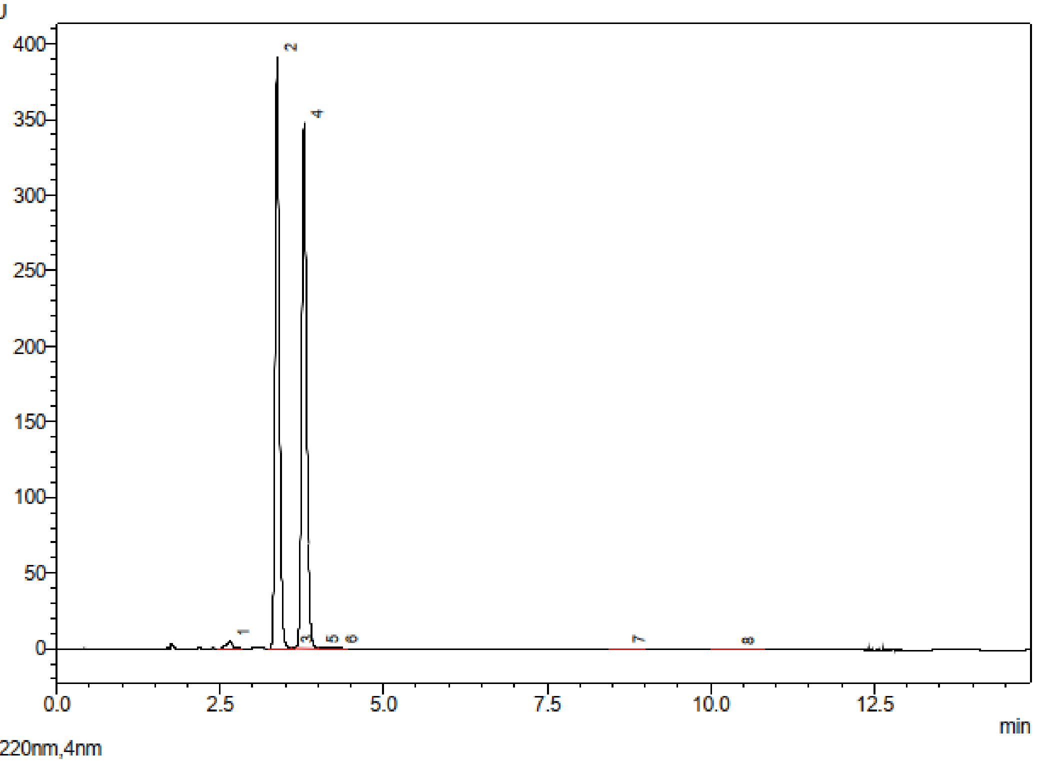

1 220nm,4nm

PDA Ch1 220nm

Peak #	Ret. Time	Area %	Name
1	2.65	0.96	
2	3.37	48.86	1st Enantiomer
3	3.62	0.09	
4	3.78	49.27	2nd Enantiomer
5	4.02	0.09	
6	4.30	0.23	
7	8.70	0.24	
8	10.37	0.25	
Total		100.00	

Operator : Be
Sample Name : YIE-YA-324-02
Vial # : 2
Injection Volume : 3 uL
Data File Name : YIE-YA-324-02-01.lcd
Method File Name : Yiannakas.lcm

Data Acquired: 28.08.2020 09:20:49

3.0 µL YIE-YA-324-02
ca. 4.0 mg in 500 µL n-Heptan
150 mm Chiralcel OD-3, 4.6 mm i.D., Säule 2
n-Heptan / i-Propanol / TFA = 98:2:0.1
1.0 mL/min, 9.0 MPa, 298 K
UV, 220 nm

Säulenauswahlventil
<<Oven>>
Valve L : 2
Valve R : 0

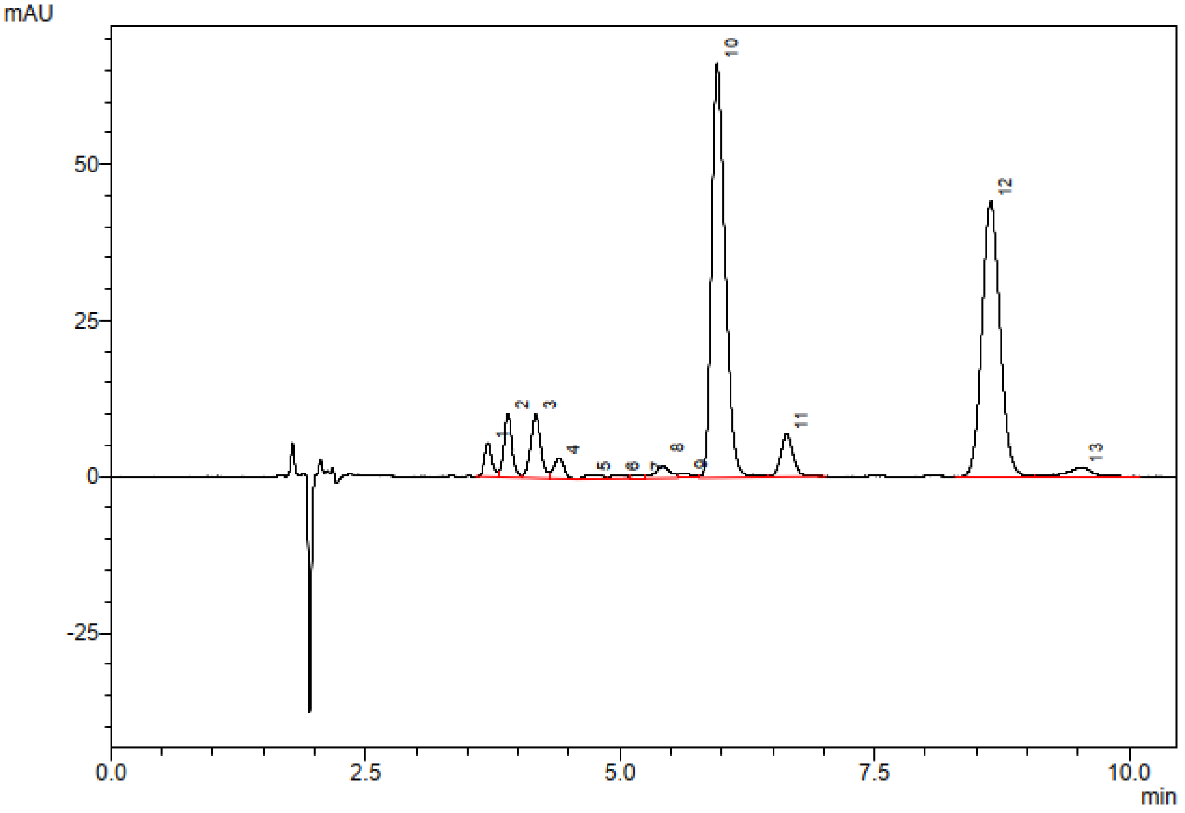

1 220nm,4nm

PDA Ch1 220nm

Peak #	Ret. Time	Area %	Name
1	3.70	1.90	
2	3.90	3.90	
3	4.17	4.79	
4	4.40	1.57	
5	4.70	0.53	
6	4.98	0.41	
7	5.23	0.22	
8	5.42	1.41	1.Enantiomer
9	5.65	0.44	
10	5.96	41.84	2.Enantiomer
11	6.64	3.99	
12	8.64	37.08	
13	9.54	1.91	
Total		100.00	

ee = 93 %

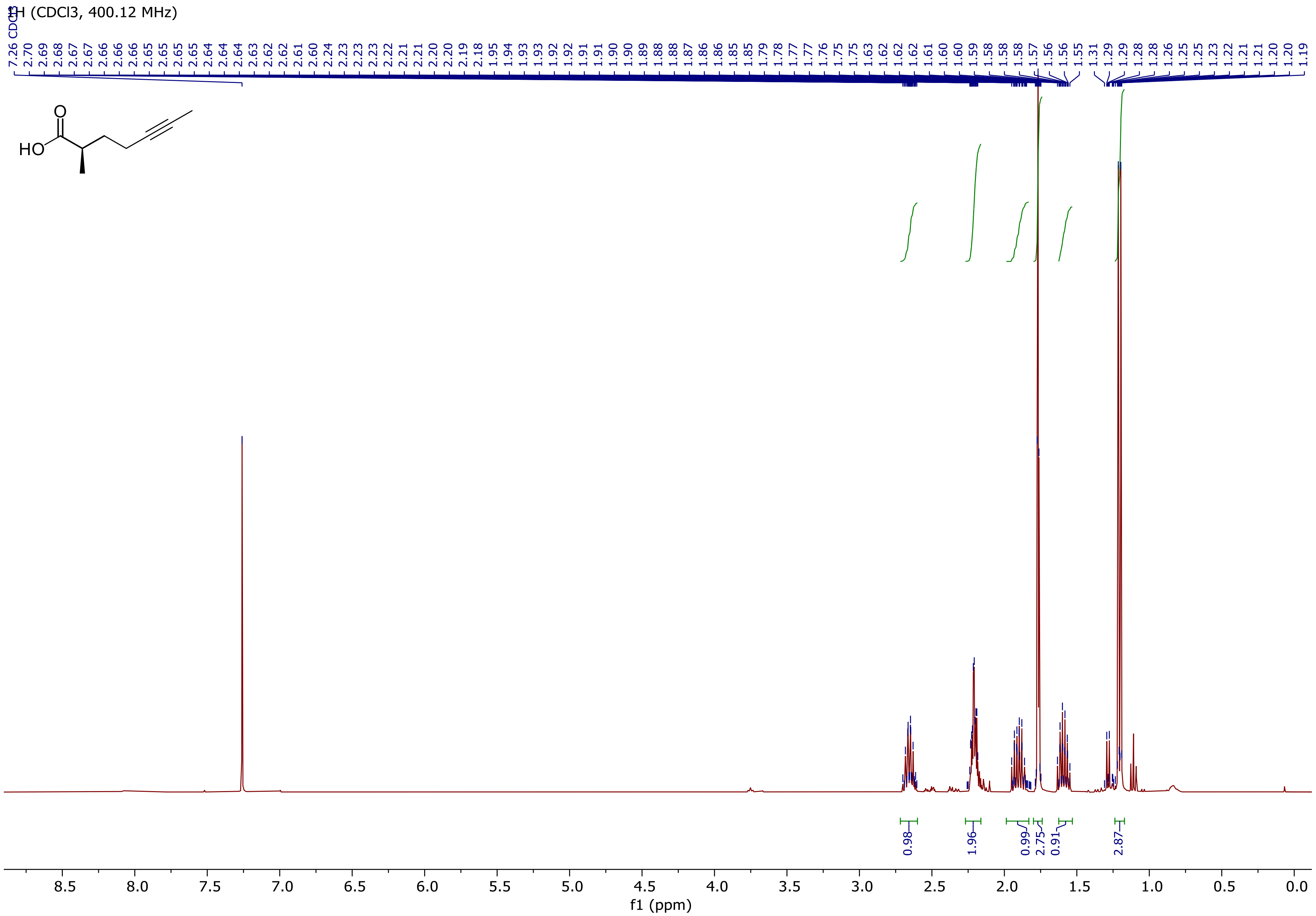
1H (CDCl3, 400.12 MHz)
HO
O
f1 (ppm)
0.98
1.96
0.99
2.75
0.91
2.87

13C (CDCl3, 100.62 MHz)

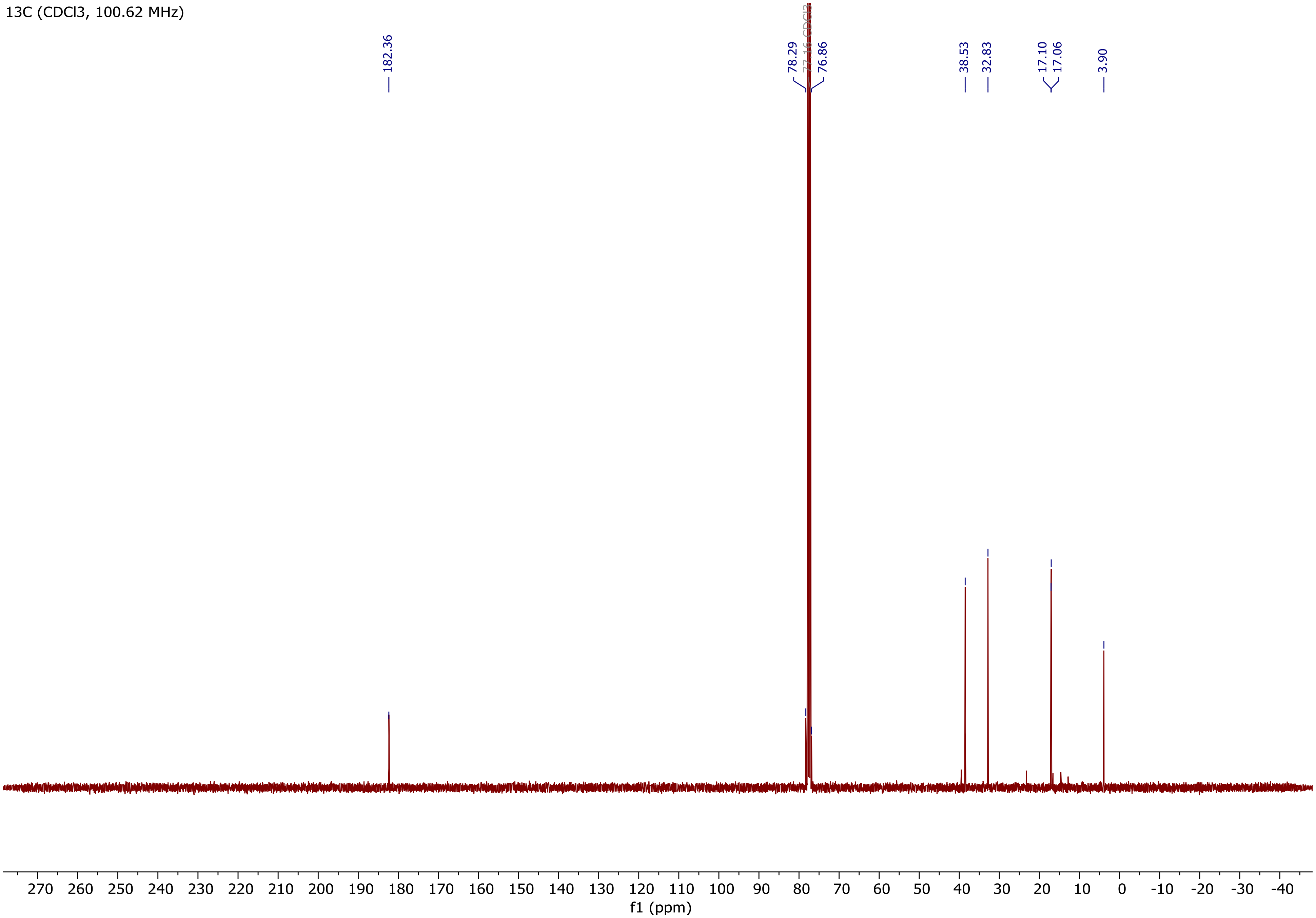

Step 2 (Weinreb amide formation):

(R)-N-nethoxy-N,2-diemthylhept-5-ynamide (13a):

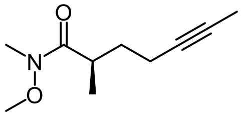

A two-neck round round-bottomed flask equipped with a magnetic stir bar, a glass stopper and a gas inlet connected to an argon-vacuum manifold, was charged with solution of acid **12a** (1.6 g, 11.4 mmol) in CH_2Cl_2 (56 mL). Upon cooling to 0 °C, 1,1-carbonyldiimidazole (2.4 g, 14.4 mmol, 1.2 equiv.) was added and the resulting mixture was stirred at this temperature for 0.5 h. After that *N,O*-dimethylhydroxylamine-hydrochloride (2.9 g, 30.0 mmol, 2.5 equiv.) was added and the reaction mixture was allowed to stir at rt overnight. The slurry obtained was then filtered and the filtrate was washed with dilute HCl (30 mL, 1 M, aq.). The organic phase was then dried over $MgSO_4$, filtered, and then concentrated under reduced pressure. The residue obtained (2.0 g, a mixture of the desired amide **13a** and residual Weinreb amine) was then progressed to the next step without any further purification, due concerns about potential racemization. ^{1}H NMR (400 MHz, $CDCl_3$): δ = 3.72 (s, 3H), 3.67 (s, 3H), 3.19 (s, 3H), 3.08 (s, 3H), 2.22 – 2.08 (m, 2H), 1.89 (ddt, *J* = 13.4, 8.4, 6.9 Hz, 1H), 1.76 (t, *J* = 2.6 Hz, 3H), 1.57 – 1.44 (m, 1H), 1.12 (d, *J* = 6.9 Hz, 3H). ^{13}C NMR (101 MHz, $CDCl_3$) δ 79.2, 76.3, 61.8, 61.0, 36.6, 34.5, 33.0, 17.7, 17.2, 3.9. HRMS-EI (*m/z*): calc'd. for $C_{10}H_{17}NO_2$ $[M]^{+\cdot}$, 183.1253; found, 183.1254.

The optical purity of was determined (93.7 %ee) by HPLC on a crude sample of the amide **13a** using a chiral stationary phase (Chiralcel OD-3, 3 µm, 150 mm, ∅ 4.6 mm). Racemate of Weinreb amide **13a** was prepared by amidation of the racemate of acid **12a**.

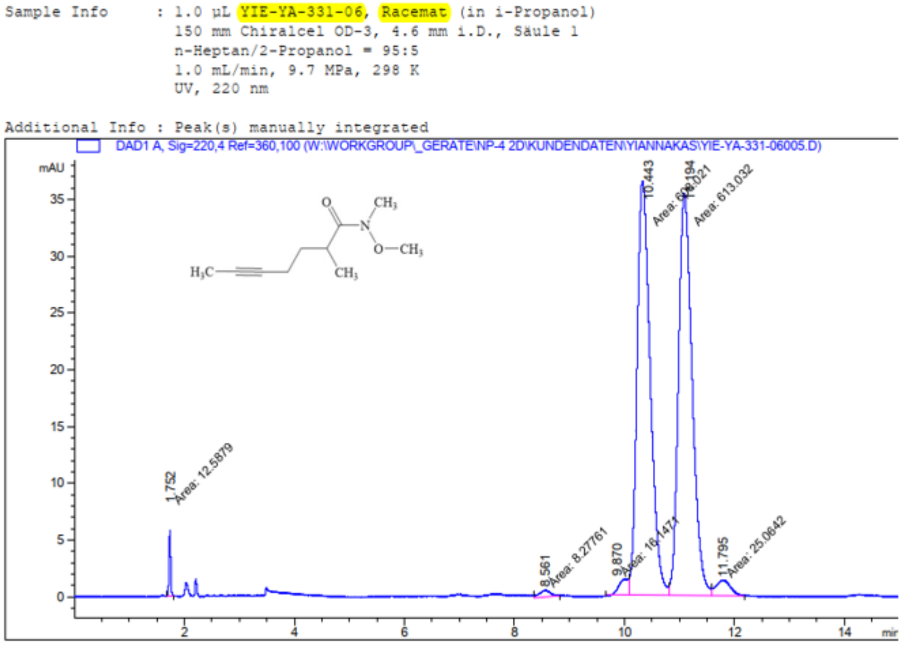

Area Percent Report

Sorted By : Signal
Multiplier : 1.0000
Dilution : 1.0000
Do not use Multiplier & Dilution Factor with ISTDs

Signal 1: DAD1 A, Sig=220,4 Ref=360,100

Peak #	RetTime [min]	Type	Width [min]	Area [mAU*s]	Height [mAU]	Area %
1	1.752	MM	0.0267	12.58789	7.86406	0.9810
2	8.561	MM	0.2325	8.27761	5.93254e-1	0.6451
3	9.870	MF	0.1975	16.14715	1.36295	1.2584

Peak #	RetTime [min]	Type	Width [min]	Area [mAU*s]	Height [mAU]	Area %	
4	10.443	MF	0.2776	608.02130	36.50487	47.3858	1st enantiomer
5	11.194	MF	0.2853	613.03192	35.81454	47.7763	2nd enantiomer
6	11.795	FM	0.3084	25.06424	1.35466	1.9534	

Totals : 1283.13011 83.49433

Sample Info : 1.0 µL YIE-YA-324-03, chirale Probe (in i-Propanol)
150 mm Chiralcel OD-3, 4.6 mm i.D., Säule 1
n-Heptan/2-Propanol = 95:5
1.0 mL/min, 9.7 MPa, 298 K
UV, 220 nm

Additional Info : Peak(s) manually integrated

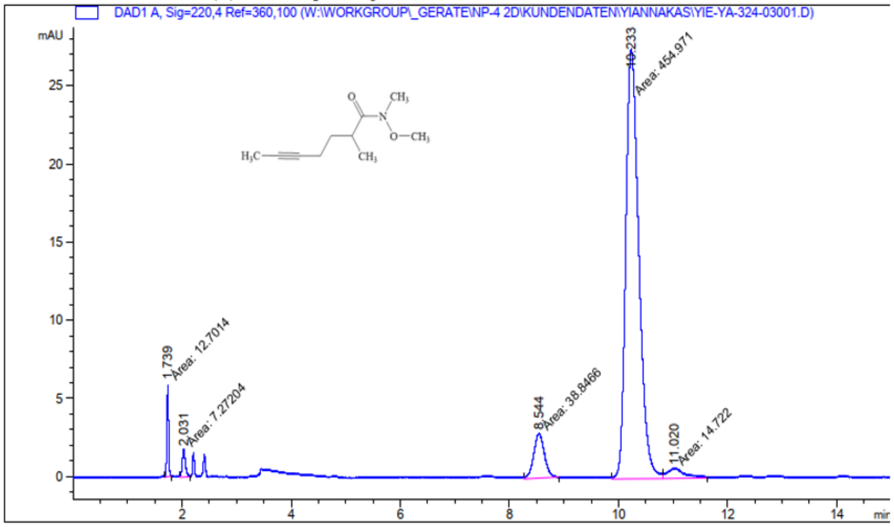

===
Area Percent Report
===

Sorted By : Signal
Multiplier : 1.0000
Dilution : 1.0000
Do not use Multiplier & Dilution Factor with ISTDs

Signal 1: DAD1 A, Sig=220,4 Ref=360,100

Peak #	RetTime [min]	Type	Width [min]	Area [mAU*s]	Height [mAU]	Area %
1	1.739	MM	0.0353	12.70141	5.99324	2.4032
2	2.031	MM	0.0681	7.27204	1.77996	1.3759
3	8.544	MM	0.2264	38.84655	2.85997	7.3502

Peak #	RetTime [min]	Type	Width [min]	Area [mAU*s]	Height [mAU]	Area %	
4	10.233	MF	0.2754	454.97144	27.52904	86.0851	1st enantiomer
5	11.020	FM	0.3905	14.72203	6.28361e-1	2.7856	2nd enantiomer

ee = 93.7 %

Totals : 528.51346 38.79058

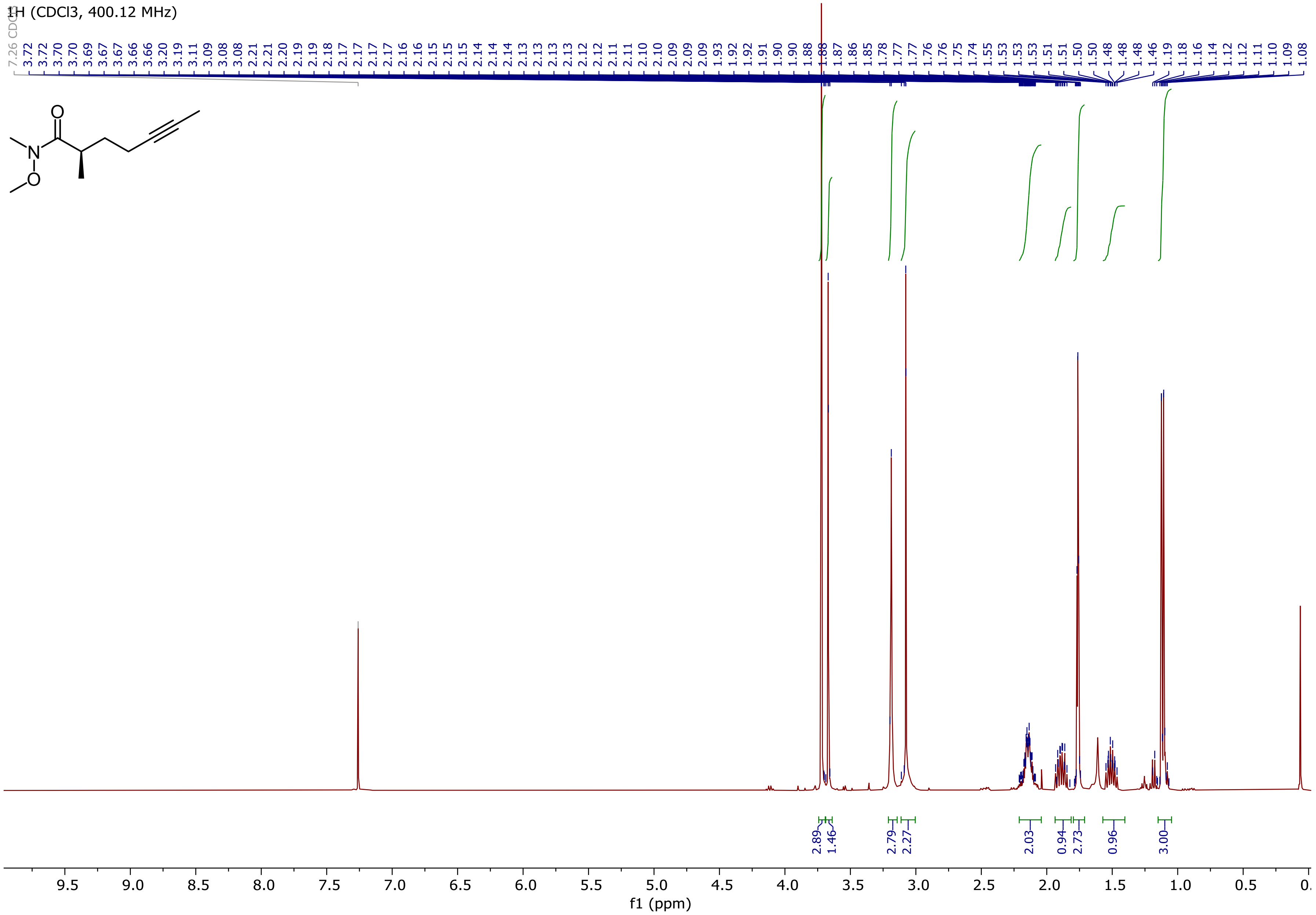
1H (CDCl3, 400.12 MHz)
7.26 CDCl3
3.72 3.72 3.70 3.69 3.67 3.67 3.66 3.66 3.20 3.19 3.11 3.09 3.08 3.08 2.21 2.21 2.20 2.19 2.19 2.18 2.17 2.17 2.17 2.16 2.16 2.15 2.15 2.14 2.14 2.13 2.13 2.13 2.13 2.12 2.12 2.11 2.11 2.10 2.10 2.09 2.09 2.09 1.93 1.92 1.92 1.91 1.90 1.88 1.88 1.87 1.86 1.85 1.78 1.77 1.77 1.76 1.75 1.74 1.55 1.53 1.53 1.53 1.51 1.51 1.50 1.50 1.48 1.48 1.48 1.46 1.19 1.18 1.16 1.14 1.12 1.12 1.11 1.10 1.09 1.08
O
N
O
2.89
1.46
2.79
2.27
2.03
0.94
2.73
0.96
3.00
9.5 9.0 8.5 8.0 7.5 7.0 6.5 6.0 5.5 5.0 4.5 4.0 3.5 3.0 2.5 2.0 1.5 1.0 0.5
f1 (ppm)

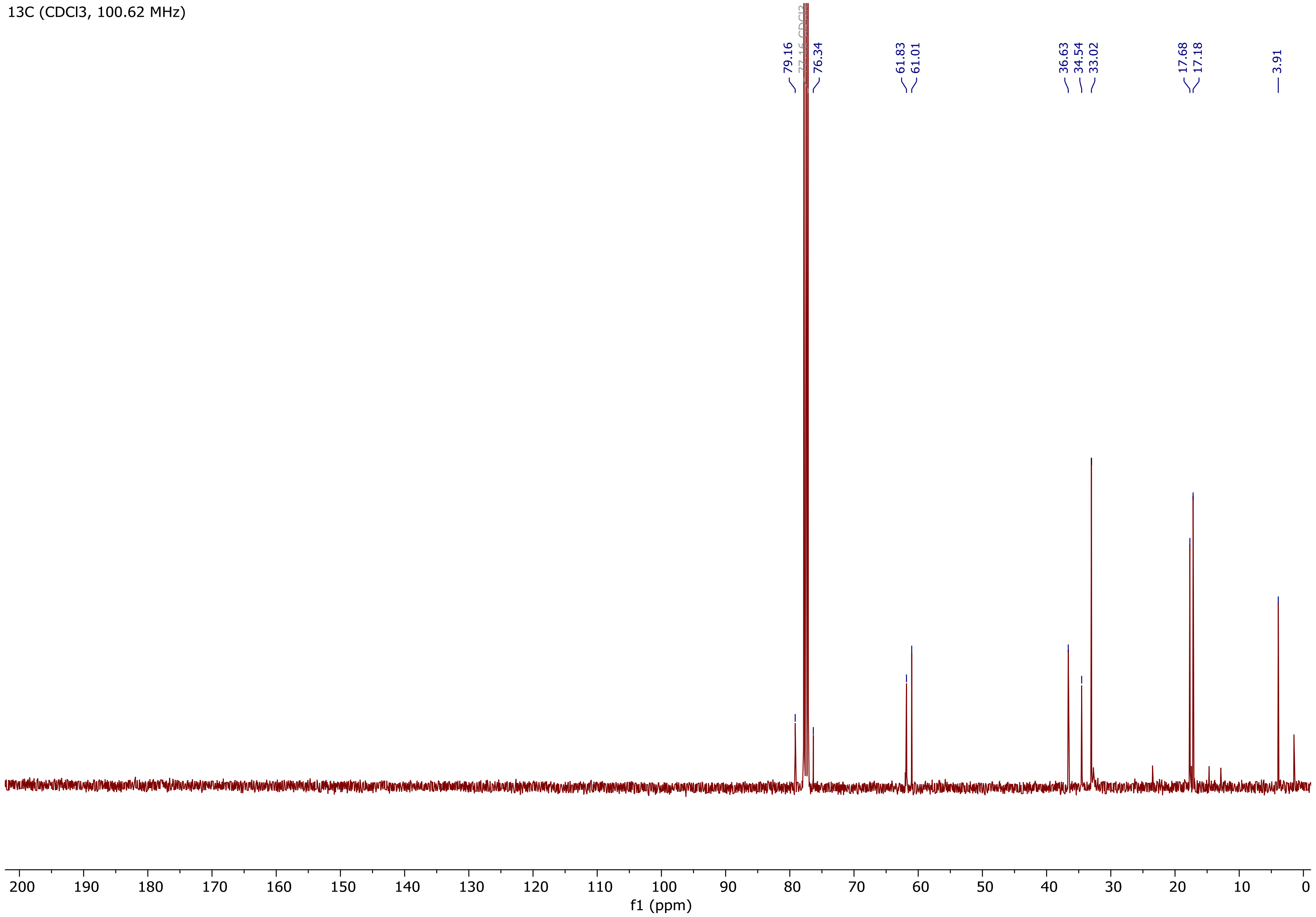

13C (CDCl3, 100.62 MHz)
79.16
77.16 CDCl3
76.34
61.83
61.01
36.63
34.54
33.02
17.68
17.18
3.91
200
190
180
170
160
150
140
130
120
110
100
90
80
70
60
50
40
30
20
10
0
f1 (ppm)

Step 3 & 4 (Weinreb Amide reduction & Cyclocondesation):

A three-neck jacketed vessel equipped with a stir bar, a gas inlet connected to an argon-vacuum manifold, a ground-glass joint thermometer adapter connected to low temperature thermometer and a rubber septum was charged with a solution of the crude amide **13a** (2.0 g, 12.0 mmol) in CH_2Cl_2 (56 mL). The reaction mixture was cooled to –78 °C and DIBAL-H (15.8 mL, 15.8 mmol, 1.5 equiv., 1.0 M in *n*-hexanes) was added dropwise. The resulting mixture stirred for 1 h at this temperature, before it was quenched with Rochelle salt (50 mL, sat.aq.). The slurry immediately formed was allowed to warm to rt. The biphasic mixture obtained was then separated and the aqueous layer was extracted with CH_2Cl_2 (3 x 50 mL). The combined organic extracts were dried over $MgSO_4$, filtered, and then concentrated under reduced pressure (300m mbar) at rt. The crude aldehyde **9a** was engaged to the next without any purification. A three-neck jacketed vessel equipped with a stir bar, a gas inlet connected to an argon-vacuum manifold, a ground-glass joint thermometer adapter connected to low temperature thermometer and a rubber septum was charged with a solution of lithium perchlorate (1.1 g, 10.8 mmol, 0.9 equiv.) in Et_2O (17 mL) and a solution of TMS*q* (429 mg, 1.1 mmol, 10 mol%) in CH_2Cl_2 (34 mL). The mixture was stirred for 0.5 h at rt and then cooled to –78 °C. DIPEA (4.7 mL, 27.0 mmol, 2.25 equiv.) was added, followed by a solution of the crude aldehyde **9a** in CH_2Cl_2-Et_2O (51 mL, 2:1). A solution of AcCl (1.14 mL, 16.0 mmol, 1.3 equiv.) in CH_2Cl_2 (9 mL) was added via syringe pump over 5 h and the resulting mixture was stirred at –78 °C for 24 h. The reaction mixture was diluted with Et_2O (50 mL) and then allowed to warm to rt. The yellow suspension obtained was then filtered through a silica plug eluting with Et_2O (100 mL) and the filtrate obtained was concentrated under reduced pressure (300 mbar) at rt. This afforded the crude beta-lactone **7a** as a pale yellow oil (96:4 dr), which was subjected to the next step without any purification.

Step 5 (Schöllkopf Condensation):

A three-neck jacketed vessel equipped with a stir bar, a gas inlet connected to an argon-vacuum manifold, a ground-glass joint thermometer adapter connected to low temperature thermometer and a rubber septum, was charged with a solution of TosMIC (2.2 g, 10.9 mmol, 1.0 equiv.) in THF (120 mL). The solution was cooled to –78 °C and ^{n}BuLi (7.5 mL, 12.0 mmol, 1.0 equiv., 1.6 M in *n*-hexane) was added dropwise. The pale yellow solution obtained was stirred at –78 °C for 0.5 h, before a solution of the crude beta-lactone **8a** in THF (20 mL) was added. The resulting mixture was stirred at –78 °C for an hour and then warmed to rt. The reaction was then quenched with HCl (20 mL, 1.0 M, aq.) and extracted with EtOAc (3 x 100 mL). The combined organic extracts were dried over $MgSO_4$, filtered and then concentrated under reduced pressure. The dark brown oily residue obtianed was purified by flash column chromatography (SiO_2, 100 g, 55 mm ø, 0-100% EtOAc/iso-Hexanes, ca. 20 mL). This gave the product as an orange viscous oil (1.5 g, 35 % yield over 5 steps, 96:4 dr). ^{1}H NMR (400 MHz, $CDCl_3$): δ = 7.90 – 7.83 (m, 2H), 7.69 (s, 1H), 7.32 – 7.24 (m, 2H), 3.88 (ddt, *J* = 9.6, 6.8, 3.6 Hz, 1H), 3.27 (dd, *J* = 14.8, 9.6 Hz, 1H), 3.13 (dd, *J* = 14.8, 3.6 Hz, 1H), 2.36 (s, 3H), 2.26 – 2.03 (m, 2H), 1.94 (d, *J* = 6.6 Hz, 1H), 1.79 – 1.59 (m, 4H), 1.38 (dddd, *J* = 13.3, 8.4, 7.1, 6.8 Hz, 1H), 0.94 (d, *J* = 6.8 Hz, 3H); ^{13}C NMR (101 MHz, $CDCl_3$): δ = 155.4, 150.2, 145.5, 137.4, 137.2, 130.4, 128.6, 79.2, 76.6, 73.7, 38.3, 32.6, 31.5, 22.2, 16.9, 13.7, 3.9; $[a]_D^{20}$= + 21.0 (c = 2.41, $CHCl_3$); IR (film): $\tilde{\nu}$ = 3527, 3128, 2921, 1595, 1517, 1494, 1449, 1402, 1382, 1342, 1305, 1245, 1217, 1147, 1085, 1059, 1017, 854, 815, 709, 697, 663, 601, 540, 502 cm^{-1}; HRMS-ESI+ (*m/z*): calc'd. for $C_{19}H_{23}NO_4SNa$ $[M+Na]^+$, 384.1240; found, 384.1241.

1H (CDCl3, 400.12 MHz)

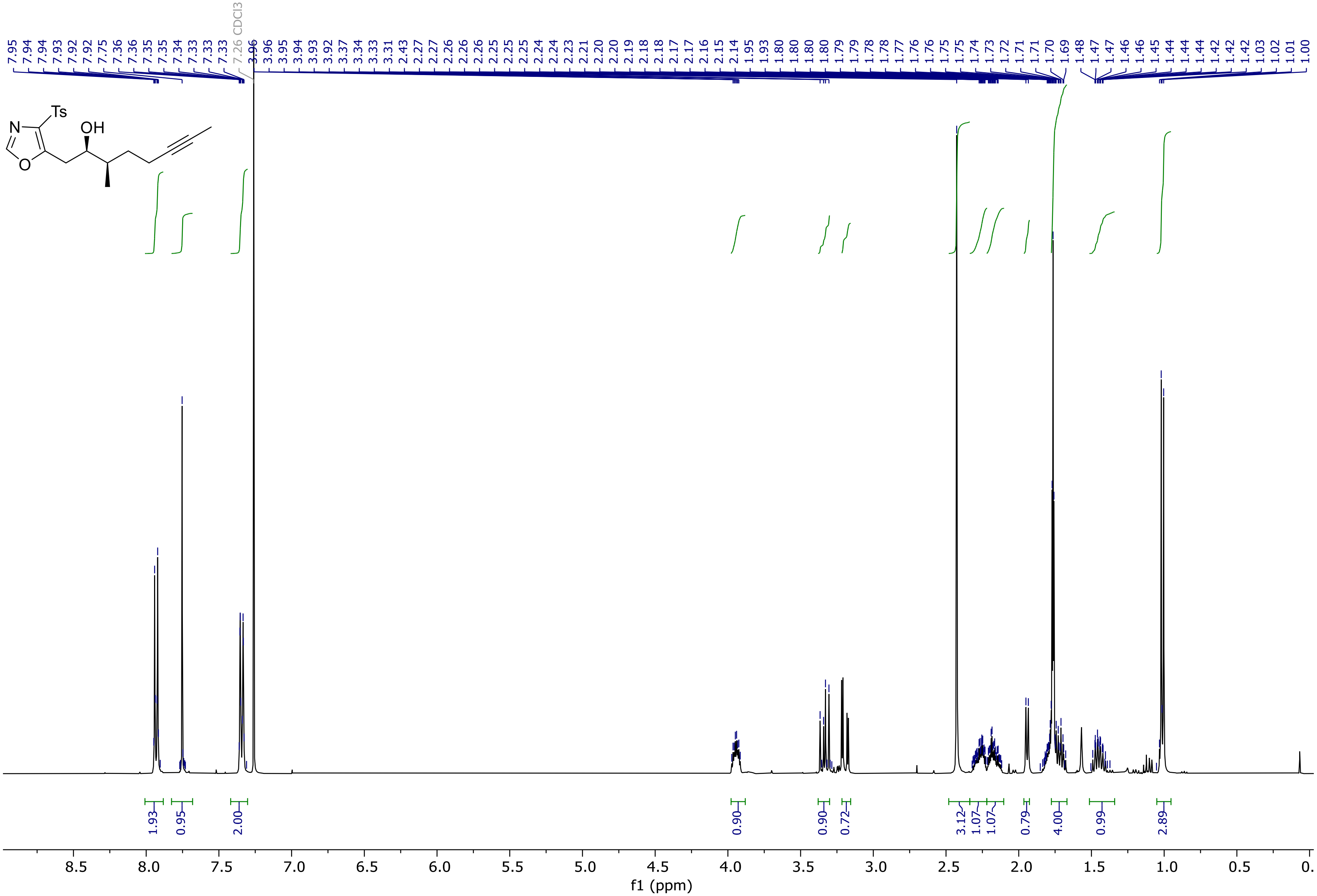

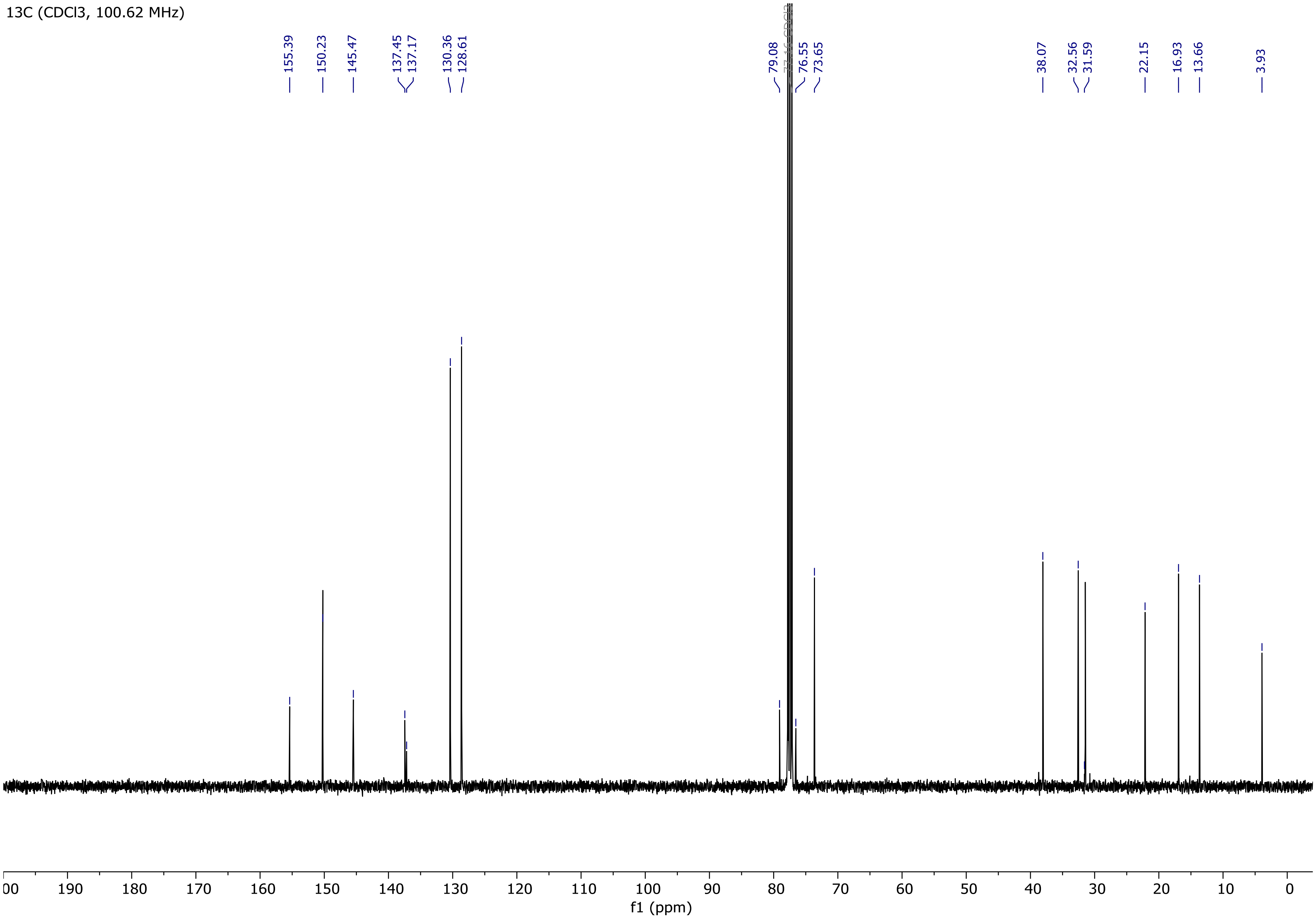
13C (CDCl3, 100.62 MHz)
155.39
150.23
145.47
137.45
137.17
130.36
128.61
79.08
76.55
73.65
38.07
32.56
31.59
22.15
16.93
13.66
3.93
f1 (ppm)

(*R*)-*N*-((1*S*,2*S*)-1-hydroxy-1-phenylpropan-2-yl)-*N*,2-dimethylhept-5-ynamide (18a)

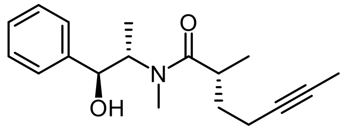

A three-neck jacketed vessel equipped with a stir bar, a gas inlet connected to an argon-vacuum manifold, a ground-glass joint thermometer adapter connected to low temperature thermometer and a rubber septum, was charged with a solution of lithium chloride (866 mg, 20.4 mmol, 4.3 equiv.) and diisopropylamine (2.9 mL, 20.4 mmol, 4.3 equiv.) in THF (77 mL). Upon cooling of the mixture to 0 °C, ^{n}BuLi (11.9 mL, 12.0 mmol, 4.3 equiv., 1.6 M in *n*-hexanes) was added dropwise and the mixture was stirred at this temperature for 1 h. After that the reaction mixture was cooled to - 78 °C and a solution of *N*-((1*S*,2*S*)-1-hydroxy-1-phenylpropan-2-yl)-*N*-methylpropionamide **17** (2.2 g, 10 mmol, 2.1 equiv.) in THF (33 mL) was added dropwise. The mixture was then stirred for 1 h at - 78 °C, before it was warmed to 0 °C. 5-Iodopent-2-yne (1.0 g, 4.7 mmol) was added and the resulting mixture was then stirred for 2 h at 0 °C. The reaction mixture was then quenched with NH_4Cl (100 mL, sat. aq.) and then extracted with EtOAc (3 x 100 mL). The combined organic extracts were dried over $MgSO_4$, filtered and then concentrated under reduced pressure. The orange oily residue obtained was then purified by flash column chromatography (SiO_2, 100 g, 55 mm ø, 0-100% EtOAc/iso-Hexanes, ca. 20 mL). This afforded the product as a thick pale yellow syrup (1.4 g, 95 %). ^{1}H NMR (600 MHz, DMSO, mixture of rotamers): δ = 7.38 – 7.35 (m, 3H, major), 7.33 – 7.30 (m, 3H, major), 7.29 – 7.27 (m, 3H, minor), 7.26 – 7.23 (m, 2H, major), 7.21 – 7.18 (m, 1H, minor), 5.41 (d, *J* = 3.7 Hz, 1H, major), 5.28 (d, *J* = 4.6 Hz, 1H, minor), 4.67 (s, 1H, minor), 4.54 (dd, *J* = 7.7, 4.5 Hz, 1H, minor), 4.51 (dd, *J* = 8.1, 3.7 Hz, 1H, major), 3.99 – 3.93 (m, 1H, major), 2.85 (s, 3H, major), 2.84 – 2.79 (m, 1H, minor), 2.77 – 2.73 (m, 1H, major), 2.72 (s, 3H, minor), 1.96 (dddt, *J* = 14.7, 9.6, 7.3, 2.6 Hz, 4H, major, minor), 1.84 – 1.75 (m, 1H, major), 1.66 – 1.58 (m, 1H, minor), 1.35 – 1.25 (m, 2H, major, minor), 0.91 (d, *J* = 5.7 Hz, 3H, major), 0.90 (s, 3H, major), 0.86 (d, *J* = 6.9 Hz, 3H, minor), 0.80 (d, *J* = 6.8 Hz, 3H, minor); ^{13}C NMR (151 MHz, DMSO, mixture of romatmers): δ = 175.3, 174.9, 143.4, 143.3, 127.9, 127.5, 127.1, 126.7, 126.7, 126.4, 79.1, 78.7, 75.7, 75.4, 73.8, 65.2, 56.7, 53.4, 33.9, 33.7, 33.6, 32.8, 32.8, 29.9, 26.7, 17.2, 16.8, 15.9, 15.2, 14.0, 13.9, 2.9; $[\alpha]_D^{20}$= + 23.8 (c = 1.00, EtOH); mp: 67-69 °C; (IR (film): ṽ = 3364, 2974, 2917, 2849, 1614, 1517, 1483, 1450, 1408, 1374, 1350, 1302, 1245, 1233, 1198, 1140,1108, 1082, 1051, 1026, 1001, 924, 900, 860, 762, 702, 610, 540 cm^{-1}; HRMS-ESI+ (*m/z*): calc'd. for $C_{18}H_{25}NO_2Na$ $[M+Na]^+$, 310.1777; found, 310.1780. The optical purity of diastereomerically pure amide **18a** was determined (80.7: 1 dr) by HPLC on a crude sample of the amide **18a** using a chiral stationary phase (Eclipse Plus C_{18}, 50 mm, ∅ 4.6 mm).

Amide **18a** was epimerized by stirring with trifluoroacetic acid (10 equiv.) in THF at reflux for 1 h (effecting N → O acyl transfer as well as α-epimerization), followed by neutralization with sat. $NaHCO_3$ solution at 23 °C for 24 h (causing O → N acyl transfer). The optical purity of diastereomerically pure amide **18a** was determined (80.7: 1 dr) by HPLC on a crude sample of the amide **18a** using a chiral stationary phase (Eclipse Plus C_{18}, 50 mm, ∅ 4.6 mm).

Data file:	C:\ChemStationData\Kundendaten LC\Yiannakas\YIE-YA-288-01007.D
Sample name:	YIE-YA-288-01
Description:	1 µl YIE-YA-288-01 (in 1 ml Acetonitril)<br>Acetonitril/Wasser - Gradient:<br>30 % CH3CN - 5' -50 % CH3CN<br>1.0 mL / min, 17.7 MPa, 308 K<br>UV, 220 nm

Instrument: RR-Prime LC ohne ELSD

Column: Eclipse Plus C18 length 50.00 i.D. 4.60

Injection date:	8/11/2020 3:36:04 PM	Location:	P1-A-01
Acq. method:	Yiannakas.M	Injection volume:	1.000
Analysis method:	Yiannakas.M	Acq. operator:	SYSTEM
Last changed:	8/12/2020 11:17:29 AM		

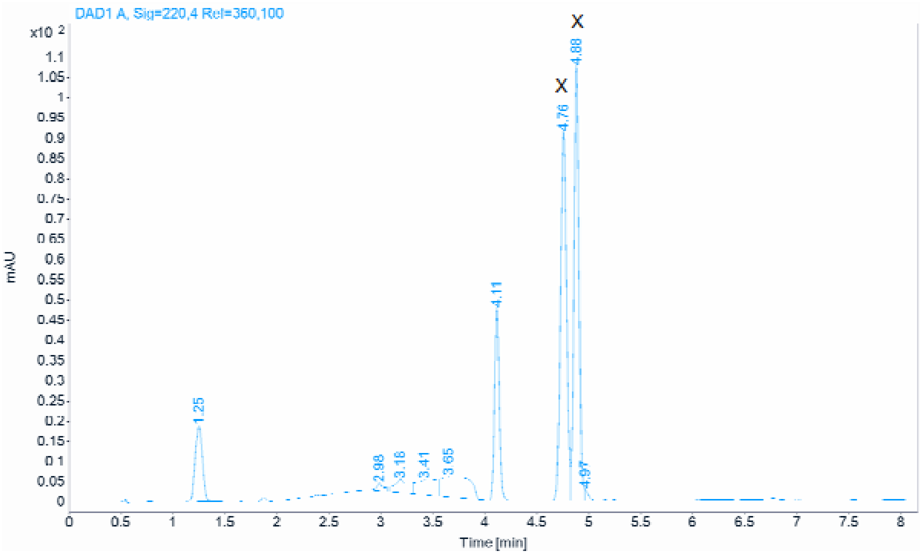

Signal: DAD1 A, Sig=220,4 Ref=360,100

RT [min]	Area	Area%	Name
1.25	93.79	7.98	
2.98	10.12	0.86	
3.18	31.95	2.72	
3.41	54.18	4.61	
3.65	97.37	8.28	
4.11	153.90	13.09	
4.76	372.10	31.66	1. Diastereomer
4.88	356.92	30.36	2. Diastereomer
4.97	5.12	0.44	
Sum	1175.4521		

**Data file:**	C:\ChemStationData\Kundendaten LC\Yiannakas\YIE-YA-288-02005.D
**Sample name:**	YIE-YA-288-02
**Description:**	1 µl YIE-YA-288-02 (in 1 ml Acetonitril)<br>Acetonitril/Wasser - Gradient:<br>30 % CH3CN - 5' -50 % CH3CN<br>1.0 mL / min, 16.0 MPa, 308 K<br>UV, 220 nm

Instrument: RR-Prime LC ohne ELSD

Column Eclipse Plus C18 length 50.00 i.D. 4.60

Injection date: 8/11/2020 3:50:29 PM
Acq. method: Yiannakas.M
Analysis method: Yiannakas.M
Last changed: 8/12/2020 11:17:29 AM

Location: P1-A-02
Injection volume: 1.000
Acq. operator: SYSTEM

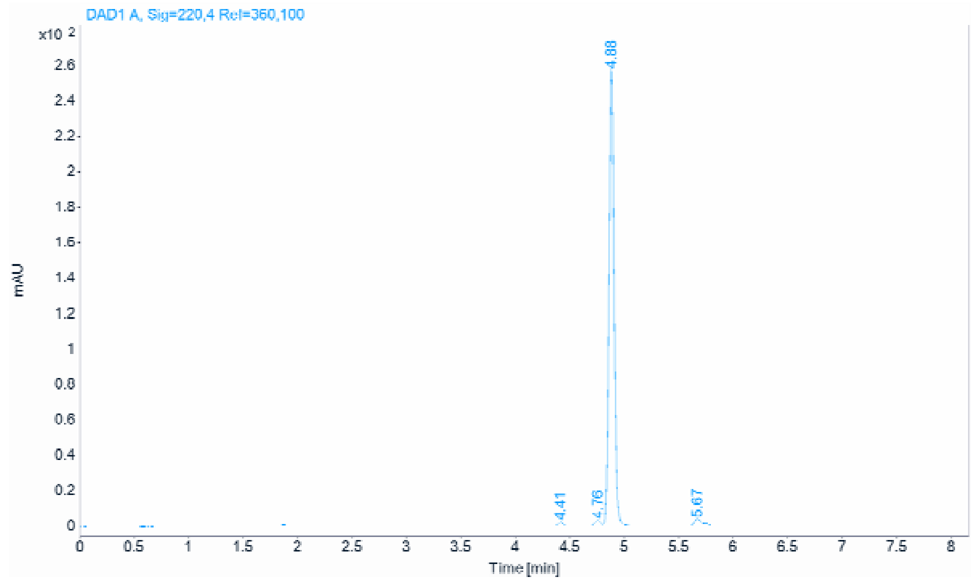

Signal: DAD1 A, Sig=220,4 Ref=360,100

RT [min]	Area	Area%	Name
4.41	6.64	0.75	
4.76	10.48	1.19	1. Diastereomer
4.88	844.45	95.96	2. Diastereomer
5.67	18.46	2.10	
Sum	880.0319		

dr = 1:80.7

1H (DMSO, 600.20 MHz)

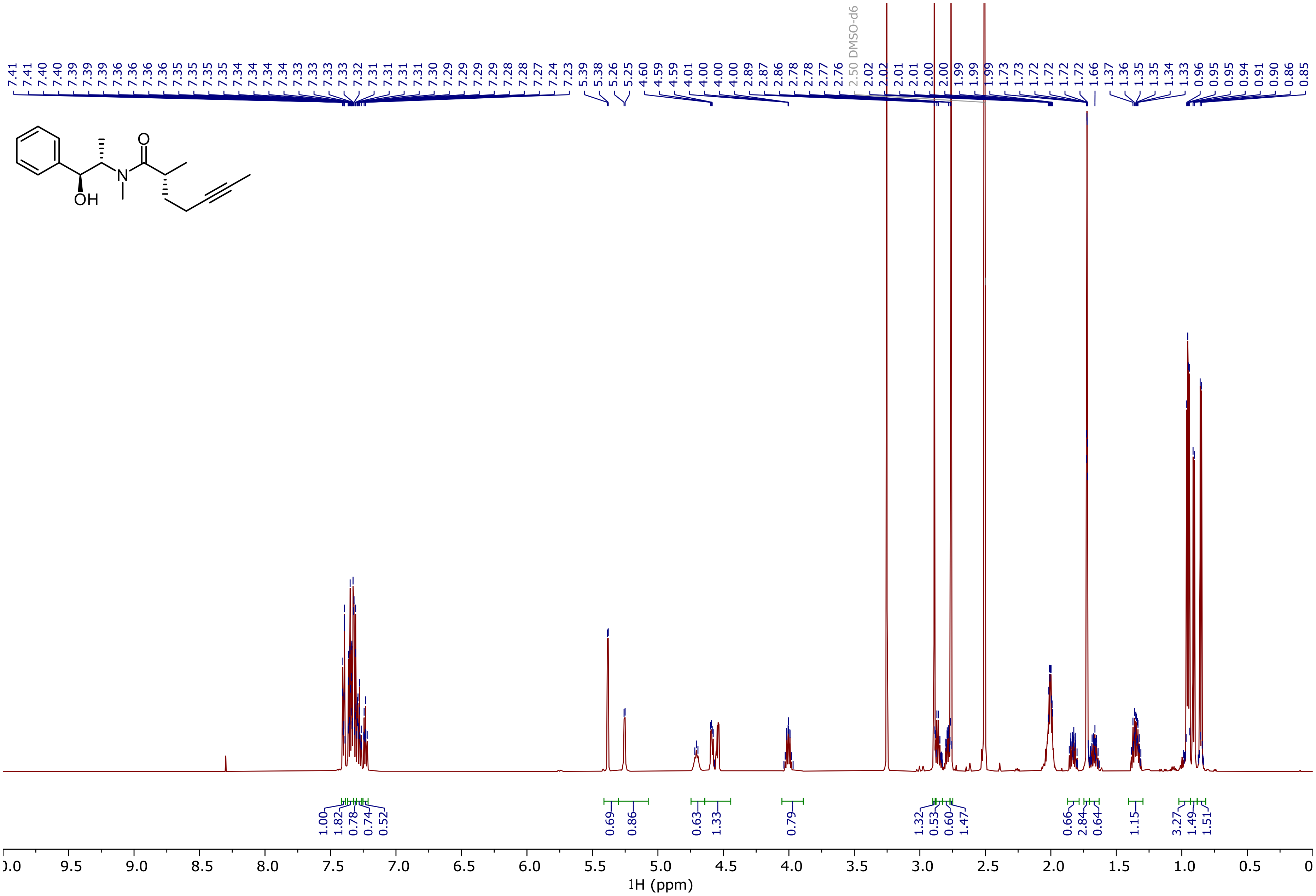

13C (DMSO, 150.94 MHz)

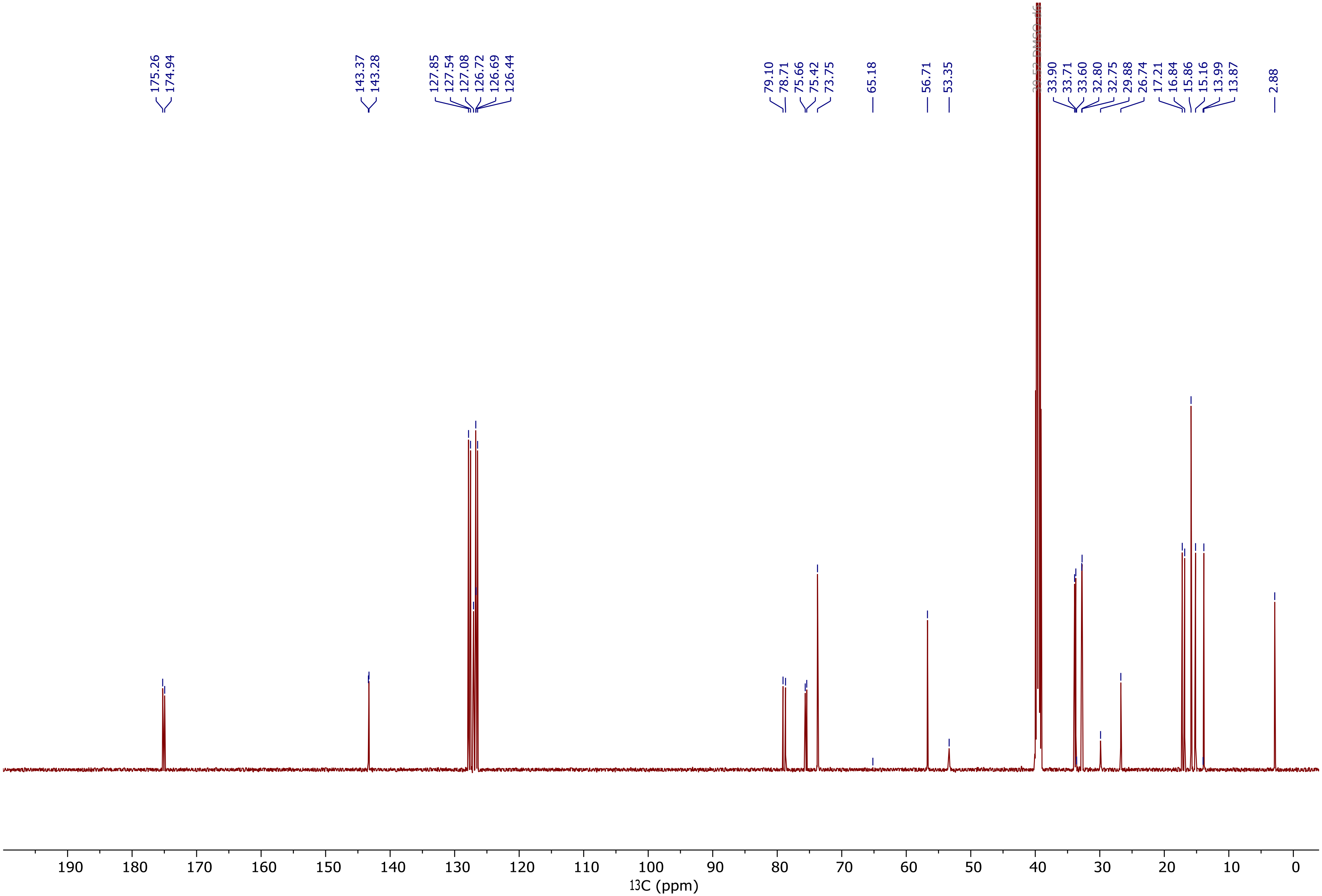

(2*R*,3*R*)-3-methyl-1-(4-tosyloxazol-5-yl)oct-6-yn-2-ol **7a** was prepared by a three-step sequence starting from (*R*)-N-((1*S*,2*S*)-1-hydroxy-1-phenylpropan-2-yl)-*N*,2-dimethylhept-5-ynamide **18a**:

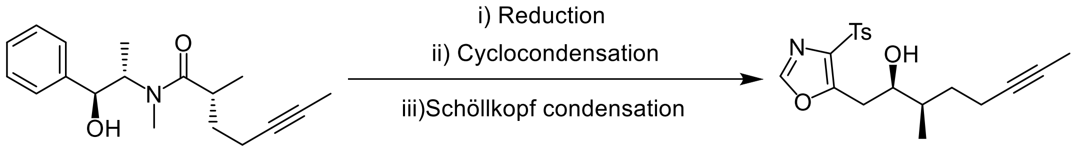

18a **7a**

A three-neck jacketed vessel equipped with a stir bar, a gas inlet connected to an argon-vacuum manifold, a ground-glass joint thermometer adapter connected to low temperature thermometer and a rubber septum was charged with $LiAlH_4$ (708 mg, 18.7 mmol, 2.3 equiv.) and *iso*-hexane (43 mL). The vessel was cooled to 0 °C and ethyl acetate (2.7 mL, 27.6 mmol, 3.4 equiv) was added via a syringe pump over a period of 1.5 h. The resulting suspension of lithium triethoxyaluminum hydride was cooled to −78 °C. A solution of amide **18a** (2.3 g, 8.1 mmol, 1.0 equiv) in THF (30mL) was added via a syringe pump over 5 min, and the reaction mixture was warmed to 0 °C. After being stirred for 1 h at 0 °C, the reaction mixture was transferred by cannula to an ice-cold solution of trifluoroacetic acid (5 mL, 32.5 mmol, 10 equiv) in dilute HCl (80 mL, 1.0 M, aq).The resulting biphasic mixture was diluted with dilute HCl (140 mL, 1.0 M, aq.) and the layers were separated. The aqueous layer was extracted with CH_2Cl_2 (3 × 100 mL). The combined organic layers were neutralized with $NaHCO_3$ (100 mL, sat., aq.). The aqueous layer (pH 7−8) was separated and extracted with CH_2Cl_2 (100 mL). The combined organic extracts were dried over $MgSO_4$ filtered and concentrated under reduced pressure (300 mbar) at rt. This gave the crude aldehyde **9a** as pale-yellow oil. Due to concerns about potential oxidation and racemization, the crude aldehyde **9a** was subjected to the next step without any purification. A three-neck jacketed vessel equipped with a stir bar, a gas inlet connected to an argon-vacuum manifold, a ground-glass joint thermometer adapter connected to low temperature thermometer and a rubber septum was charged with a solution of lithium perchlorate (1.1 g, 10.8 mmol, 0.9 equiv.) in Et_2O (17 mL) and a solution of **14** (409 mg, 1.1 mmol, 10 mol%) in CH_2Cl_2 (35 mL). The mixture was stirred for 0.5 h at rt and then cooled to –78 °C. DIPEA (4.5 mL, 26.0 mmol, 2.25 equiv.) was added, followed by a solution of the crude aldehyde **9a** in CH_2Cl_2-Et_2O (26 mL, 2:1). A solution of AcCl (1.3 mL, 19.0 mmol, 1.3 equiv.) in CH_2Cl_2 (9 mL) was added via syringe pump over 5 h and the resulting mixture was stirred at –78 °C for 24 h. The reaction mixture was diluted with Et_2O (50 mL) and then allowed to warm to rt. The yellow suspension obtained was then filtered through a silica plug eluting with Et_2O (100 mL) and the filtrate obtained was concentrated under reduced pressure (300 mbar) at rt. This afforded the crude β-lactone **8a** as a pale-yellow oil (> 20:1 dr by NMR), which was subjected to the next step without any purification. A three-neck jacketed vessel equipped with a stir bar, a gas inlet connected to an argon-vacuum manifold, a ground-glass joint thermometer adapter connected to low temperature thermometer and a rubber septum, was charged with a solution of TosMIC (753 mg, 3.8 mmol, 1.0 equiv.) in THF (13 mL). The solution was cooled to - 78 °C and ^{n}BuLi (2.5 mL, 4.0 mmol, 1.0 equiv., 1.6 M in *n*-hexanes) was added dropwise. The pale-yellow solution obtained was stirred at - 78 °C for 0.5 h, before a solution of the crude β-lactone **8a** in THF (20 mL) was added. The resulting mixture was stirred at –78 °C for an hour and then warmed to rt. The reaction was then quenched with HCl (20 mL, 1.0 M, aq.) and extracted with EtOAc (3 x 100 mL). The combined organic extracts were dried over $MgSO_4$, filtered and then concentrated under reduced pressure. The dark brown oily residue obtained was purified by flash column chromatography (SiO_2, 100 g, 55 mm ø, 0-100% EtOAc/iso-Hexanes, ca. 20 mL). This gave the product as an orange viscous oil (1.5 g, 52 % yield over 3 steps, 97:3 dr). ^{1}H NMR (400 MHz, $CDCl_3$): δ = 7.90 – 7.83 (m, 2H), 7.69 (s, 1H), 7.32 – 7.24 (m, 2H), 3.88 (ddt, *J* = 9.6, 6.8, 3.6 Hz, 1H), 3.27 (dd, *J* = 14.8, 9.6 Hz, 1H), 3.13 (dd, *J* = 14.8, 3.6 Hz, 1H), 2.36 (s, 3H), 2.26 – 2.03 (m, 2H), 1.94 (d, *J* = 6.6 Hz, 1H), 1.79 – 1.59 (m, 4H), 1.38 (dddd, *J* = 13.3, 8.4, 7.1, 6.8 Hz, 1H), 0.94 (d, *J* = 6.8 Hz, 3H); ^{13}C NMR (101 MHz, $CDCl_3$): δ = 155.4, 150.2, 145.5, 137.4, 137.2, 130.4, 128.6, 79.2, 76.6, 73.7, 38.3, 32.6, 31.5, 22.2, 16.9, 13.7, 3.9; $[\alpha]_D^{20}$= + 21.0 (c = 2.41, $CHCl_3$); IR (film): $\tilde{\nu}$ = 3527, 3128, 2921, 1595, 1517, 1494, 1449, 1402, 1382, 1342, 1305, 1245, 1217, 1147, 1085, 1059, 1017, 854, 815, 709, 697, 663, 601, 540, 502 cm^{-1}; HRMS-ESI+ (*m/z*): calc'd. for $C_{19}H_{23}NO_4SNa$ $[M+Na]^+$, 384.1240; found, 384.1241.

The diastereomeric purity of (2*R*,3*R*)-3-methyl-1-(4-tosyloxazol-5-yl)oct-6-yn-2-ol **7a** originally determined by NMR was confirmed by HPLC using a collection of chiral stationary phases.

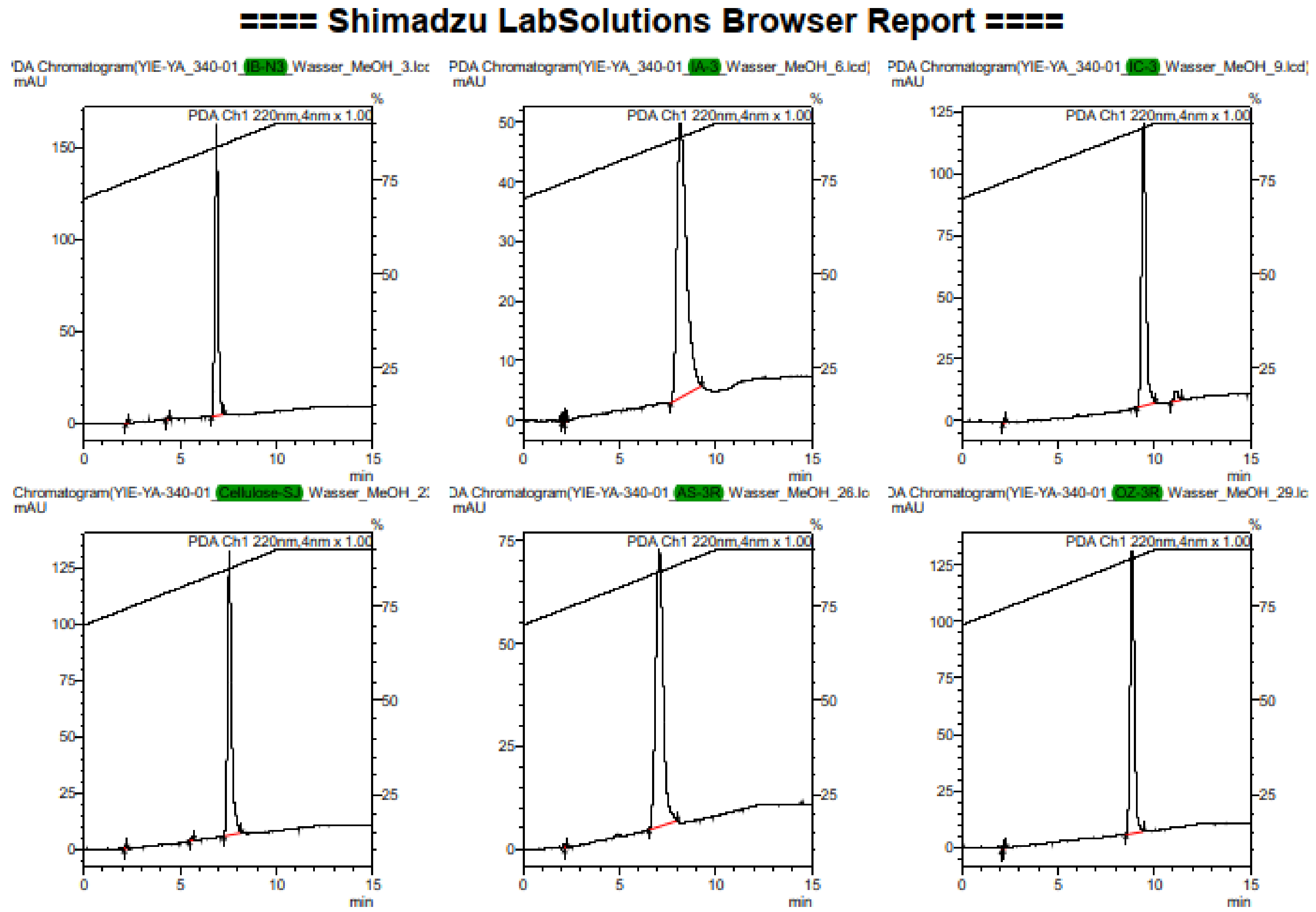

Methanol / water gradient: 70 % - 10 ' - 90 % MeOH

==== Shimadzu LabSolutions Browser Report ====

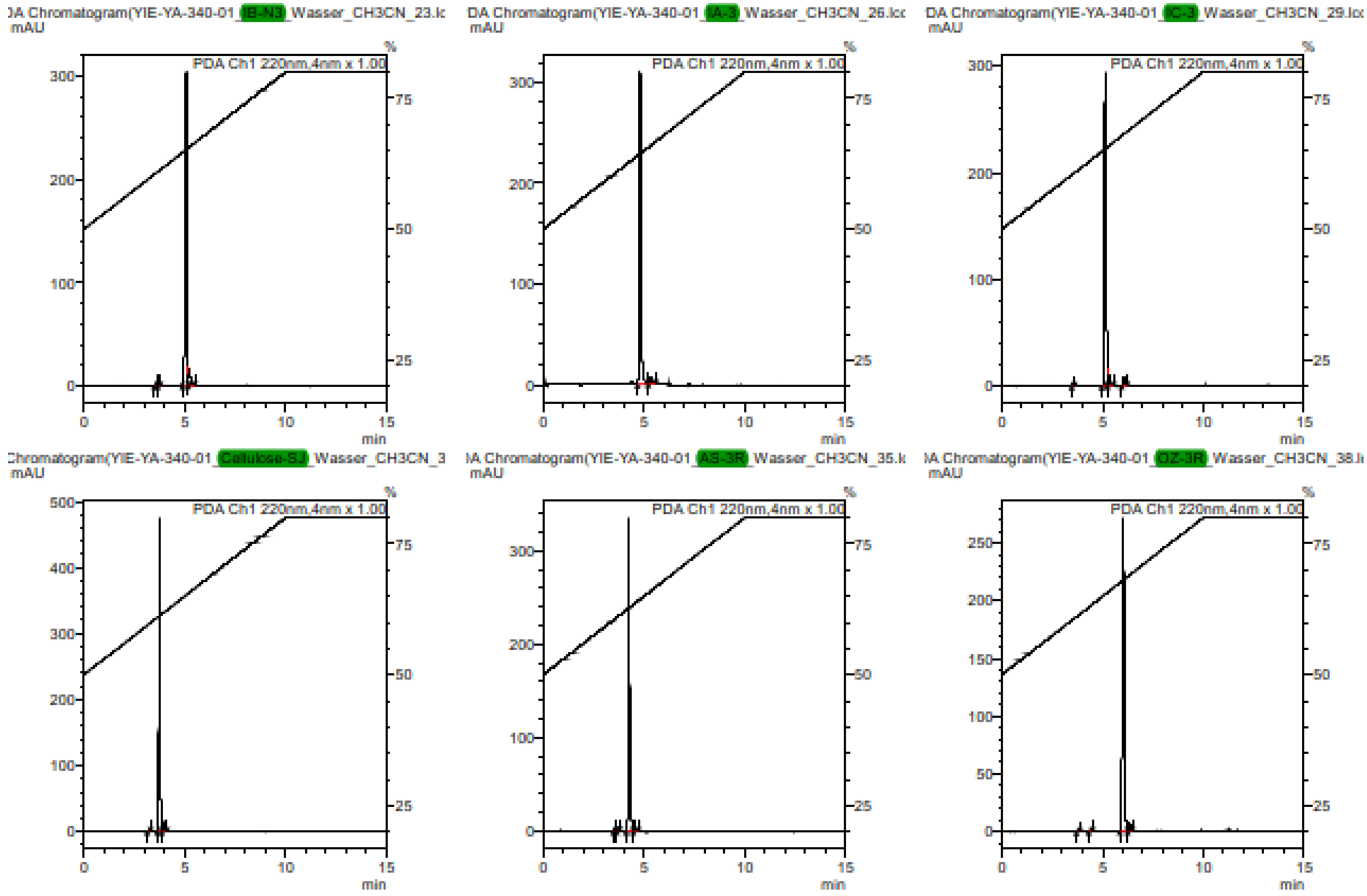

acetonitrile / water gradient: 50 % - 10 ' - 80 % ACN

Mosher esters from (2R,3R)-3-methyl-1-(4-tosyloxazol-5-yl)oct-6-yn-2-ol **7a** and Mosher ester analysis.

5-((2*R*,3*R*)-3-methyl-2-((*R*)-2,2,2-trifluoro-1-methoxy-1-phenylethoxy)oct-6-yn-1-yl)-4-tosyloxazole (15a)

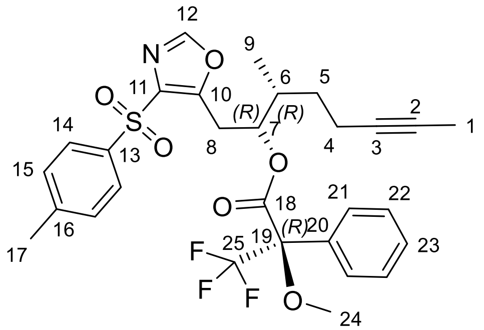

A Schleck tube equipped a magnetic stir bar was charged with a solution of (2*R*,3*R*)-3-methyl-1-(4-tosyloxazol-5-yl)oct-6-yn-2-ol **7a** (45.0 mg, 125 µmol, 1.5 equiv.) in CH_2Cl_2 (1 mL), DMAP (1.0 mg, 6.3 µmol, 10.0 mol%) and TEA (35 µL, 249 µmol, 3.0 equiv.). (S)-(+)-MTPACl (11.8 µL, 63 µmol) was added and the resulting mixture was stirred overnight at rt. After that the solvent was evaporated under reduced pressure. The residue was purified by flash column chromatography (SiO_2, 10 g, 20 mm ø, 0-50% EtOAc/iso-Hexanes, ca. 5 mL). This gave the product as a pale yellow oil (28 mg, 77 %).^{1}H NMR (600 MHz, $CDCl_3$): δ = 7.94 – 7.90 (m, 2H), 7.44 (s, 1H), 7.38 – 7.32 (m, 6H), 7.31 – 7.27 (m, 2H), 5.49 (ddd, *J* = 9.2, 4.4, 3.2 Hz, 1H), 3.51 – 3.47 (m, 4H), 3.31 (dd, *J* = 15.3, 9.2 Hz, 1H), 2.43 (s, 3H), 2.30 – 2.22 (m, 1H), 2.18 – 2.07 (m, 1H), 1.71 (t, *J* = 2.5 Hz, 3H), 1.65 (dddd, *J* = 13.2, 8.3, 6.9, 4.9 Hz, 1H), 1.35 (dddd, *J* = 13.2, 9.2, 6.9, 6.1 Hz, 1H), 1.05 (d, *J* = 6.9 Hz, 3H); ^{13}C NMR (151 MHz, $CDCl_3$): δ = 166.0, 152.5, 149.9, 145.1, 136.8, 136.7, 131.8, 129.9, 129.5, 129.5, 128.5, 128.4, 128.24, 128.21, 127.13, 127.06, 126.0, 123.14 (q, *J* = 288.7 Hz), 84.6, 77.7, 77.2, 77.0, 76.8, 76.5, 55.4, 35.2, 31.0, 27.1, 21.7, 16.3, 14.0, 3.4; IR (film): $\tilde{\nu}$ =3130, 2923, 1746, 1595, 1515, 1495, 1451, 1386, 1329, 1291, 1248, 1169, 1148, 1120, 1083, 1018, 998, 933, 815, 766, 719, 697, 662, 601, 540 cm^{-1}; $[\alpha]_D^{20}$= + 9.7 (c = 1.31, $CHCl_3$); HRMS-ESI+ (*m/z*): calc'd. for $C_{29}H_{30}F_3NO_6SNa$ $[M+Na]^+$, 600.1638; found, 600.1640.

5-((2R,3R)-3-methyl-2-((S)-2,2,2-trifluoro-1-methoxy-1-phenylethoxy)oct-6-yn-1-yl)-4-tosyloxazole (16a)

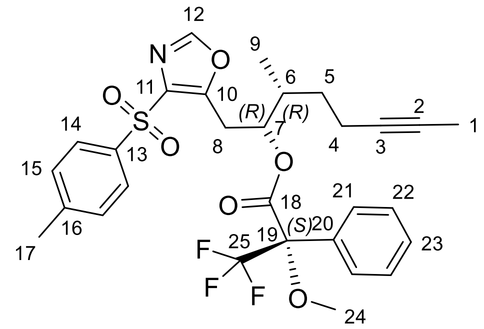

A Schleck tube equipped a magnetic stir bar was charged with a solution of (2*R*,3*R*)-3-methyl-1-(4-tosyloxazol-5-yl)oct-6-yn-2-ol **7a** (45.0 mg, 125 µmol, 1.5 equiv.) in CH_2Cl_2 (1 mL), DMAP (1.0 mg, 6.3 µmol, 10.0 mol%) and TEA (35 µL, 249 µmol, 3.0 equiv.). (R)-(-)-MTPACl (11.8 µL, 63 µmol) was added and the resulting mixture was stirred overnight at rt. After that the solvent was evaporated under reduced pressure. The residue was purified by flash column chromatography (SiO_2, 10 g, 20 mm ø, 0-50% EtOAc/iso-Hexanes, ca. 5 mL). This gave the product as a pale yellow oil (26 mg, 72 %).^{1}H NMR (600 MHz, $CDCl_3$): δ = 7.94 – 7.90 (m, 2H), 7.44 (s, 1H), 7.38 – 7.32 (m, 6H), 7.31 – 7.27 (m, 2H), 5.49 (ddd, *J* = 9.2, 4.4, 3.2 Hz, 1H), 3.51 – 3.47 (m, 4H), 3.31 (dd, *J* = 15.3, 9.3 Hz, 1H), 2.43 (s, 3H), 2.30 – 2.22 (m, 1H), 2.18 – 2.07 (m, 1H), 1.71 (t, *J* = 2.5 Hz, 3H), 1.65 (dddd, *J* = 13.3, 8.3, 7.0, 4.9 Hz, 1H), 1.35 (dddd, *J* = 13.2, 9.2, 6.9, 6.1 Hz, 1H), 1.05 (d, *J* = 6.9 Hz, 3H); ^{13}C NMR (151 MHz, $CDCl_3$): δ = 166.0, 152.5, 149.9, 145.1, 136.8, 136.7, 131.8, 129.9, 129.5, 129.5, 128.5, 128.4, 128.2, 128.2, 127.1, 127.1, 126.0, 123.3 (q, *J* = 288.5 Hz), 84.6, 77.7, 77.2, 77.0, 76.8, 76.5, 55.4, 35.2, 31.0, 27.1, 21.7, 16.3, 14.0, 3.4; IR (film): $\tilde{\nu}$ = 3129, 2923, 1746, 1595, 1515, 1494, 1451, 1385, 1329, 1291, 1248, 1169, 1148, 1120, 1083, 1018, 997, 932, 815, 765, 719, 697, 662, 601, 540 cm^{-1}; $[\alpha]_D^{20}$= - 14.1 (c = 1.22, $CHCl_3$); HRMS-ESI+ (*m/z*): calc'd. for $C_{29}H_{30}F_3NO_6SNa$ $[M+Na]^+$, 600.1638; found, 600.1641.

Table S-1. **Mosher ester analysis of (2*R*,3*R*)-3-methyl-1-(4-tosyloxazol-5-yl)oct-6-yn-2-ol 7a, arbitrary numbering scheme as shown below.**

Assignment	(S)-X [ppm]	(R)-X [ppm]	Δ(δ(S-R)) [ppm]
8a	3.51	3.49	0.02
8b	3.46	3.31	0.15
6	2.02	2.10	-0.08
5a	1.51	1.65	-0.14
5b	1.28	1.35	-0.07
4a	2.18	2.26	-0.08
4b	2.09	2.14	-0.05
9	1.01	1.05	-0.04

1H (CDCl3, 600.22 MHz)

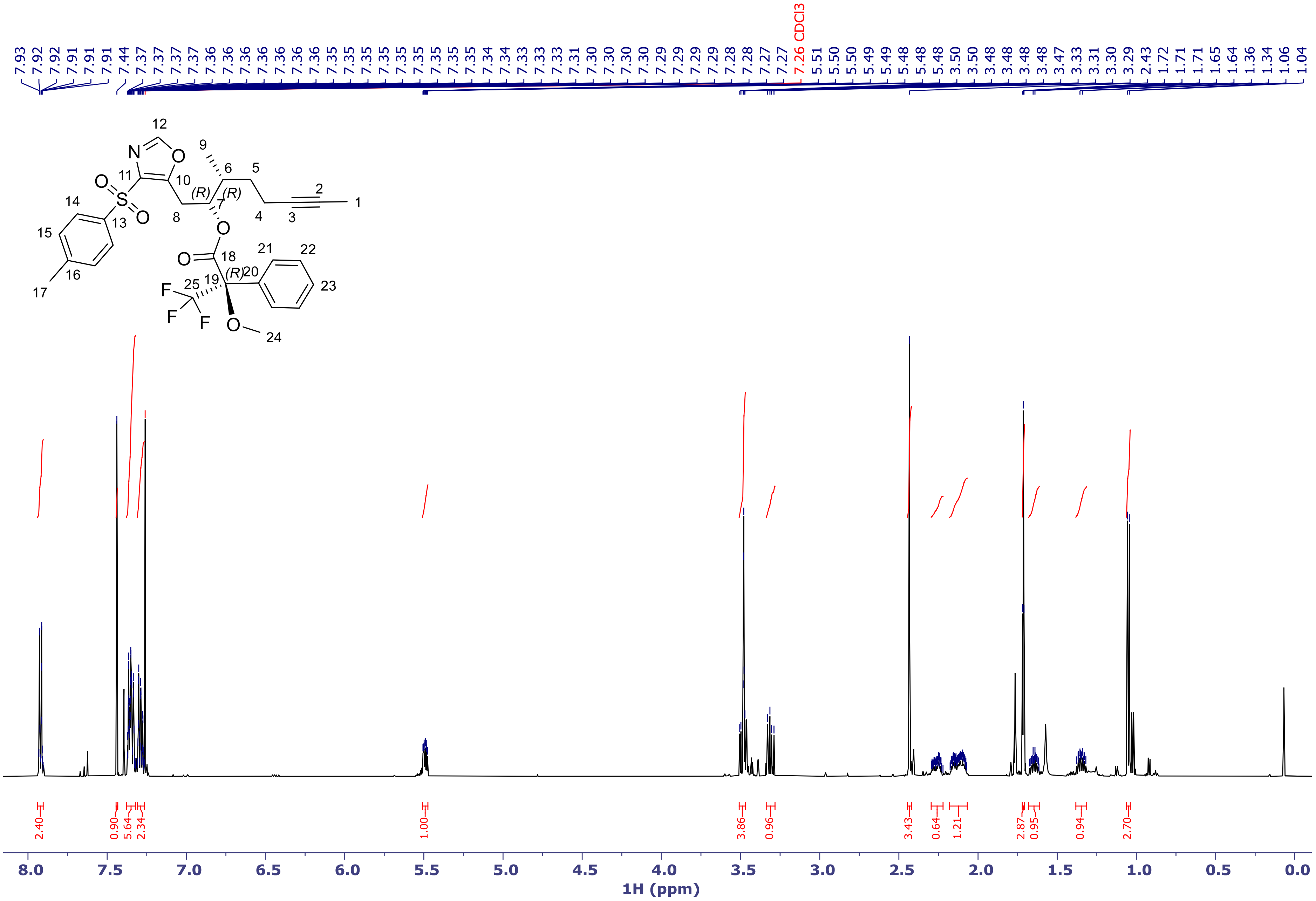

13C (CDCl3, 150.94 MHz)

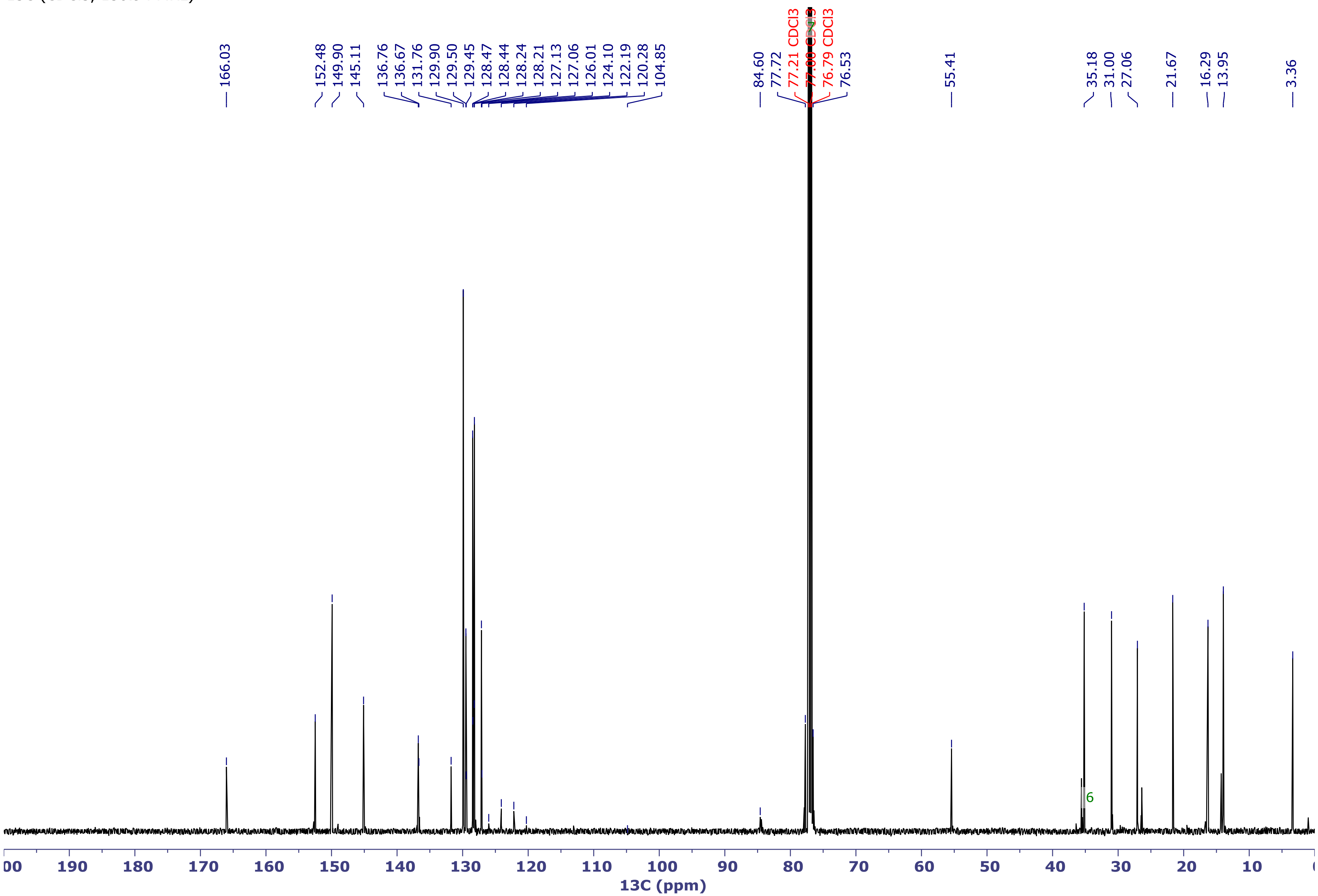

1H (CDCl3, 600.22 MHz)

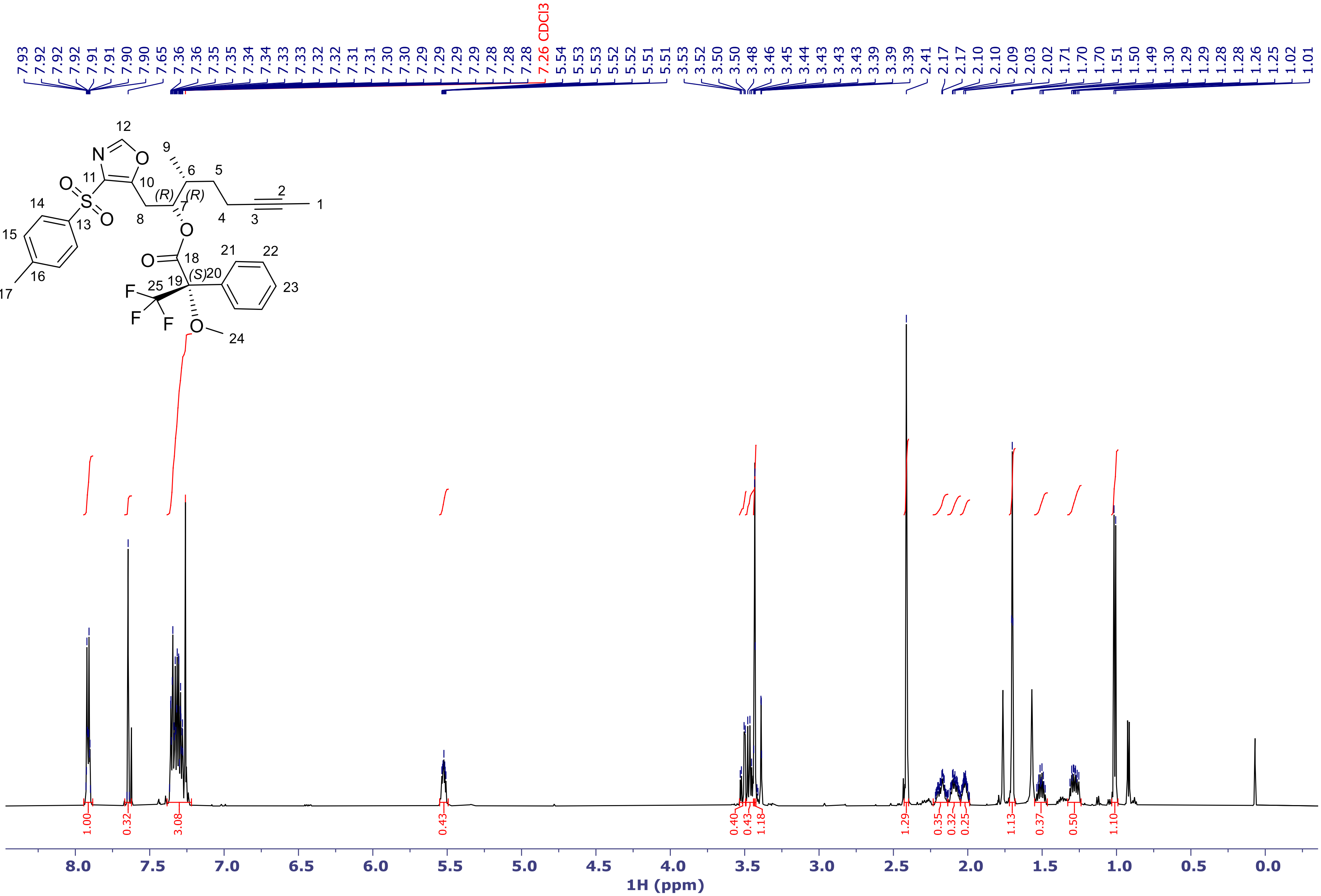

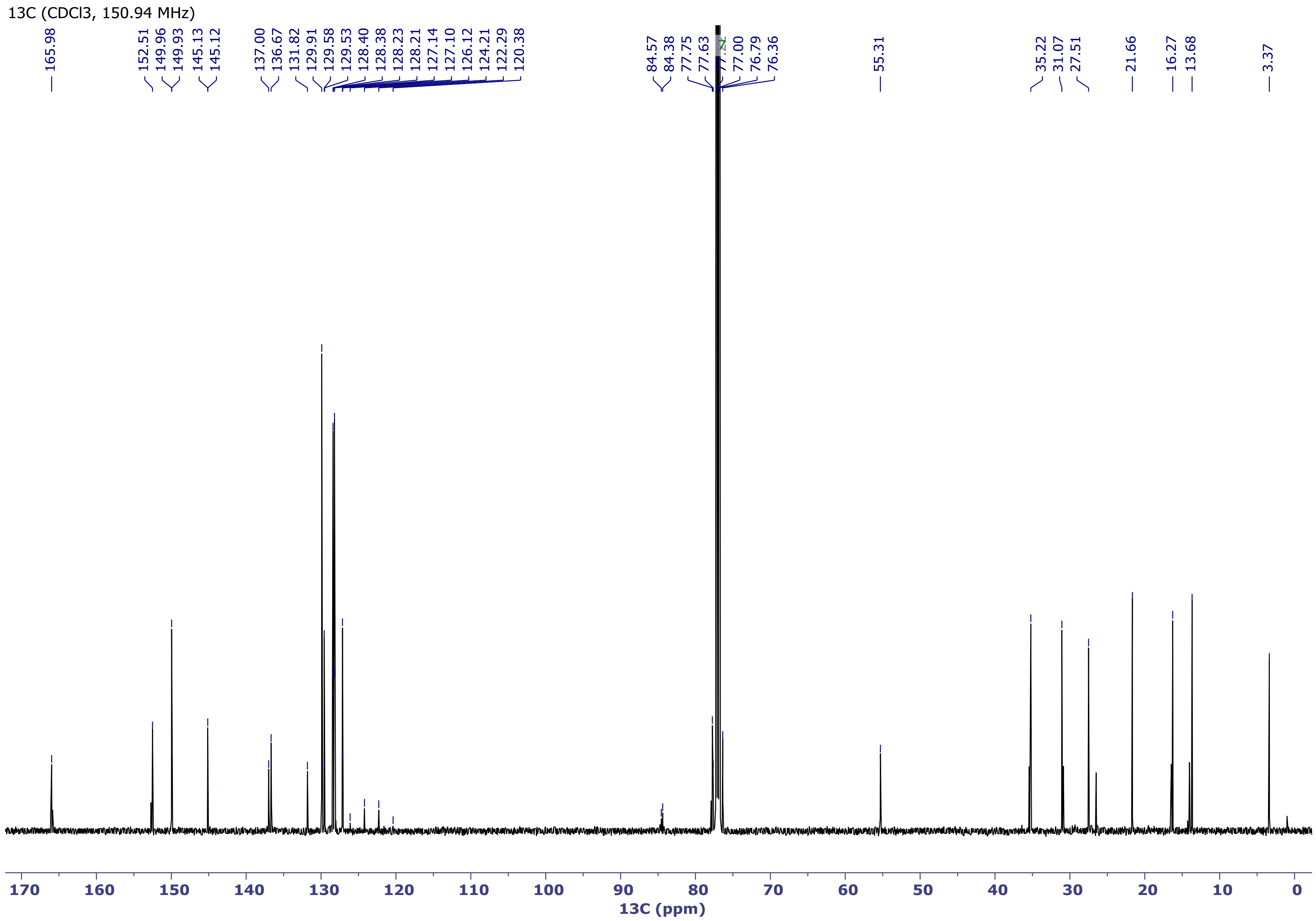
13C (CDCl3, 150.94 MHz)
165.98
152.51
149.96
149.93
145.13
145.12
137.00
136.67
131.82
129.91
129.58
129.53
128.40
128.38
128.23
128.21
127.14
127.10
126.12
124.21
122.29
120.38
84.57
84.38
77.75
77.63
77.00
76.79
76.36
55.31
35.22
31.07
27.51
21.66
16.27
13.68
3.37
13C (ppm)

(*R*)-*N*-((1*S*,2*S*)-1-hydroxy-1-phenylpropan-2-yl)-*N*,2-dimethylnon-7-ynamide (18b)

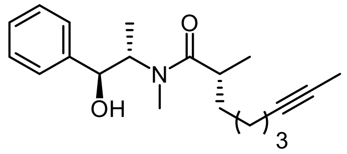

A three-neck jacketed vessel equipped with a stir bar, a gas inlet connected to an argon-vacuum manifold, a ground-glass joint thermometer adapter connected to low temperature thermometer and a rubber septum, was charged with a solution of lithium chloride (4.6 g, 108.5 mmol, 6.0 equiv.) and diisopropylamine (5.7 mL, 40.7 mmol, 2.3 equiv.) in THF (30 mL). Upon cooling of the mixture to 0 °C, ^{n}BuLi (23.5 mL, 37.6 mmol, 2.1 equiv., 1.6 M in *n*-hexane) was added dropwise and the mixture was stirred at this temperature for 1 h. After that the reaction mixture was cooled to - 78 °C and a solution of N-((1*S*,2*S*)-1-hydroxy-1-phenylpropan-2-yl)-*N*-methylpropionamide *ent*-**17** (4.0 g, 18.1 mmol, 1.0 equiv.) in THF (30 mL) was added dropwise. The mixture was then stirred for 1 h at - 78 °C, before it was warmed to 0 °C. 7-iodohept-2-yne **S3** (6.0 g, 27.1 mmol. 1.5 equiv.) was added and the resulting mixture was then stirred for 2 h at 0 °C. The reaction mixture was then quenched with NH_4Cl (100 mL, sat. aq.) and then extracted with EtOAc (3 x 100 mL). The combined organic extracts were dried over $MgSO_4$, filtered and then concentrated under reduced pressure. The orange oily residue obtained was then purified by flash column chromatography (SiO_2, 100 g, 55 mm ø, 0-100% EtOAc/iso-Hexanes, ca. 20 mL). This afforded the product as a thick pale yellow syrup (5.6 g, 98 %). ^{1}H NMR (600 MHz, DMSO, mixture of rotamers (1:1)): δ 7.35 – 7.30 (m, 4H, rotamer a), 7.30 – 7.25 (m, 4H, rotamer b), 7.25 – 7.22 (m, 1H, rotamer a), 7.21 – 7.18 (m, 1H, rotamer b), 5.42 (d, *J* = 3.9 Hz, 1H, rotamer a), 5.29 (d, *J* = 4.2 Hz, 1H, rotamer b), 4.68 (s, 1H, rotamer b), 4.54 (dd, *J* = 7.7, 4.6 Hz, 1H, rotamer a), 4.51 (dd, *J* = 7.5, 3.9 Hz, 1H, rotamer b), 3.94 (p, *J* = 6.9 Hz, 1H, rotamer a), 2.83 (s, 3H, rotamer b), 2.73 (s, 3H, rotamer a), 2.68 – 2.57 (m, 2H, rotamer a, rotamer b), 2.03 (qt, *J* = 6.8, 2.6 Hz, 4H, rotamer a, rotamer b), 1.71 (t, *J* = 2.6 Hz, 3H, rotamer a), 1.67 (t, *J* = 2.6 Hz, 3H, rotamer b), 1.52 – 1.42 (m, 2H, rotamer a, rotamer b), 1.37 – 1.27 (m, 4H, rotamer a, rotamer b), 1.19 – 1.08 (m, 4H, rotamer a, rotamer b), 0.95 (d, *J* = 6.8 Hz, 3H, rotamer a), 0.88 (d, *J* = 6.9 Hz, 3H, rotamer a), 0.85 (d, *J* = 7.0 Hz, 3H, rotamer b), 0.81 (d, *J* = 6.8 Hz, 3H, rotamer b); ^{13}C NMR (151 MHz, DMSO, mixture of rotamers): δ = 176.2 (rotamer a), 175.9 (rotamer b), 144.1 (rotamer b), 144.0 (rotamer a), 128.5 (rotamer a), 128.2 (rotamer b), 127.7 (rotamer a), 127.4 (rotamer b), 127.3 (rotamer a), 127.1 (rotamer b), 79.9 (rotamer a), 79.7 (rotamer b), 76.1 (rotamer b), 76.0 (rotamer a), 74.4v(rotamer a), 74.3 (rotamer b), 57.2 (rotamer a), 53.6 (rotamer b), 35.2 (rotamer b), 35.0 (rotamer a), 33.7 (rotamer b), 33.4 (rotamer a), 30.4 (rotamer b), 29.4 (rotamer a), 29.1 (rotamer b), 27.7 (rotamer a), 26.6 (rotamer b), 26.5 (rotamer a), 18.6 (rotamer a), 18.5 (rotamer b), 18.4 (rotamer a), 17.9(rotamer b), 16.0 (rotamer a), 14.5 (rotamer b), 3.6 (rotamer a), 3.5 (rotamer b); $[\alpha]_D^{20}$= + 44.0 (c = 1.05, EtOH); IR (film): $\tilde{\nu}$ = 3367, 3061, 3029, 2968, 2934, 2859, 1617, 1452, 1409, 1374, 1308, 1199, 1109, 1083, 1051, 1027, 756, 702, 671, 666, 591, 549, 526 cm^{-1}; HRMS-GC-EI (*m/z*): calc'd. for $C_{20}H_{25}NO_3$ $[M]^{+\cdot}$, 327. 1828; found, 327. 1830.

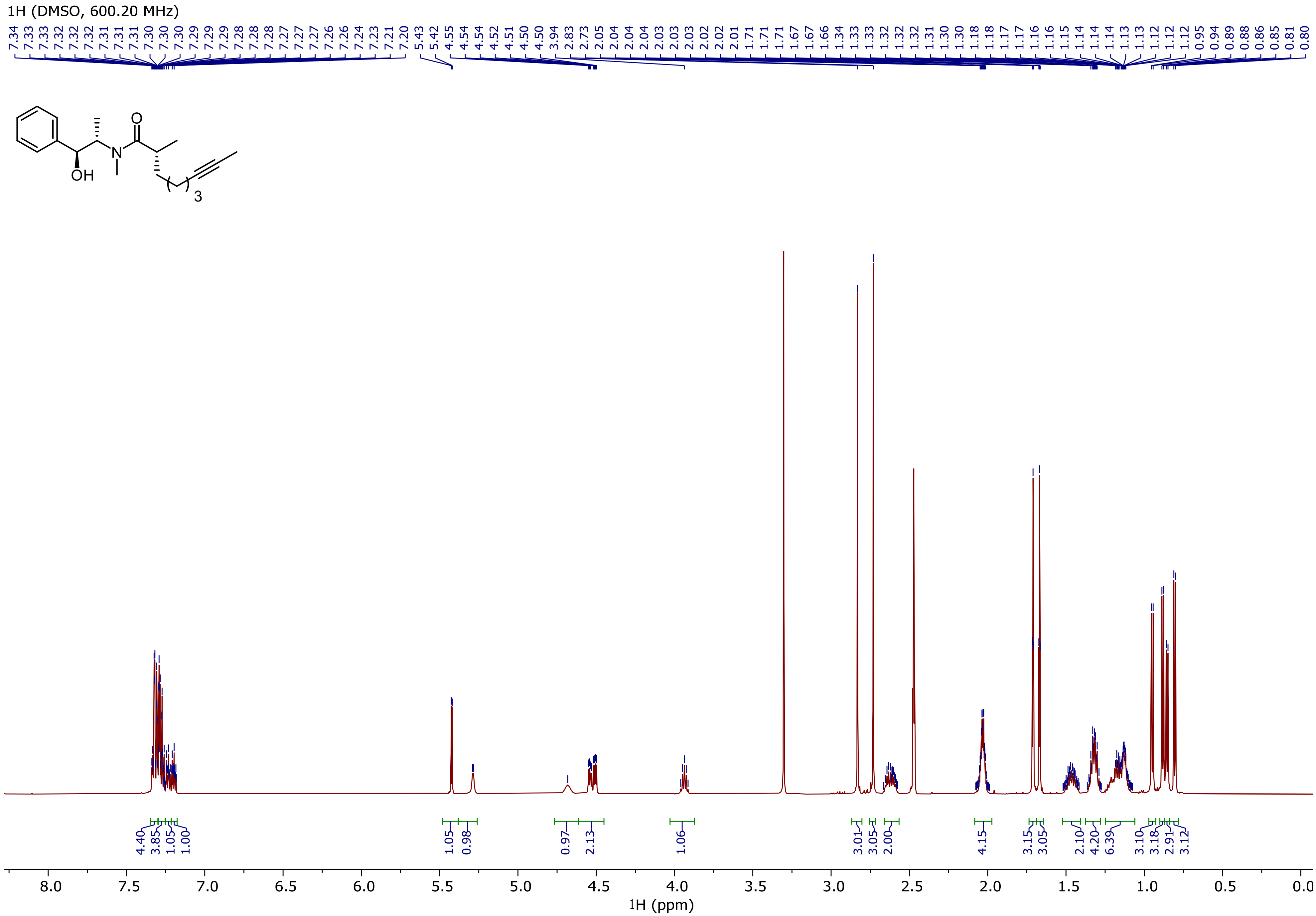
1H (DMSO, 600.20 MHz)
7.34 7.33 7.33 7.32 7.32 7.32 7.31 7.31 7.30 7.30 7.30 7.29 7.29 7.28 7.28 7.27 7.27 7.26 7.24 7.23 7.21 7.20 5.43 5.42 4.55 4.54 4.54 4.52 4.51 4.50 4.50 3.94 2.83 2.73 2.05 2.04 2.04 2.04 2.03 2.03 2.02 2.02 2.01 1.71 1.71 1.71 1.67 1.67 1.66 1.34 1.33 1.33 1.32 1.32 1.32 1.31 1.30 1.30 1.18 1.18 1.17 1.17 1.16 1.16 1.15 1.14 1.14 1.14 1.13 1.13 1.12 1.12 1.12 0.95 0.94 0.89 0.88 0.86 0.85 0.81 0.80
OH
N
O
3
4.40 3.85 1.05 1.00 1.05 0.98 0.97 2.13 1.06 3.01 3.05 2.00 4.15 3.15 3.05 2.10 4.20 6.39 3.10 3.18 2.91 3.12
8.0 7.5 7.0 6.5 6.0 5.5 5.0 4.5 4.0 3.5 3.0 2.5 2.0 1.5 1.0 0.5 0.0
1H (ppm)

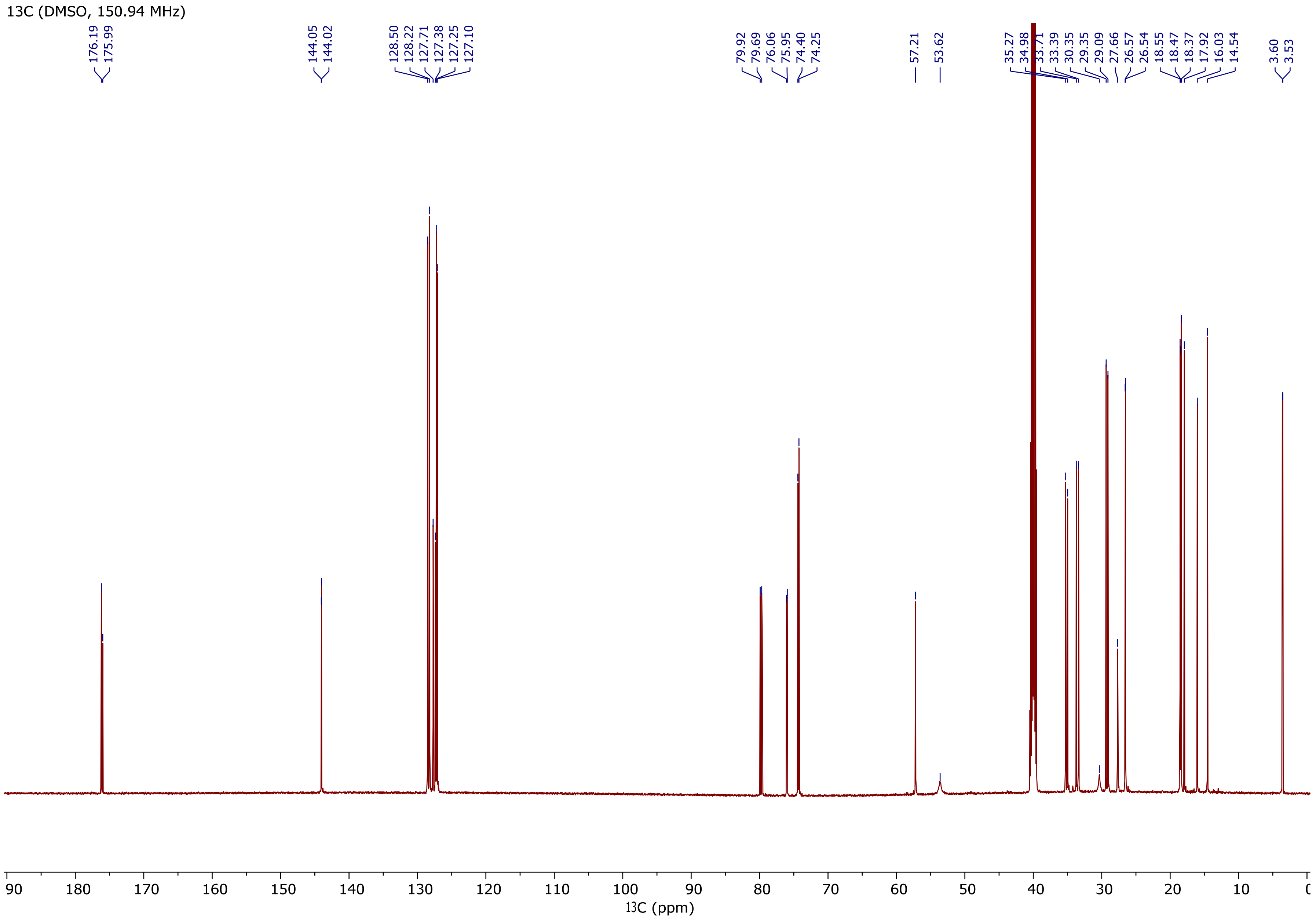
13C (DMSO, 150.94 MHz)
176.19
175.99
144.05
144.02
128.50
128.22
127.71
127.38
127.25
127.10
79.92
79.69
76.06
75.95
74.40
74.25
57.21
53.62
35.27
34.98
33.71
33.39
30.35
29.35
29.09
27.66
26.57
26.54
18.55
18.47
18.37
17.92
16.03
14.54
3.60
3.53
13C (ppm)

Amide **19b** was epimerized by stirring with trifluoroacetic acid (10 equiv.) in THF at reflux for 1 h (effecting N → O acyl transfer as well as α-epimerization), followed by neutralization with sat. $NaHCO_3$ solution at 23 °C for 24 h (causing O → N acyl transfer). The optical purity of diastereomerically pure amide **19b** was determined (71.4: 1 dr) by HPLC on a crude sample of the amide **19b** using a chiral stationary phase (Chiralpak AS-3, 150 mm, ∅ 4.6 mm).

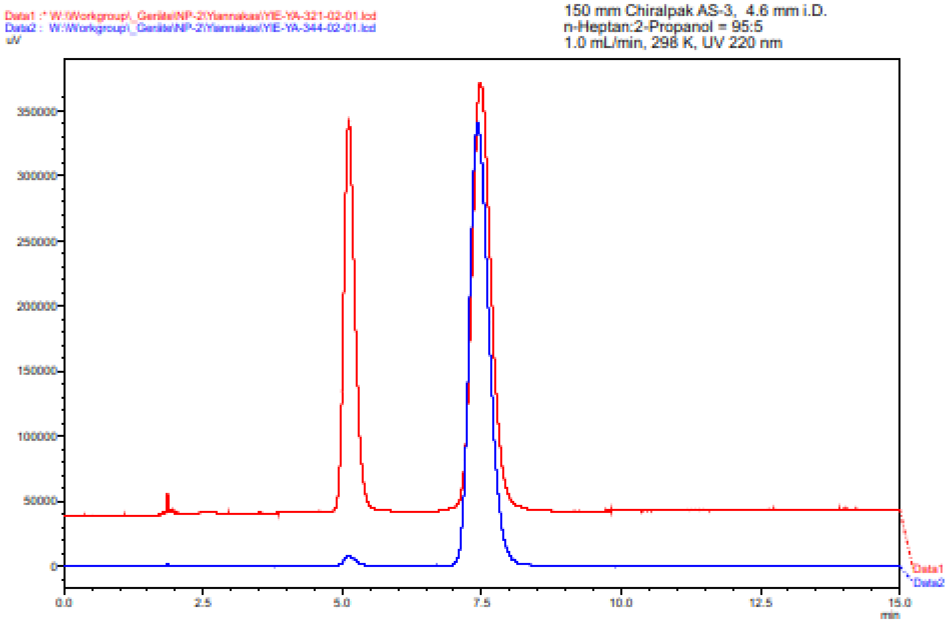

Säulenauswahlventil
<<Oven>>
Valve L : 5
Valve R : 0

1 µl YIE-YA-321-02 (in n-Heptan)
150 mm Chiralpak AS-3, 4.6 mm i.D., Säule 2
n-Heptan / 2-Propanol = 95:5
1.0 mL/min, 10.8 MPa, 298 K
UV, 220 nm

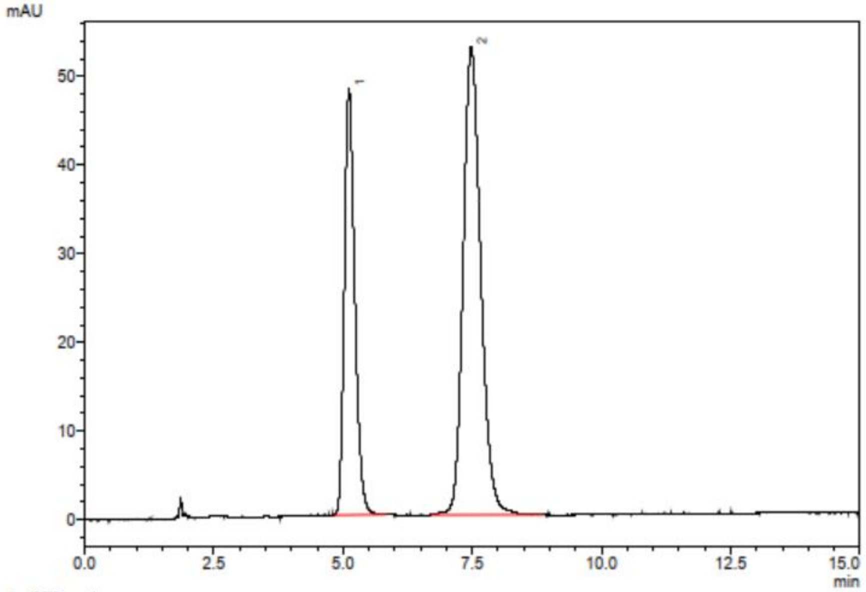

1 220nm,4nm

PDA Ch1 220nm

Peak #	Ret. Time	Area %	Name
1	5.12	35.08	1st epimer
2	7.48	64.92	2nd epimer
Total		100.00	

1 µl YIE-YA-344-02 (in n-Heptan)
150 mm Chiralpak AS-3, 4.6 mm i.D., Säule 2
n-Heptan / 2-Propanol = 95:5
1.0 mL/min, 10.8 MPa, 298 K
UV, 220 nm

Säulenauswahlventil
<<Oven>>
Valve L : 5
Valve R : 0

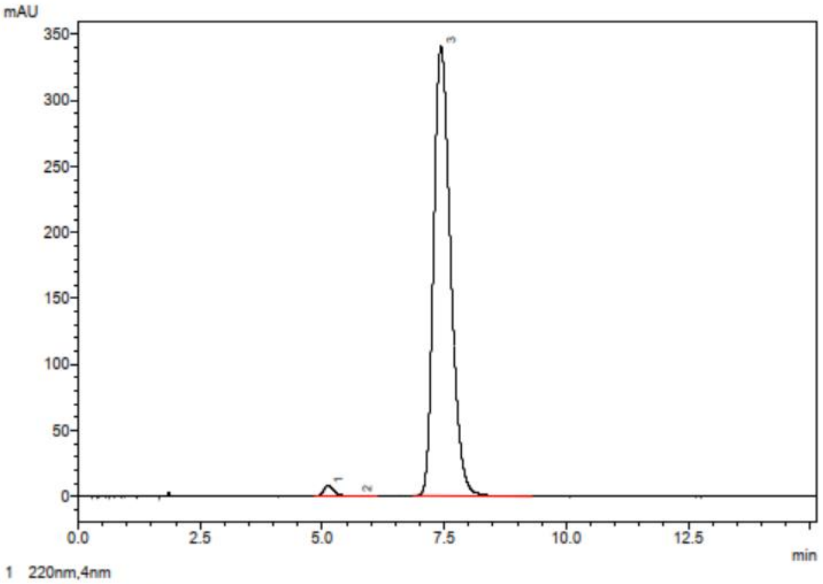

1 220nm,4nm

PDA Ch1 220nm

Peak #	Ret. Time	Area %	Name
1	5.12	1.38	1st epimer
2	5.71	0.13	
3	7.43	98.49	2nd epimer
Total		100.00	

dr = 71.4 : 1

(*R*)-4-benzyl-3-(non-7-ynoyl)oxazolidin-2-one (11b)

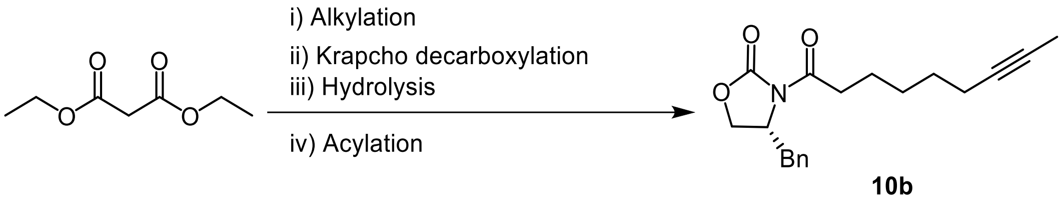

<u>Step 1 (Alkylation):</u>

A two-neck round bottom flask equipped with a stir bar, rubber septum and an argon bridge was charged with NaH (338 mg, 14.1 mmol, 1.1 equiv.) and DMF (37.5 mL). The resulting suspension was cooled to 0°C over an ice-bath. Diethyl malonate (2.2 g, 13.4 mmol) was added, and the resulting mixture was stirred for 0.5 h at 0 °C. After that 7-iodohept-2-yne **S3** (3.1 g, 14.1 mmol, 1.1 equiv.) was added and reaction mxture was allowed to warm to rt and then stirred overnight. The reaction mixture was quenched with brine (200 mL) and then extracted with Et_2O (4 x 100 mL). The combined organic extracts were then dried over $MgSO_4$, filtered and then concentrated under reduced pressure. The yellow oil obtained was carried forward without any purification.

<u>Step 2 (Krapcho Decarboxylation):</u>

A round bottom flask equipped with a reflux condenser and a stirbar was charged with a solution of the crude diethyl 2-(hept-5-yn-1-yl)malonate in DMSO (24 mL), water (100 uL, 5.6 mmol, 0.4 equiv.) and LiCl (1.3 g, 29.7 mmol, 2.2 equiv.). The resulting mixture was heated at 160 °C overnight. Upon cooling to rt, the reaction mixture was diluted with brine (250 mL) and extracted with Et_2O (3 x 100 mL). The combined organic extracts were dried over $MgSO_4$, filtered and then concentrated under reduced pressure. The brown oil obtained was then carried foward without any purification.

<u>Step 3 (Hydrolysis):</u>

A round bottom flask equipped with a stir bar was charged with a solution of the crude ethyl non-7-ynoate in THF (115 mL) and the flask was cooled to 0 °C. A solution of LiOH (2.3 g, 96.8 mmol, 3.0 equiv.) in water (115 mL) was added and the resulting mixture was allowed to warm to rt and then stirred overnight. The THF was removed under reduced pressure and the aqueous fraction obtained was acidified to pH 1 with conc. HCl. The resulting mixture was extracted with EtOAc (3 x 100 mL) and the combined organic extracts were dried over $MgSO_4$, filtered and then concentrated under reduced pressure. This gave the acid **17** as a white solid, which was used directly in the next step.

<u>Step 4 (Acylation):</u>

A three-neck jacketed glass reaction vessel equipped with a magnetic stir bar, a pressure equalizing addition funnel and a gas inlet connected to an argon-vacuum manifold, was charged with solution of the crude 7-nonynoic acid 48 (6.0 g, 12.8 mmol) in THF (70 mL) and TEA (4.8 mL, 34.3 mmol, 2.6 equiv.). The reaction mixture was then cool to - 10°C and PvCl (1.6 mL, 12.8 mmol, 1.0 equiv.) dropwise. The white slurry immediately formed was stirred at this temperature for 1 h. Then, a solution of (*R*)-4-benzyloxazolidin-2-one (2.2 g, 12.2 mmol, 1.0 equiv.) and TEA (2.3 mL, 16.8 mmol, 3.0 equiv.) in THF (7.4 mL) was added. The resulting mixture was allowed to warm to rt and then stirred overnight. The reaction

mixture was quenched with $NaHCO_3$ (100 mL, sat. aq.) and extracted with EtOAc (3 x 100 mL). The combined organic layers were washed with brine (100 mL), dried $MgSO_4$, filtered and then concentrated under reduced pressure. The residue was purified by flash column chromatography (SiO_2, 100 g, 55 mm ø, 0-50% EtOAc/iso-Hexanes, ca. 20 mL). This gave the product as a pale yellow oil (1.0 g, 24 %). ^{1}H NMR (400 MHz, $CDCl_3$): δ = 7.32 – 7.22 (m, 2H), 7.24 – 7.19 (m, 1H), 7.16 – 7.10 (m, 2H), 4.67 – 4.52 (m, 1H), 4.17 – 4.09 (m, 2H), 3.23 (dd, *J* = 13.3, 3.3 Hz, 1H), 2.97 – 2.78 (m, 2H), 2.76 – 2.64 (m, 1H), 2.08 (tq, J = 7.0, 2.6 Hz, 2H), 1.70 (t, J = 2.6 Hz, 3H), 1.68 – 1.59 (m, 2H), 1.52 – 1.35 (m, 4H).; ^{13}C NMR (151 MHz, $CDCl_3$): δ = 173.3, 153.5, 135.3, 129.4, 128.9, 127.4, 79.0, 75.6, 66.2, 55.2, 37.9, 34.9, 28.8, 28.3, 23.8, 18.6, 3.5; IR (film): $\tilde{\nu}$ = 3029, 2923, 2859, 1776, 1698, 1604, 1497, 1480, 1454, 1386, 1351, 1329, 1291, 1212, 1198, 1156, 1111, 1078, 1051, 1016, 921, 844, 762, 746, 703, 621, 595, 504 cm^{-1}; $[\alpha]_D^{20}$= -43.9 (c = 1.48, $CHCl_3$); HRMS-ESI+ (*m/z*): calc'd. for $C_{19}H_{23}NO_3Na$ $[M+Na]^+$,336.1570; found 336.1566.

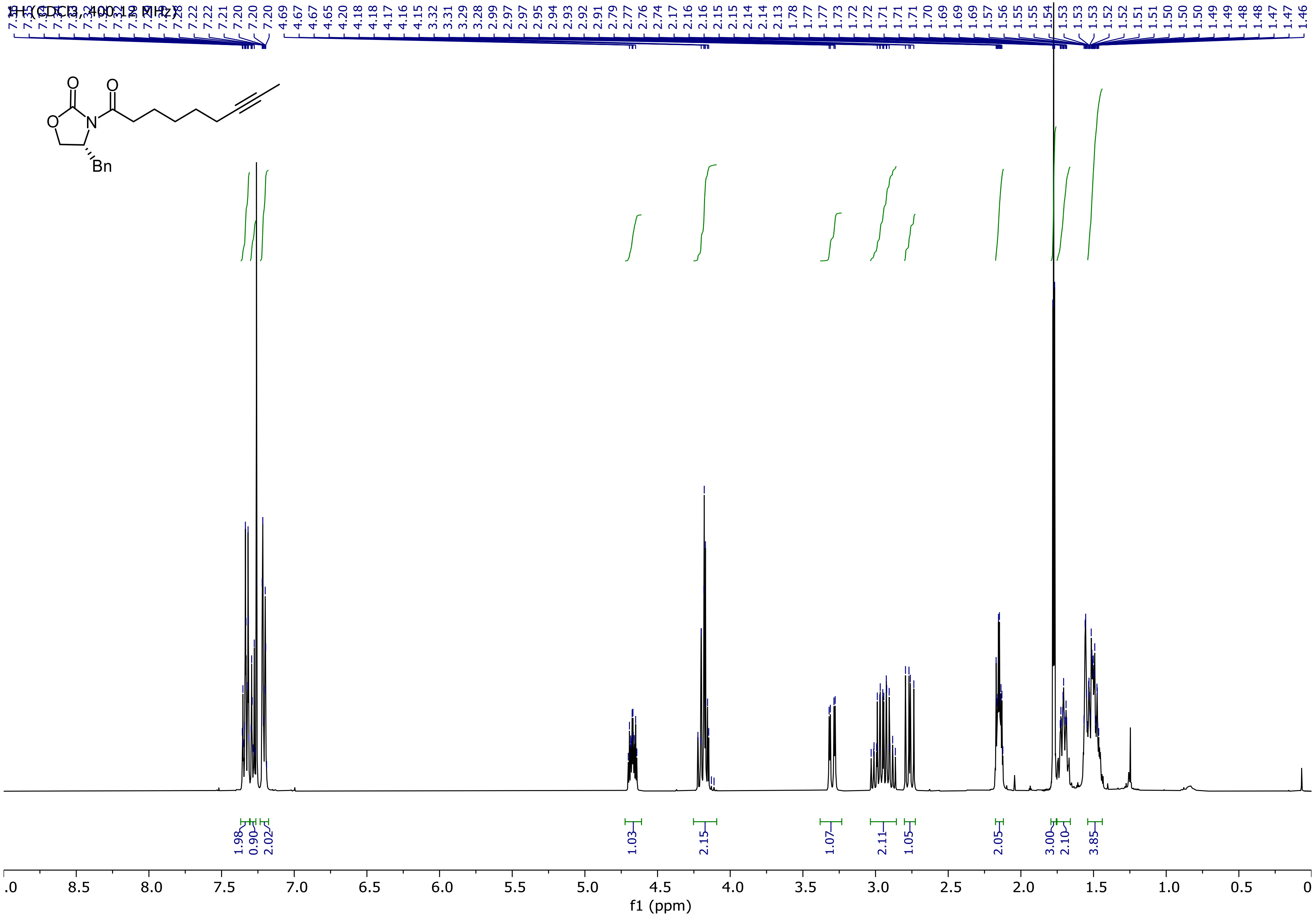
4.69 4.67 4.65 4.20 4.18 4.18 4.17 4.16 4.15 3.32 3.31 3.29 3.28 2.99 2.97 2.97 2.95 2.94 2.93 2.92 2.91 2.79 2.77 2.76 2.74 2.17 2.16 2.16 2.15 2.15 2.14 2.14 2.13 1.78 1.77 1.77 1.73 1.72 1.72 1.71 1.71 1.71 1.70 1.69 1.69 1.69 1.57 1.56 1.55 1.55 1.54 1.53 1.53 1.53 1.52 1.52 1.51 1.51 1.50 1.50 1.50 1.49 1.49 1.48 1.48 1.47 1.47 1.46
Bn
1.98 0.90 2.02 1.03 2.15 1.07 2.11 1.05 2.05 3.00 2.10 3.85
8.5 8.0 7.5 7.0 6.5 6.0 5.5 5.0 4.5 4.0 3.5 3.0 2.5 2.0 1.5 1.0 0.5 0
f1 (ppm)

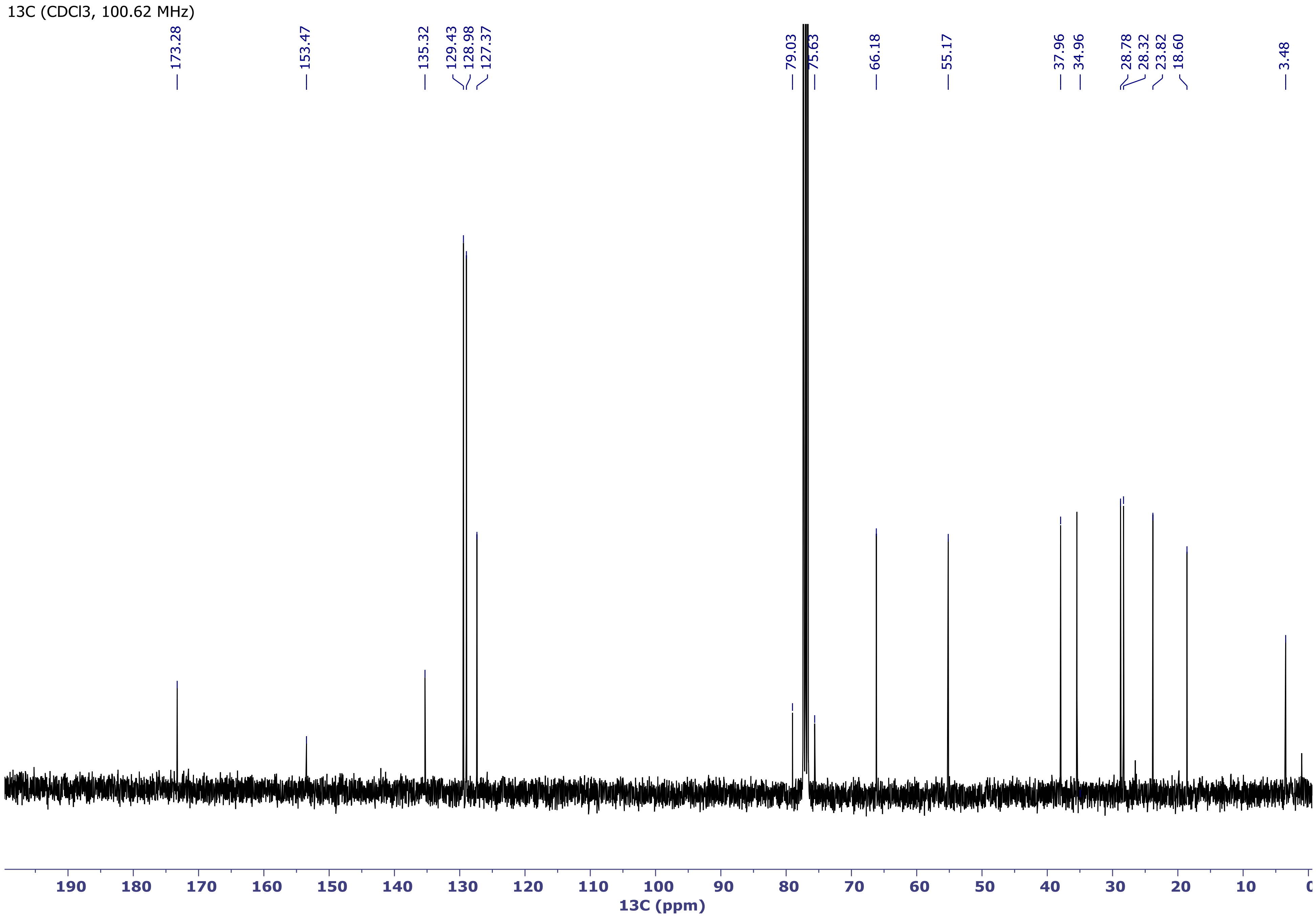
13C (CDCl3, 100.62 MHz)
173.28
153.47
135.32
129.43
128.98
127.37
79.03
75.63
66.18
55.17
37.96
34.96
28.78
28.32
23.82
18.60
3.48
190 180 170 160 150 140 130 120 110 100 90 80 70 60 50 40 30 20 10
13C (ppm)

(R)-4-benzyl-3-((R)-2-methylnon-7-ynoyl)oxazolidin-2-one (11b)

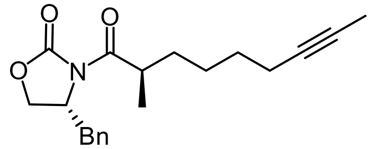

A three-neck jacketed vessel equipped with a stir bar, a gas inlet connected to an argon-vacuum manifold, a ground-glass joint thermometer adapter connected to low temperature thermometer and a rubber septum was charged with a solution of (R)-4-benzyl-3-(non-7-ynoyl)oxazolidin-2-one **10b** (4.0 g, 12.8 mmol) in THF (74.5 mL). The reaction mixture was cooled to –78 °C. A solution of NaHMDS (13.1 mL, 19.1 mmol, 1.5 equiv., 1.5 M solution in THF) was added dropwise. The resulting pale yellow mixture was stirred for 1 h at - 78 °C. The reaction mixture was then treated with MeI (4.0 mL, 63.8 mmol, 5.0 equiv.) and stirred for additional 4.5 h at -78 °C. The yellow reaction was quenched with AcOH (7.3 mL) and warmed up to room temperature. The yellow slurry obtained was then diluted with water (200 mL) and extracted with EtOAc (3 x 250 mL). The combined organic layers were dried over $MgSO_4$, filtered and then concentrated under reduced pressure. The residue was purified by flash column chromatography (SiO_2, 100 g, 55 mm ø, 0-40% EtOAc/iso-Hexanes, ca. 20 mL). This gave the product as a colourless oil (4.1 g, 98 %). ^{1}H NMR (400 MHz, $CDCl_3$) δ = 7.26 (ddt, m, 2H), 7.23 – 7.20 (m, 1H), 7.17 – 7.11 (m, 2H), 4.61 (ddt, J = 9.6, 7.3, 3.3 Hz, 1H), 4.18 – 4.04 (m, 2H), 3.73 – 3.58 (m, 1H), 3.20 (dd, J = 13.3, 3.3 Hz, 1H), 2.70 (dd, J = 13.3, 9.6 Hz, 1H), 2.12 – 2.00 (m, 2H), 1.70 (t, J = 2.5 Hz, 4H), 1.52 – 1.25 (m, 4H), 1.16 (d, J = 6.8 Hz, 3H); ^{13}C NMR (101 MHz, $CDCl_3$) δ = 177.7, 153.5, 135.8, 129.9, 129.4, 127.8, 79.5, 76.1, 66.5, 55.8, 38.4, 38.1, 33.3, 29.4, 26.9, 19.1, 17.8, 3.9; $[\alpha]_D^{20}$= − 62.2 (c = 1.03, $CHCl_3$); IR (film): $\tilde{\nu}$ = 3065, 3028, 2934, 2861, 1780, 1697, 1650, 1494, 1481, 1454, 1386, 1350, 1290, 1237, 1211, 1107, 1015, 972, 762, 747, 703, 522, 509, 411 cm^{-1}; HRMS-GC-EI (m/z): calc'd. for $C_{20}H_{25}NO_3$ $[M]^{+\cdot}$, 327.1828; found, 327.1830.

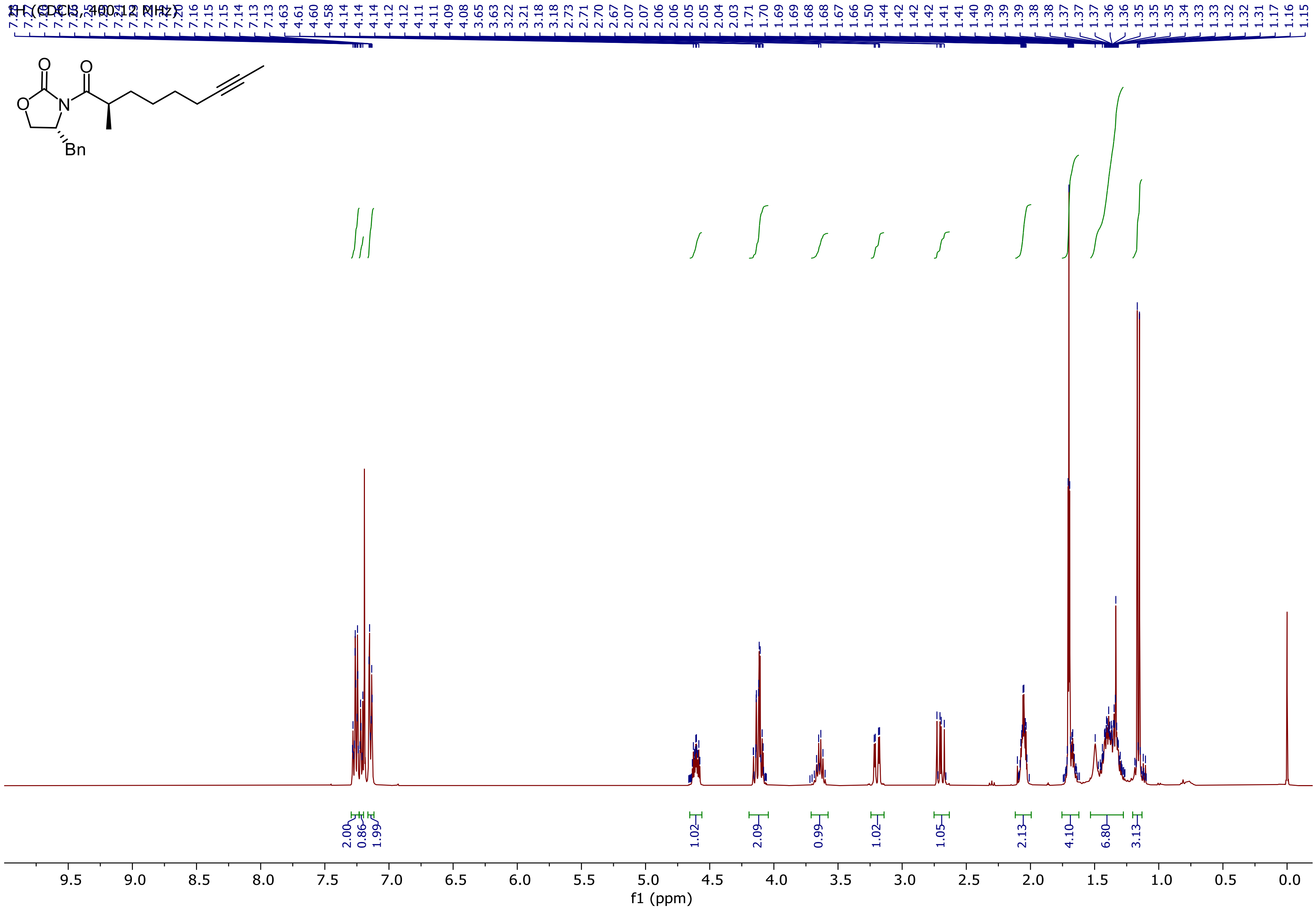
1H (CDCl3, 400.12 MHz)
Bn
f1 (ppm)

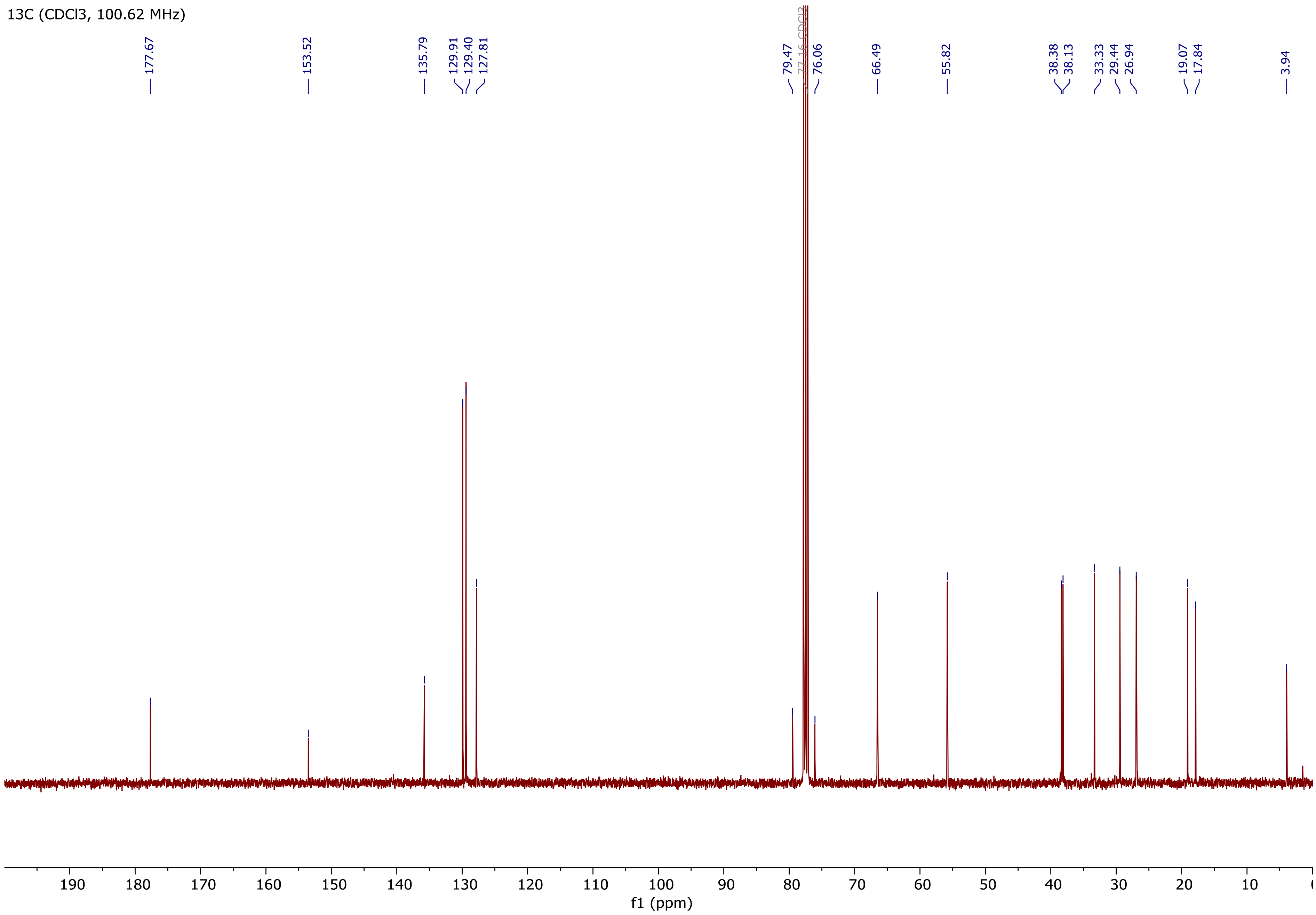
13C (CDCl3, 100.62 MHz)
177.67
153.52
135.79
129.91
129.40
127.81
79.47
77.16 CDCl3
76.06
66.49
55.82
38.38
38.13
33.33
29.44
26.94
19.07
17.84
3.94
f1 (ppm)

(2*R*,3*R*)-3-methyl-1-(4-tosyloxazol-5-yl)dec-8-yn-2-ol (7b)

(2*R*,3*R*)-3-methyl-1-(4-tosyloxazol-5-yl)dec-8-yn-2-ol **7b** was prepared by a five-step sequence starting from (*R*)-4-benzyl-3-((*R*)-2-methylnon-7-ynoyl)oxazolidin-2-one **11b** .

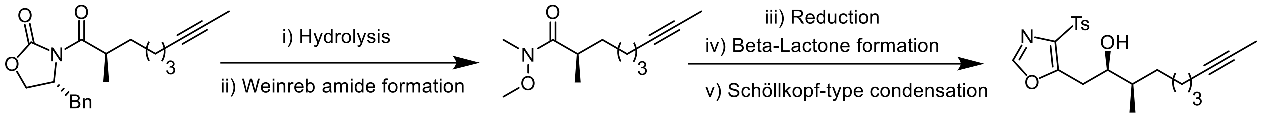

11b **13b** **7b**

Steps 1-2 (Hydrolysis and Amidation):

A round-bottom flask equipped with a stir bar, was charged with a solution of (*R*)-4-benzyl-3-((*R*)-2-methylnon-7-ynoyl)oxazolidin-2-one **11b** (4.2 g, 13.0 mmol) in THF-H_2O (45 mL, 1:1). The reaction mixture was cooled to 0 °C, before H_2O_2 (5.0 mL, 51.8 mmol, 4.0 equiv., 35 % w/w, aq.) and LiOH (621 mg, 25.9 mmol, 2.0 equiv.) were added. The resulting mixture was allowed to warm to rt and then stirred overnight. After that the THF was evaporated under reduced pressure and the aqueous fraction obtained was extracted with CH_2Cl_2 (3 x 50 mL). The aqueous solution was then acidified with dilute HCl (1 M ,aq) to pH 1 and re-extracted with CH_2Cl_2 (4 x 50 mL). The combined organic extracts were dried over $MgSO_4$, filtered and then concentrated under reduced pressure. This gave the product as a colourless oil (2.0 g, 91 %). ^{1}H NMR (400 MHz, $CDCl_3$): δ = 2.47 – 2.34 (m, 2H), 2.13 – 2.01 (m, 2H), 1.71 (t, *J* = 2.6 Hz, 3H), 1.69 – 1.55 (m, 1H), 1.48 – 1.29 (m, 4H), 1.12 (d, *J* = 7.0 Hz, 3H).

The optical purity of was determined (94 %ee) by HPLC on a crude sample of the acid **12b** using a chiral stationary phase (Chiralpak IC-3, 3 µm, 150 mm, ∅ 4.6 mm). The racemate of acid **12b** was prepared via three step sequence consisting of initial alkylation of diethyl methyl malonate with 7-iodohept-2-yne **S3**, followed by Krapcho dealkoxycarbonylation reaction and saponification.

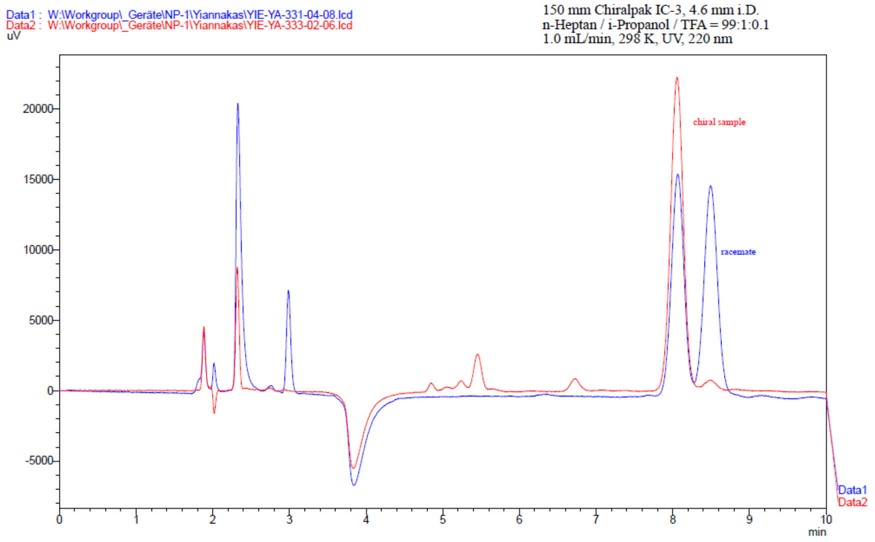

Valve R : 0

3.0 µL YIE-YA-331-04, Racemat
ca. 4.0 mg in 500 µL nC7/iProp/TFA=99:1:0.1
150 mm Chiralpak IC-3, 4.6 mm i.D.,
n-Heptan / i-Propanol / TFA = 99:1:0.1
(frische TFA!)
1.0 mL / min, 8.9 MPa, 298 K
UV, 220 nm

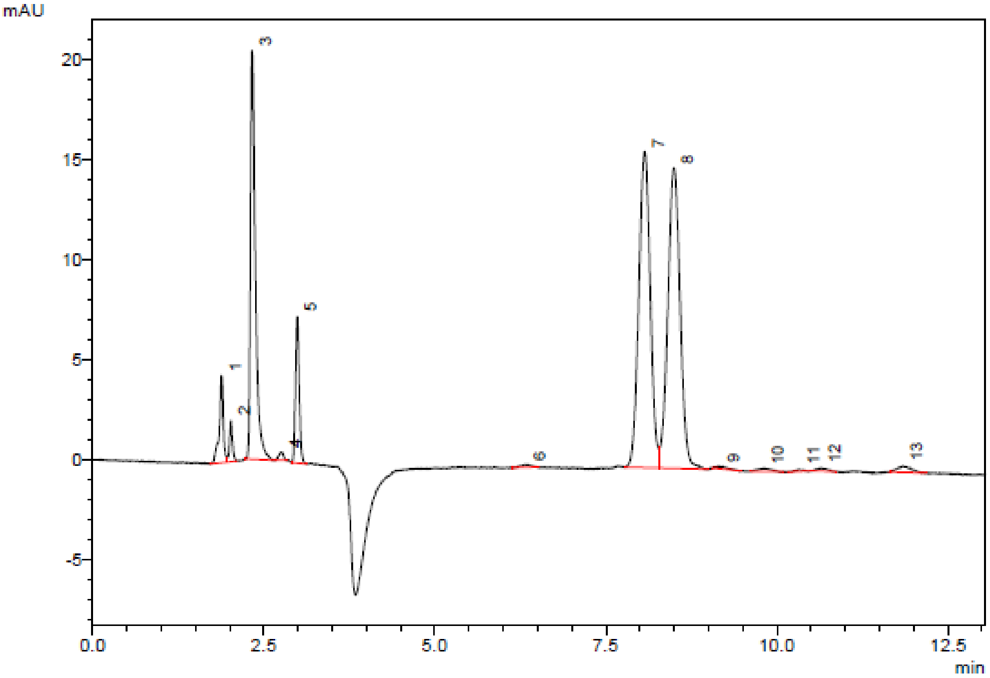

1 220nm,4nm

PDA Ch1 220nm

Peak #	Ret. Time	Area %	Name
1	1.88	3.28	
2	2.02	1.12	
3	2.33	19.04	
4	2.76	0.34	
5	2.99	5.35	
6	6.33	0.27	
7	8.07	34.05	1st enantiomer
8	8.49	34.75	2nd enantiomer
9	9.16	0.25	
10	9.81	0.37	
11	10.33	0.11	
12	10.65	0.28	
13	11.84	0.79	
Total		100.00	

3.0 µL YIE-YA-333-02, chirale Probe
ca. 4.0 mg in 500 µL nC7
150 mm Chiralpak IC-3, 4.6 mm i.D.,
n-Heptan / i-Propanol / TFA = 99:1:0.1
(frische TFA!)
1.0 mL / min, 7.4 MPa, 298 K
UV, 220 nm

Valve R : 0

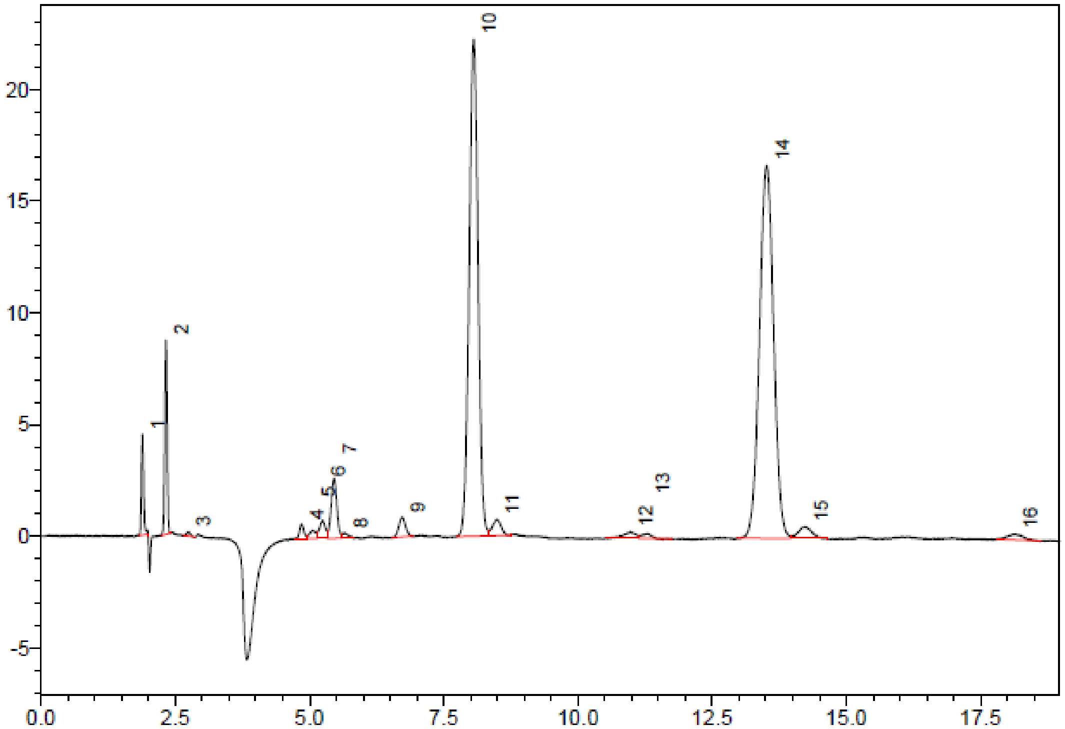

1 PDA Ch1 220nm

Peak #	Ret. Time	Area %	Name	
1	1.88	1.91		
2	2.32	3.93		
3	2.73	0.13		
4	4.85	0.54		
5	5.05	0.46		
6	5.24	0.85		
7	5.45	3.05		
8	5.66	0.23		
9	6.72	1.11		
10	8.06	38.29	1st enantiomer	ee = 94.0 %
11	8.49	1.18	2nd enantiomer	
12	10.98	0.69		
13	11.28	0.37		
14	13.51	45.11		
15	14.23	1.35		
16	18.13	0.81		
Total		100.00		

A two-neck round round-bottomed flask equipped with a magnetic stir bar, a glass stopper and a gas inlet connected to an argon-vacuum manifold, was charged with solution of the crude acid **12b** (1.6 g, 11.4 mmol) in CH_2Cl_2 (56 mL). Upon cooling to 0 °C, 1,1-carbonyldiimidazole (2.6 g, 15.5 mmol, 1.2 equiv.) was added and the resulting mixture was stirred at this temperature for 0.5 h. After that *N,O*-dimethylhydroxylamine-hydrochloride (3.2 g, 32.4 mmol, 2.5 equiv.) was added and the reaction mixture was allowed to rt overnight. The slurry obtained was then filtered and the filtrate was washed with dilute HCl (30 mL, 1 M, aq.). The organic phase was then dried over $MgSO_4$, filtered, and then concentrated under reduced pressure. This afforded the amide **13b** as a colourless oil (2.2 g, 82% over two steps). ^{1}H NMR (400 MHz, $CDCl_3$): δ = 3.69 (s, 3H), 3.19 (s, 3H), 2.16 – 2.06 (m, 2H), 1.77 (t, *J* = 2.5 Hz, 3H), 1.70 – 1.32 (m, 7H), 1.11 (d, *J* = 6.8 Hz, 3H); ^{13}C NMR (101 MHz, $CDCl_3$) δ = 178.6, 79.6, 75.9, 61.9, 35.5, 33.8, 32.7, 29.6, 27.3, 19.1, 17.9, 3.9; IR (film): $\tilde{\nu}$ = 2935, 2860, 1661, 1462, 1415, 1385, 1318, 1176, 1118, 1089, 995, 741, 711, 599, 526, 437 cm^{-1}; $[\alpha]_D^{20}$= − 17.2 (*c* = 1.14, $CHCl_3$); HRMS-ESI+ (*m/z*): calc'd. for $C_{12}H_{21}NO_2Na$ $[M+Na]^+$, 234.1464; found, 234.1464.

The optical purity of was determined (92.3 %ee) by HPLC on a crude sample of the amide **13b** using a chiral stationary phase (Chiralcel OD-3, 3 µm, 150 mm, ∅ 4.6 mm). Racemate of Weinreb amide **13b** was prepared by amidation of the racemate of acid **12b** .

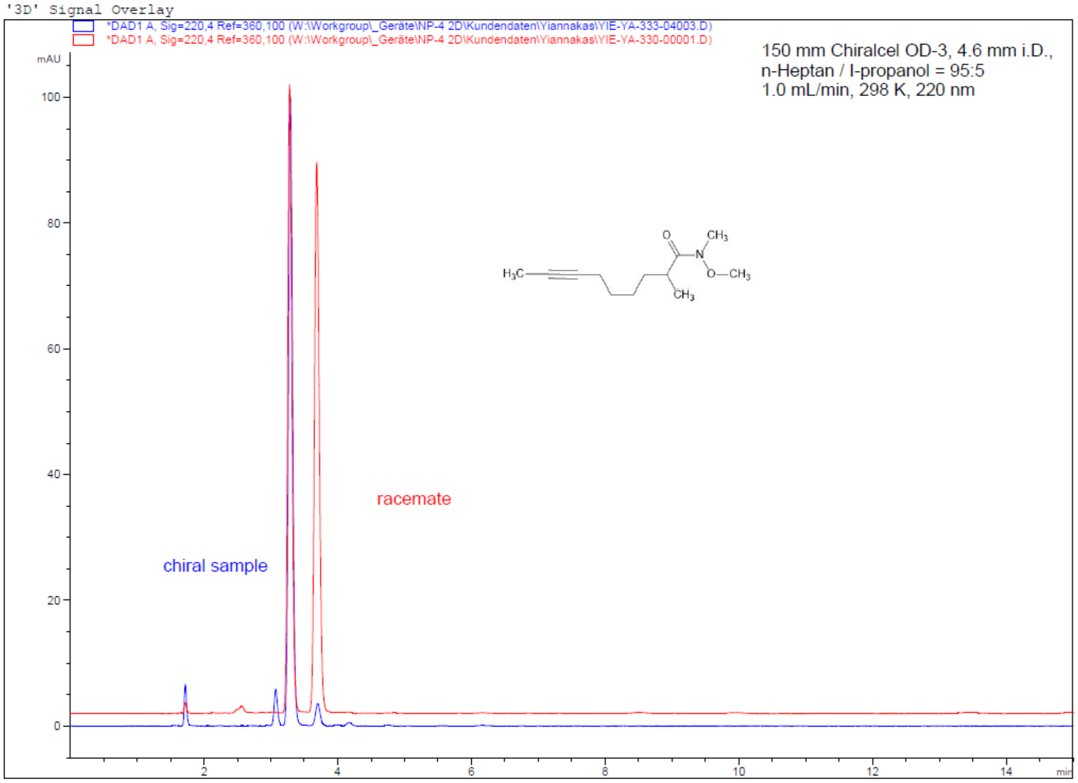

```
Sample Info      : 1.0 µL YIE-YA-330-00, Racemat (in i-Propanol)
                   150 mm Chiralcel OD-3, 4.6 mm i.D., Säule 1
                   n-Heptan/2-Propanol = 95:5
                   1.0 mL/min, 9.8 MPa, 298 K
                   UV, 220 nm
```

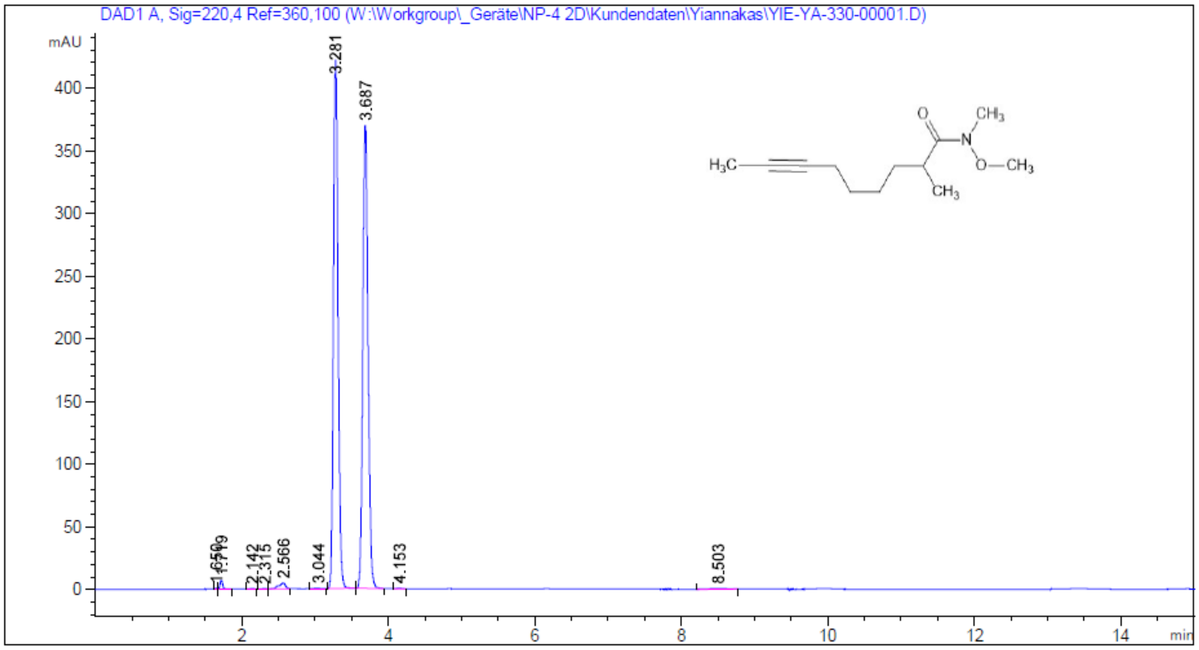

Signal 1: DAD1 A, Sig=220,4 Ref=360,100

Peak #	RetTime [min]	Type	Width [min]	Area [mAU*s]	Height [mAU]	Area %	
1	1.650	BV	0.0292	1.45365	8.00258e-1	0.0388	
2	1.719	VB	0.0400	18.16861	7.17691	0.4852	
3	2.142	BB	0.0471	1.33084	3.36794e-1	0.0355	
4	2.315	BB	0.0514	9.10512e-1	2.29083e-1	0.0243	
5	2.566	BB	0.0823	27.07807	4.58841	0.7231	
6	3.044	BB	0.0892	3.13082	4.23592e-1	0.0836	
7	3.281	BB	0.0678	1838.23206	421.79651	49.0881	1st enantiomer
8	3.687	BB	0.0777	1842.44434	369.44537	49.2006	2nd enantiomer
9	4.153	BB	0.0632	2.24327	4.78116e-1	0.0599	
10	8.503	BB	0.1626	9.77014	7.27873e-1	0.2609	

Totals : 3744.76231 806.00292

==

Sample Info : 1.0 µL YIE-YA-333-04, chirale Probe (in i-Propanol)
150 mm Chiralcel OD-3, 4.6 mm i.D., Säule 1
n-Heptan/2-Propanol = 95:5
1.0 mL/min, 9.8 MPa, 298 K
UV, 220 nm

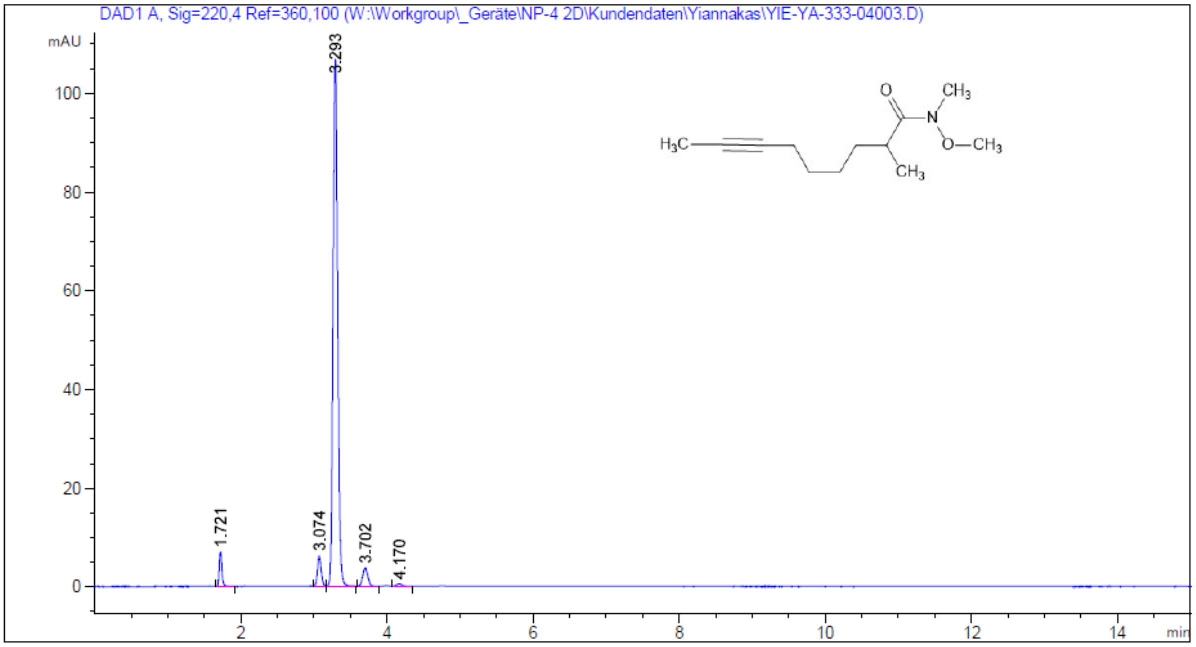

Signal 1: DAD1 A, Sig=220,4 Ref=360,100

Peak #	RetTime [min]	Type	Width [min]	Area [mAU*s]	Height [mAU]	Area %	
1	1.721	BB	0.0399	18.08478	7.05046	3.4503	
2	3.074	BV	0.0546	21.84524	6.20225	4.1678	
3	3.293	VB	0.0665	462.60187	106.86387	88.2584	1st enantiomer
4	3.702	BB	0.0767	18.49900	3.77140	3.5294	2nd enantiomer
5	4.170	BB	0.0721	3.11383	5.80327e-1	0.5941	

ee = 92.3 %

Totals : 524.14471 124.46831

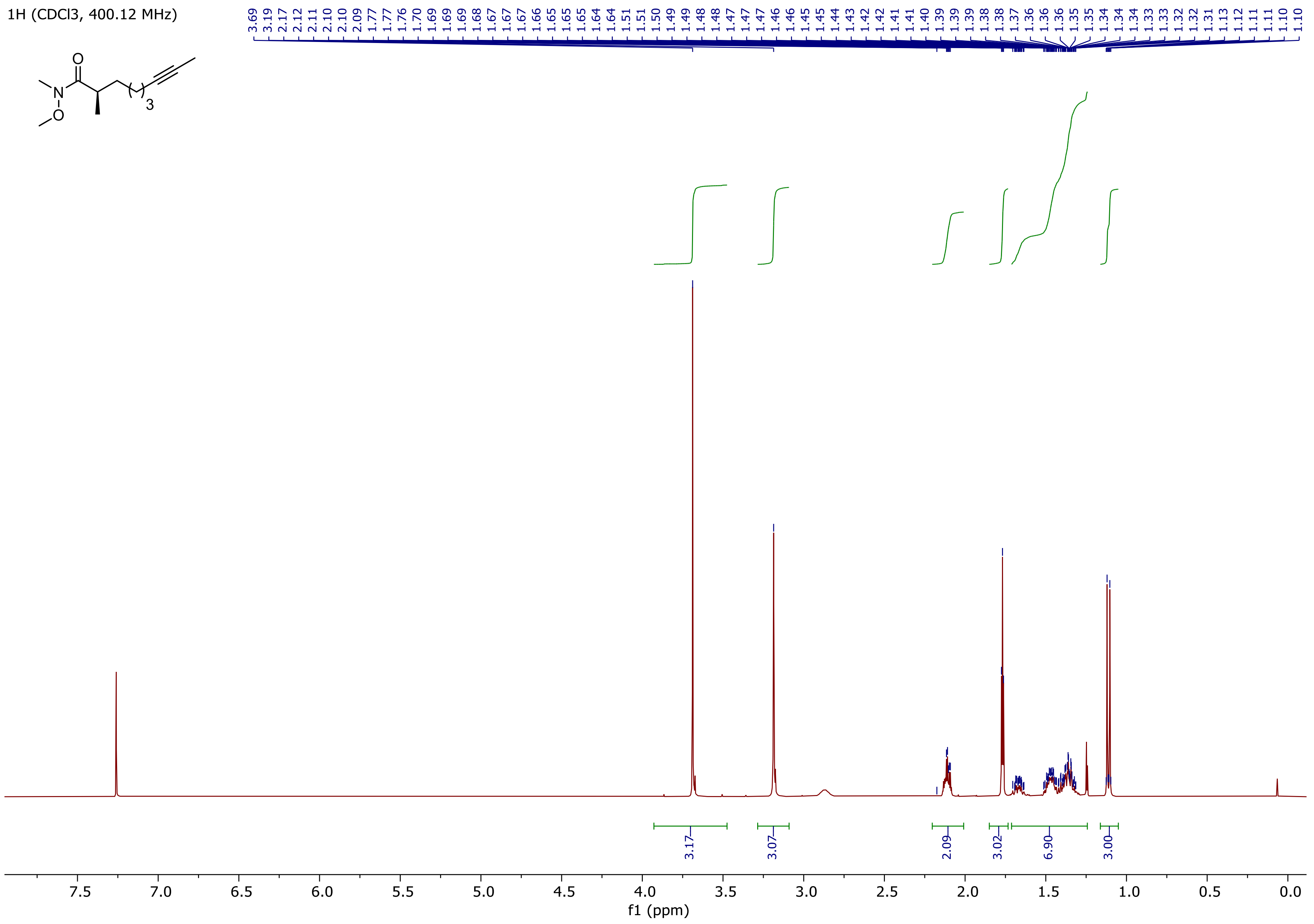
1H (CDCl3, 400.12 MHz)
3.69 3.19 2.17 2.12 2.11 2.10 2.10 2.09 1.77 1.77 1.76 1.70 1.69 1.69 1.69 1.68 1.67 1.67 1.67 1.66 1.65 1.65 1.65 1.64 1.64 1.51 1.51 1.50 1.49 1.49 1.48 1.48 1.47 1.47 1.47 1.46 1.46 1.45 1.45 1.44 1.43 1.42 1.42 1.41 1.41 1.40 1.39 1.39 1.39 1.38 1.38 1.37 1.36 1.36 1.36 1.35 1.35 1.34 1.34 1.34 1.33 1.33 1.32 1.32 1.31 1.13 1.12 1.11 1.11 1.10 1.10
3.17
3.07
2.09
3.02
6.90
3.00
7.5 7.0 6.5 6.0 5.5 5.0 4.5 4.0 3.5 3.0 2.5 2.0 1.5 1.0 0.5 0.0
f1 (ppm)

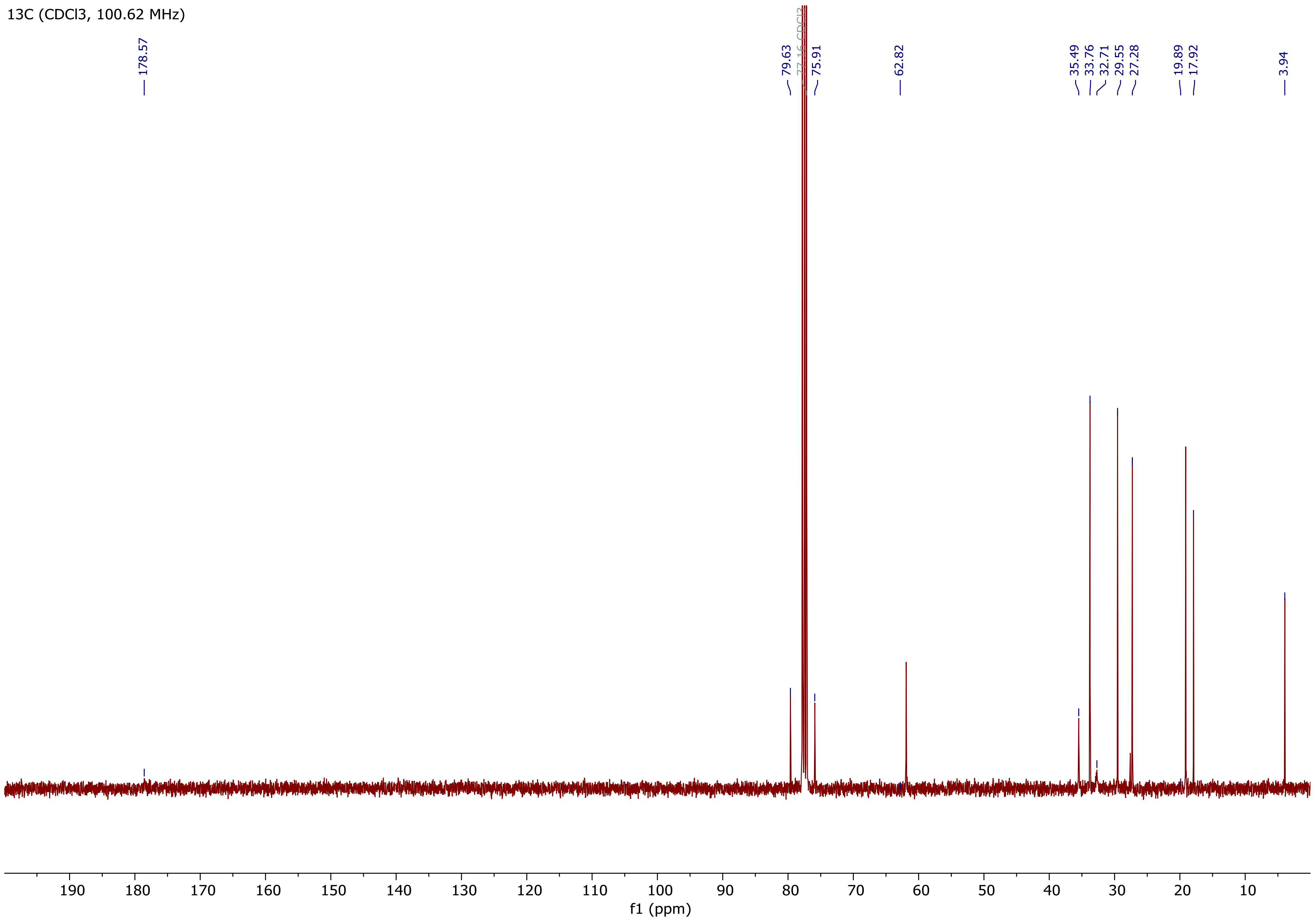
13C (CDCl3, 100.62 MHz)
178.57
79.63
77.16 CDCl3
75.91
62.82
35.49
33.76
32.71
29.55
27.28
19.89
17.92
3.94
190
180
170
160
150
140
130
120
110
100
90
80
70
60
50
40
30
20
10
f1 (ppm)

Steps 3-4 (Weinreb Amide Reduction & Cyclocondesation):

A three-neck jacketed vessel equipped with a stir bar, a gas inlet connected to an argon-vacuum manifold, a ground-glass joint thermometer adapter connected to low temperature thermometer and a rubber septum was charged with a solution of the crude amide **13b** (2.2 g, 10.6 mmol) in CH_2Cl_2 (60 mL). The reaction mixture was cooled to –78 °C and DIBAL-H (10.6 mL, 10.6 mmol, 1.0 equiv., 1.0 M in *n*-hexane) was added dropwise. The resulting mixture stirred for 1 h at this temperature, before it was quenched with Rochelle salt (50 mL, sat.aq.). The slurry immediately formed was allowed to warm to rt. The biphasic mixture obtained was then separated and the aqueous layer was extracted with CH_2Cl_2 (3 x 50 mL). The combined organic extracts were dried over $MgSO_4$, filtered, and then concentrated under reduced pressure (300m mbar) at rt. The crude aldehyde **9b** was engaged to the next without any purification. A three-neck jacketed vessel equipped with a stir bar, a gas inlet connected to an argon-vacuum manifold, a ground-glass joint thermometer adapter connected to low temperature thermometer and a rubber septum was charged with a solution of lithium perchlorate (1.2 g, 10.6 mmol, 1.0 equiv.) in Et_2O (17 mL) and a solution of TMS*q* **14** (422 mg, 1.1 mmol, 10 mol%) in CH_2Cl_2 (35 mL). The mixture was stirred for 0.5 h at rt and then cooled to –78 °C. DIPEA (4.7 mL, 27.0 mmol, 2.25 equiv.) was added, followed by a solution of the crude aldehyde **9b** in CH_2Cl_2 -Et_2O (51 mL, 2:1). A solution of AcCl (1.14 mL, 16.0 mmol, 1.3 equiv.) in CH_2Cl_2 (9 mL) was added via syringe pump over 5 h and the resulting mixture was stirred at –78 °C for 24 h. The reaction mixture was diluted with Et_2O (50 mL) and then allowed to warm to rt. The yellow suspension obtained was then filtered through a silica plug eluting with Et_2O (100 mL) and the filtrate obtained was concentrated under reduced pressure (300 mbar) at rt. This afforded the crude beta-lactone **8b** as a pale yellow oil (> 99:1 dr), which was subjected to the next step without any purification.

Step 5 (Schöllkopf Condensation):

A three-neck jacketed vessel equipped with a stir bar, a gas inlet connected to an argon-vacuum manifold, a ground-glass joint thermometer adapter connected to low temperature thermometer and a rubber septum, was charged with a solution of TosMIC (2.0 g, 10.1 mmol, 1.0 equiv.) in THF (41 mL). The solution was cooled to –78 °C and ^{n}BuLi (6.6 mL, 12.0 mmol, 1.0 equiv., 1.6 M in *n*-hexanes) was added dropwise. The pale yellow solution obtained was stirred at –78 °C for 0.5 h, before a solution of the crude beta-lactone **8b** in THF (20 mL) was added. The resulting mixture was stirred at –78 °C for an hour and then warmed to rt. The reaction was then quenched with HCl (20 mL, 1.0 M, aq.) and extracted with EtOAc (3 x 100 mL). The combined organic extracts were dried over $MgSO_4$, filtered and then concentrated under reduced pressure. The dark brown oily residue obtianed was purified by flash column chromatography (SiO_2, 100 g, 55 mm ø, 0-100% EtOAc/iso-Hexanes, ca. 20 mL). This gave the product as an orange viscous oil (2.2 g, 43 % yield over 5 steps, > 99:1 dr).

(2*R*,3*R*)-3-methyl-1-(4-tosyloxazol-5-yl)dec-8-yn-2-ol **7b** was also prepared by a three-step sequence starting from (*R*)-*N*-((1S,2S)-1-hydroxy-1-phenylpropan-2-yl)-*N*,2-dimethylnon-7-ynamide **18b**.

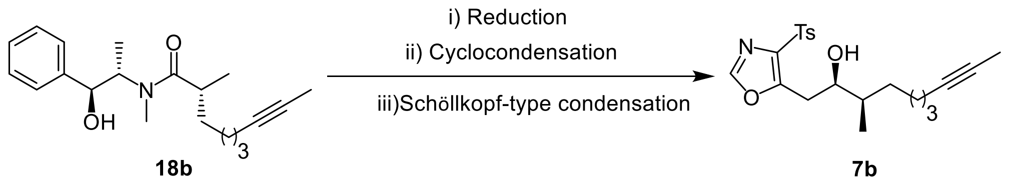

Reduction & Cyclocondensation:

A three-neck jacketed vessel equipped with a stir bar, a gas inlet connected to an argon-vacuum manifold, a ground-glass joint thermometer adapter connected to low temperature thermometer and a rubber septum was charged with $LiAlH_4$ (1.5 g, 40.6 mmol, 2.3 equiv.) and iso-hexane (96 mL). The vessel was cooled to 0 °C and ethyl acetate (6.0 mL, 60.3 mmol, 3.4 equiv) was added via a syringe pump over a period of 1.5 h. The resulting suspension of lithium triethoxyaluminum hydride was cooled to −78 °C. A solution of amide **18b** (5.6 g, 17.7 mmol, 1.0 equiv) in THF (64.5 mL) was added via a syringe pump over 5 min, and the reaction mixture was warmed to 0 °C. After being stirred for 1 h at 0 °C, the reaction mixture was transferred by cannula to an ice-cold solution of trifluoroacetic acid (13.5 mL, 177 mmol, 10 equiv) in dilute HCl (80 mL, 1.0 M, aq).The resulting biphasic mixture was diluted with dilute HCl (140 mL, 1.0 M, aq.) and the layers were separated. The aqueous layer was extracted with CH_2Cl_2 (3 × 100 mL). The combined organic layers were neutralized with $NaHCO_3$ (100 mL, sat., aq.). The aqueous layer (pH 7−8) was separated and extracted with CH_2Cl_2 (100 mL). The combined organic extracts were dried over $MgSO_4$, filtered and concentrated under reduced pressure (300 mbar) at rt. This gave the crude aldehyde **9b** as pale yellow oil. Due to concerns about potential oxidation and racemization, the crude aldehyde **9b** was subjected to the next step without any purification. A three-neck jacketed vessel equipped with a stir bar, a gas inlet connected to an argon-vacuum manifold, a ground-glass joint thermometer adapter connected to low temperature thermometer and a rubber septum was charged with a solution of lithium perchlorate (1.9 g, 17.7 mmol, 1.0 equiv.) in Et_2O (28 mL) and a solution of TMS*q* (702 mg, 1.8 mmol, 10 mol%) in CH_2Cl_2 (56 mL). The mixture was stirred for 0.5 h at rt and then cooled to –78 °C. DIPEA (7.7 mL, 44.2 mmol, 2.5 equiv.) was added, followed by a solution of the crude aldehyde **9b** in CH_2Cl_2-Et_2O (42 mL, 2:1). A solution of AcCl (2.3 mL, 32.5 mmol, 1.3 equiv.) in CH_2Cl_2 (15 mL) was added via syringe pump over 5 h and the resulting mixture was stirred at –78 °C for 24 h. The reaction mixture was diluted with Et_2O (100 mL) and then allowed to warm to rt. The yellow suspension obtained was then filtered through a silica plug eluting with Et_2O (100 mL) and the filtrate obtained was concentrated under reduced pressure (300 mbar) at rt. This afforded the crude beta-lactone **8b** as a pale yellow oil (> 99:1 dr by NMR), which was subjected to the next step without any purification.

Schöllkopf Condesation:

A three-neck jacketed vessel equipped with a stir bar, a gas inlet connected to an argon-vacuum manifold, a ground-glass joint thermometer adapter connected to low temperature thermometer and a rubber septum, was charged with a solution of TosMIC (2.5 g, 12.3 mmol, 1.0 equiv.) in THF (42 mL). The solution was cooled to –78 °C and ^{n}BuLi (8.1 mL, 12.9 mmol, 1.1 equiv., 1.6 M in *n*-hexanes) was added dropwise. The pale yellow solution obtained was stirred at –78 °C for 0.5 h, before a solution of the crude beta-lactone **8b** in THF (20 mL) was added. The resulting mixture was stirred at –78 °C for an hour and then warmed to rt. The reaction was then quenched with HCl (20 mL, 1.0 M, aq.) and extracted with EtOAc (3 x 100 mL). The combined organic extracts were dried over $MgSO_4$, filtered and then concentrated under reduced pressure. The dark brown oily residue obtianed was purified by flash column chromatography (SiO_2, 100 g, 55 mm ø, 0-100% EtOAc/iso-Hexanes, ca. 20 mL). This gave the product as an orange viscous oil (1.7 g, 24 % yield over 3 steps, > 99:1 dr). The spectroscopic data are in agreement with those obtained previously for (2*R*,3*R*)-3-methyl-1-(4-tosyloxazol-5-yl)dec-8-yn-2-ol **7b**. ^{1}H NMR (400 MHz, $CDCl_3$): δ = 8.00 – 7.86 (m, 2H), 7.75 (s, 1H), 7.40 – 7.28 (m, 2H), 3.91 (dq, *J* = 9.8, 4.6 Hz, 1H), 3.37 – 3.24 (m, 1H), 3.18 (dd, *J* = 9.8, 3.5 Hz, 1H), 2.43 (s, 3H), 2.15 (ddt, *J* = 6.9, 4.6, 2.6 Hz, 2H), 1.88 (d, *J* = 6.4 Hz, 1H), 1.78 (t, *J* = 2.6 Hz, 3H), 1.53 – 1.17 (m, 7H), 1.01 (d, *J* = 6.9 Hz, 3H); ^{13}C NMR (101 MHz, $CDCl_3$): δ = 155.5, 149.5, 146.4, 137.9, 136.2, 130.4, 128.6, 79.6, 76.1, 74.2, 40.8, 33.5, 31.5, 28.5, 25.6, 22.7, 19.1, 14.1, 3.9; $[\alpha]_D^{20}$= + 24.7 (c = 2.84, $CHCl_3$); IR (film): $\tilde{\nu}$ = 3527, 3129, 2932,2859, 1594, 1517, 1494, 1460, 1402, 1381, 1323, 1305, 1244, 1216, 1146, 1085, 1060, 1017, 932, 860, 814, 754, 706, 696, 661, 600, 439, 494 cm^{-1}; HRMS-ESI+ (*m/z*): calc'd. for $C_{19}H_{23}N_1O_4SNa$ $[M+Na]^+$, 384.1240; found, 384.1241.

The diastereomeric purity of (2*R*,3*R*)-3-methyl-1-(4-tosyloxazol-5-yl)dec-8-yn-2-ol **7b** originally determined by NMR was confirmed by HPLC using a collection of chiral stationary phases.

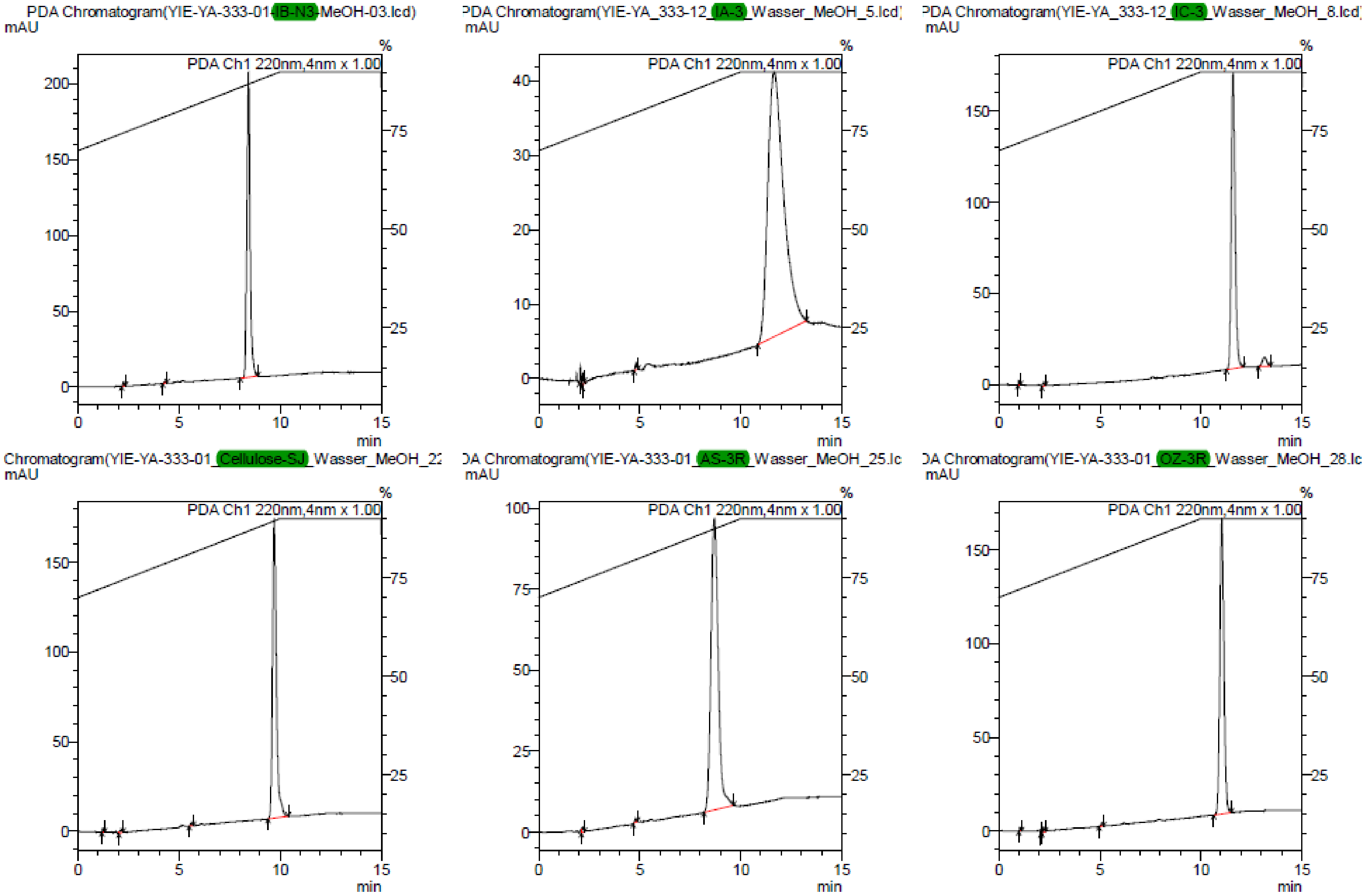

Methanol / water Gradient: 70 % - 10' - 90 % MeOH

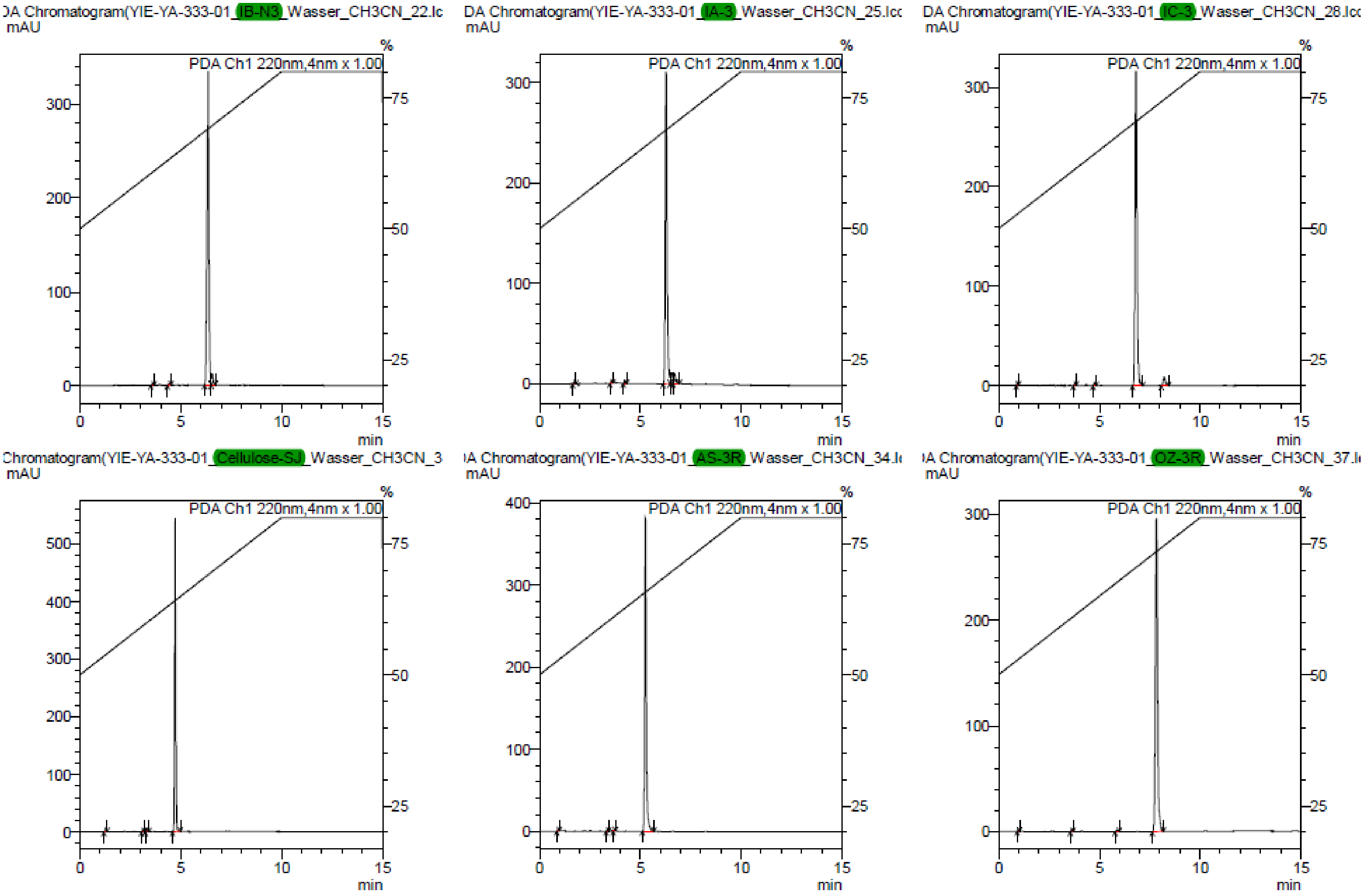

acetonitrile / water gradient: 50 % - 10 ' - 80 % ACN

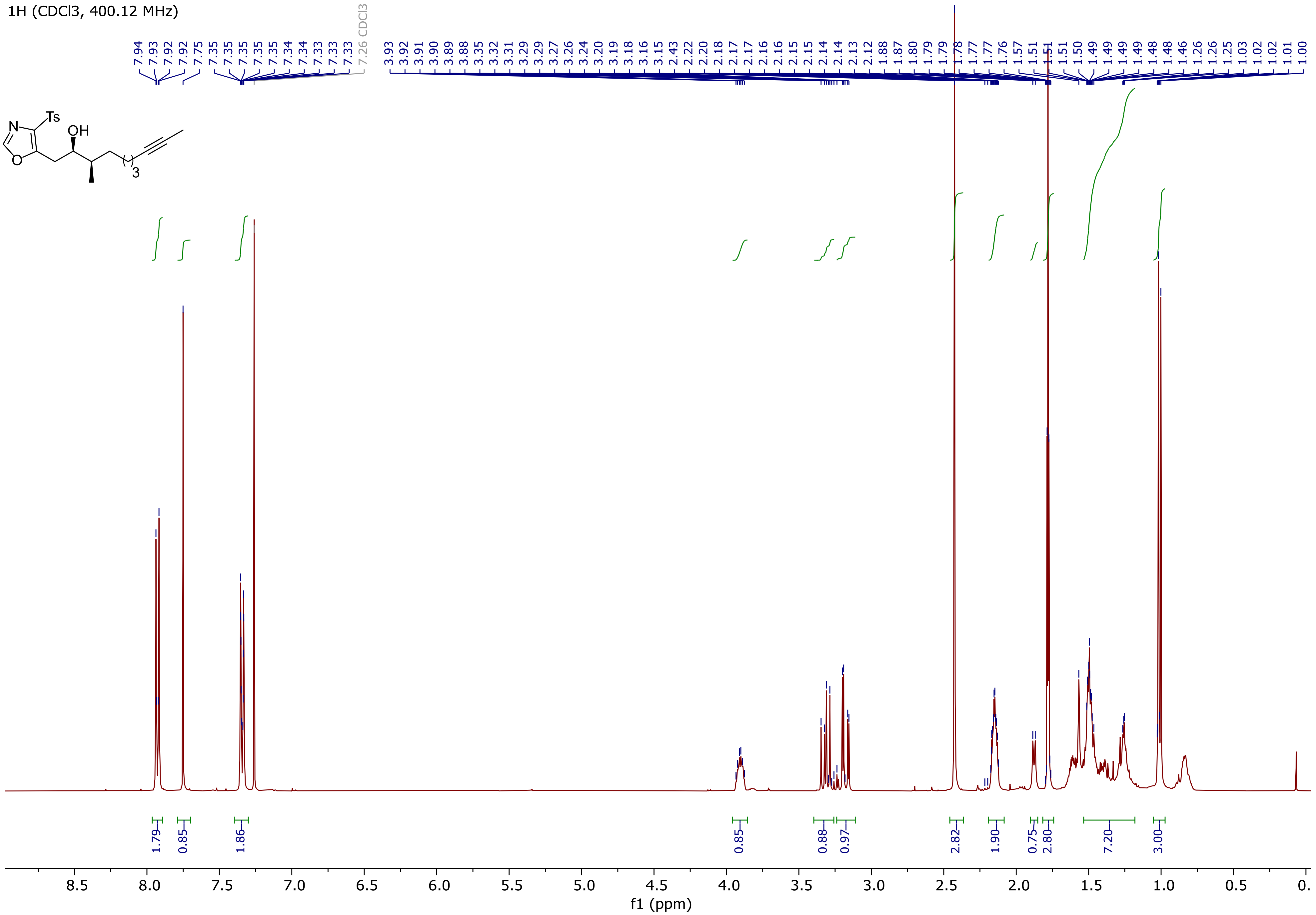
1H (CDCl3, 400.12 MHz)
7.26 CDCl3
f1 (ppm)

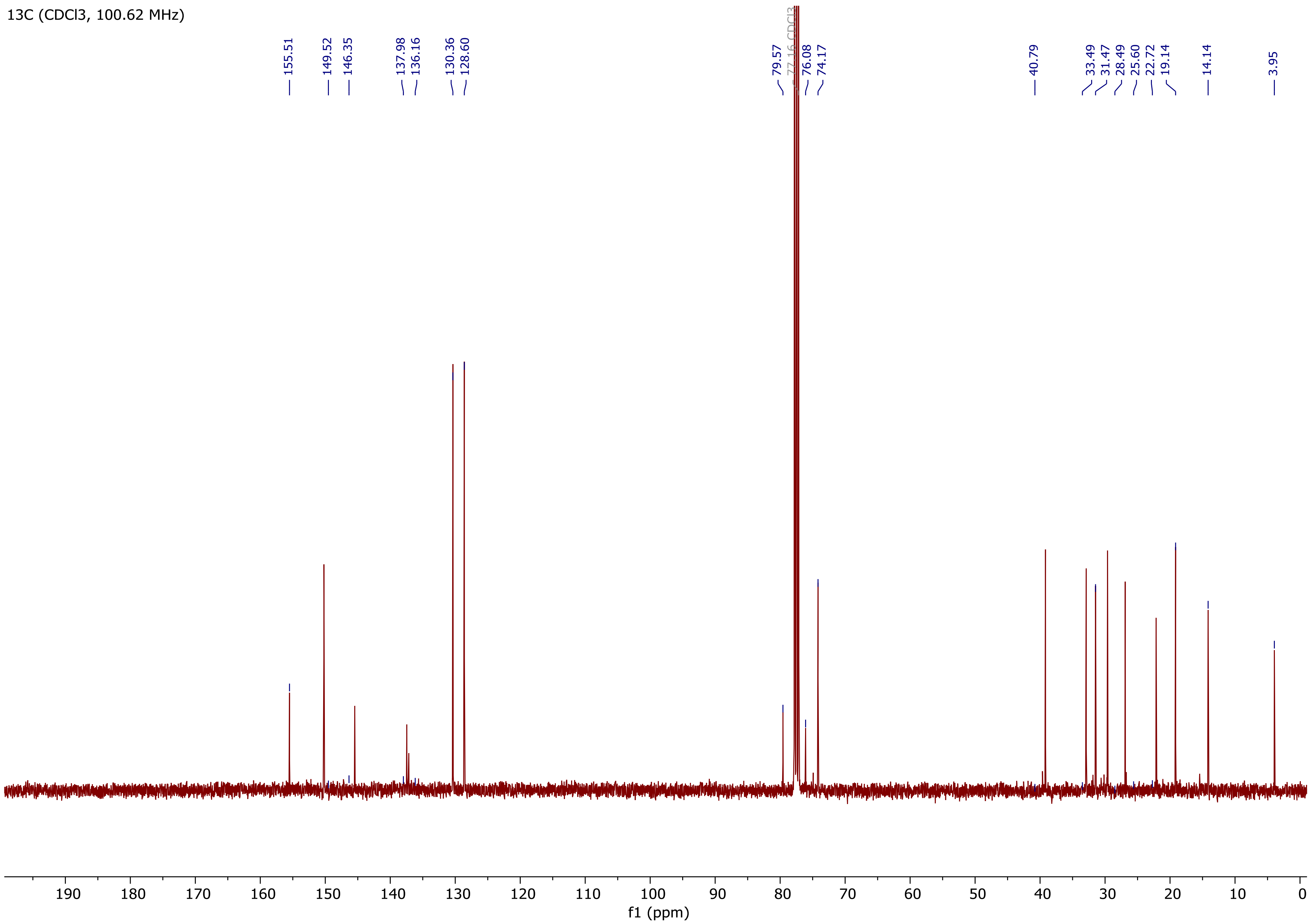
13C (CDCl3, 100.62 MHz)
155.51
149.52
146.35
137.98
136.16
130.36
128.60
79.57
77.16 CDCl3
76.08
74.17
40.79
33.49
31.47
28.49
25.60
22.72
19.14
14.14
3.95
190
180
170
160
150
140
130
120
110
100
90
80
70
60
50
40
30
20
10
0
f1 (ppm)

Mosher esters from (2*R*,3*R*)-3-methyl-1-(4-tosyloxazol-5-yl)dec-8-yn-2-ol **7b** and Mosher ester analysis.

(2*R*,3*R*)-3-methyl-1-(4-tosyloxazol-5-yl)dec-8-yn-2-yl (R)-3,3,3-trifluoro-2-methoxy-2-phenyl propanoate (15b)

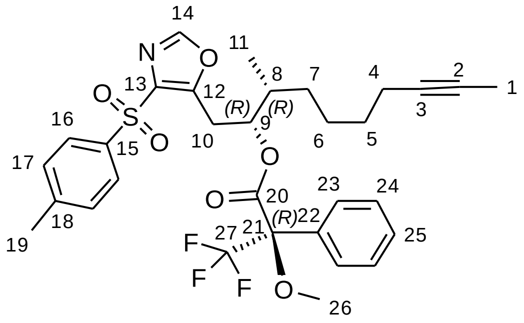

A Schleck tube equipped a magnetic stir bar was charged a solution of (2*R*,3*R*)-3-methyl-1-(4-tosyloxazol-5-yl)dec-8-yn-2-ol **7b** (39.0 mg, 97.2 µmol, 1.5 equiv.) in CH_2Cl_2 (1 mL), DMAP (1.0 mg, 6.3 µmol, 10.0 mol%) and TEA (20 µL, 195 µmol, 3.0 equiv.). (S)-(+)-MTPACl (12.0 µL, 65 µmol) was added and the resulting mixture was stirred overnight at rt. After that the solvent was evaporated under reduced pressure. The residue was purified by flash column chromatography (SiO_2, 10 g, 20 mm ø, 0-50% EtOAc/iso-Hexanes, ca. 5 mL). This gave the product as a pale yellow oil (33 mg, 85 %).^{1}H NMR (600 MHz, $CDCl_3$): δ = 7.94 – 7.89 (m, 2H), 7.47 (s, 1H), 7.39 – 7.33 (m, 5H), 7.33 – 7.28 (m, 2H), 5.48 (ddd, *J* = 9.2, 4.3, 3.4 Hz, 1H), 3.47 (dd, *J* = 15.3, 4.3 Hz, 1H), 3.45 (q, *J* = 1.2 Hz, 3H), 3.32 (dd, *J* = 15.3, 9.2 Hz, 1H), 2.43 (s, 3H), 2.12 (m, 2H), 1.86 – 1.79 (m, 1H), 1.77 (t, *J* = 2.6 Hz, 3H), 1.50 – 1.38 (m, 4H), 1.38 – 1.32 (m, 1H), 1.18 (dddd, *J* = 15.3, 10.2, 6.9 Hz, 1H), 1.04 (d, *J* = 6.9 Hz, 3H); ^{13}C NMR (151 MHz, $CDCl_3$): δ = 166.0, 152.6, 149.9, 145.1, 136.8, 136.7, 131.7, 129.9, 129.5, 128.5, 128.2, 127.2, 126.0 – 120.3 (q, *J* = 289.0 Hz) , 124.1, 122.2, 120.3, 84.8– 84.2 (q, *J* = 28.0 Hz) 78.9, 77.5, 75.7, 55.3, 36.7, 31.8, 29.0, 27.2, 26.4, 21.7, 18.6, 14.4, 3.4; IR (film): $\tilde{\nu}$ = 3134, 2937, 2859, 1746, 1595, 1515, 1495, 1451, 1386, 1329, 1292, 1252, 1169, 1149, 1121, 1084, 1018, 996, 932, 814, 766, 719, 697, 662, 601, 540, 505, 411 cm^{-1}; $[a]_D^{20}$= + 15.4 (c = 1.14, $CHCl_3$); HRMS-ESI+ (*m/z*): calc'd. for $C_{31}H_{34}F_3NO_6SNa$ $[M+Na]^+$, 628.1951; found, 628.1956.

(2*R*,3*R*)-3-methyl-1-(4-tosyloxazol-5-yl)dec-8-yn-2-yl (*S*)-3,3,3-trifluoro-2-methoxy-2-phenyl propanoate (16b)

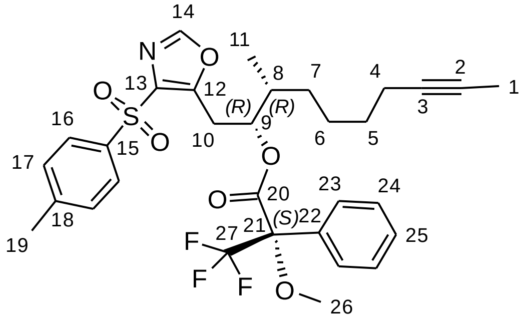

A Schleck tube equipped a magnetic stir bar was charged a solution of 2*R*,3*R*)-3-methyl-1-(4-tosyloxazol-5-yl)dec-8-yn-2-ol **7b** (39.0 mg, 97.2 µmol, 1.5 equiv.) in CH_2Cl_2 (1 mL), DMAP (1.0 mg, 6.3 µmol, 10.0 mol%) and TEA (20 µL, 195 µmol, 3.0 equiv.). (*R*)-(-)-MTPACl (12.0 µL, 65 µmol) was added and the resulting mixture was stirred overnight at rt. After that the solvent was evaporated under reduced pressure. The residue was purified by flash column chromatography (SiO_2, 10 g, 20 mm ø, 0-50% EtOAc/iso-Hexanes, ca. 5 mL). This gave the product as a pale yellow oil (34 mg, 88 %).^{1}H NMR (600 MHz, $CDCl_3$): δ = 7.93 – 7.89 (m, 2H), 7.65 (s, 1H), 7.37 – 7.27 (m, 7H), 5.50 (ddd, *J* = 8.6, 4.9, 3.3 Hz, 1H), 3.48 (dd, *J* = 15.5, 4.9 Hz, 1H), 3.44 (dd, *J* = 15.5, 8.6 Hz, 1H), 3.44 – 3.41 (m, 3H), 2.41 (s, 3H), 2.11 – 2.06 (m, 2H), 1.77 (t, *J* = 2.6 Hz, 3H), 1.37 (qd, *J* = 6.9, 4.9, 2.6 Hz, 2H), 1.33 – 1.24 (m, 2H), 1.14 – 1.06 (m, 1H), 1.00 (d, *J* = 6.9 Hz, 3H); ^{13}C NMR (151 MHz, $CDCl_3$): δ = 165.9, 152.7, 149.9, 145.1, 136.9, 136.7, 131.9, 129.9, 129.9, 129.6, 128.4, 128.2, 127.1, 126.1 – 121.4 (q, *J* = 289.0 Hz) , 124.2, 122.3, 120.4, 84.7– 84.2 (q, *J* = 28.0 Hz) 78.9, 77.3, 75.7, 55.3, 36.6, 31.6, 28.9, 27.4, 26.4, 21.7, 18.6, 14.2, 3.4; IR (film): $\tilde{\nu}$ = 3134, 2937, 2859, 1746, 1595, 1515, 1495, 1451, 1386, 1329, 1292, 1252, 1169, 1149, 1121, 1084, 1018, 996, 932, 814, 766, 719, 697, 662, 601, 540, 505, 487 cm^{-1}; $[a]_D^{20}$= - 4.5 (c = 1.10, $CHCl_3$); HRMS-ESI+ (*m/z*): calc'd. for $C_{31}H_{34}F_3NO_6SNa$ $[M+Na]^+$, 628.1951; found, 628.1956.

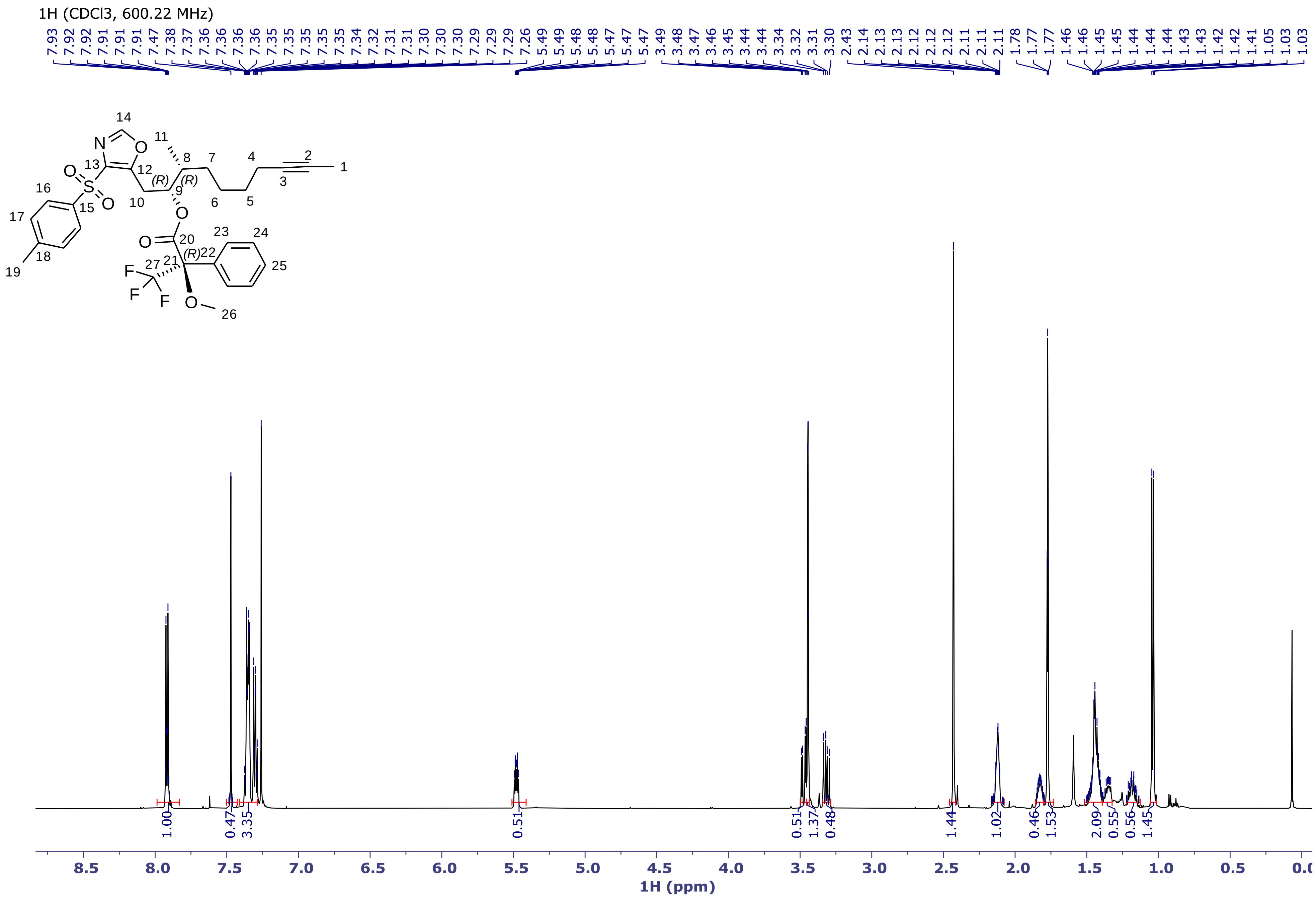
1H (CDCl3, 600.22 MHz)
7.93 7.92 7.92 7.91 7.91 7.91 7.47 7.38 7.37 7.36 7.36 7.36 7.36 7.35 7.35 7.35 7.35 7.35 7.34 7.32 7.31 7.31 7.30 7.30 7.30 7.29 7.29 7.29 7.26 5.49 5.49 5.48 5.48 5.47 5.47 5.47 3.49 3.48 3.47 3.46 3.45 3.44 3.44 3.34 3.32 3.31 3.30 2.43 2.14 2.13 2.13 2.12 2.12 2.12 2.11 2.11 2.11 1.78 1.77 1.77 1.46 1.46 1.45 1.45 1.44 1.44 1.44 1.43 1.43 1.42 1.42 1.41 1.05 1.03 1.03
1.00 0.47 3.35 0.51 0.51 1.37 0.48 1.44 1.02 0.46 1.53 2.09 0.55 0.56 1.45
8.5 8.0 7.5 7.0 6.5 6.0 5.5 5.0 4.5 4.0 3.5 3.0 2.5 2.0 1.5 1.0 0.5
1H (ppm)

13C (CDCl3, 150.94 MHz)

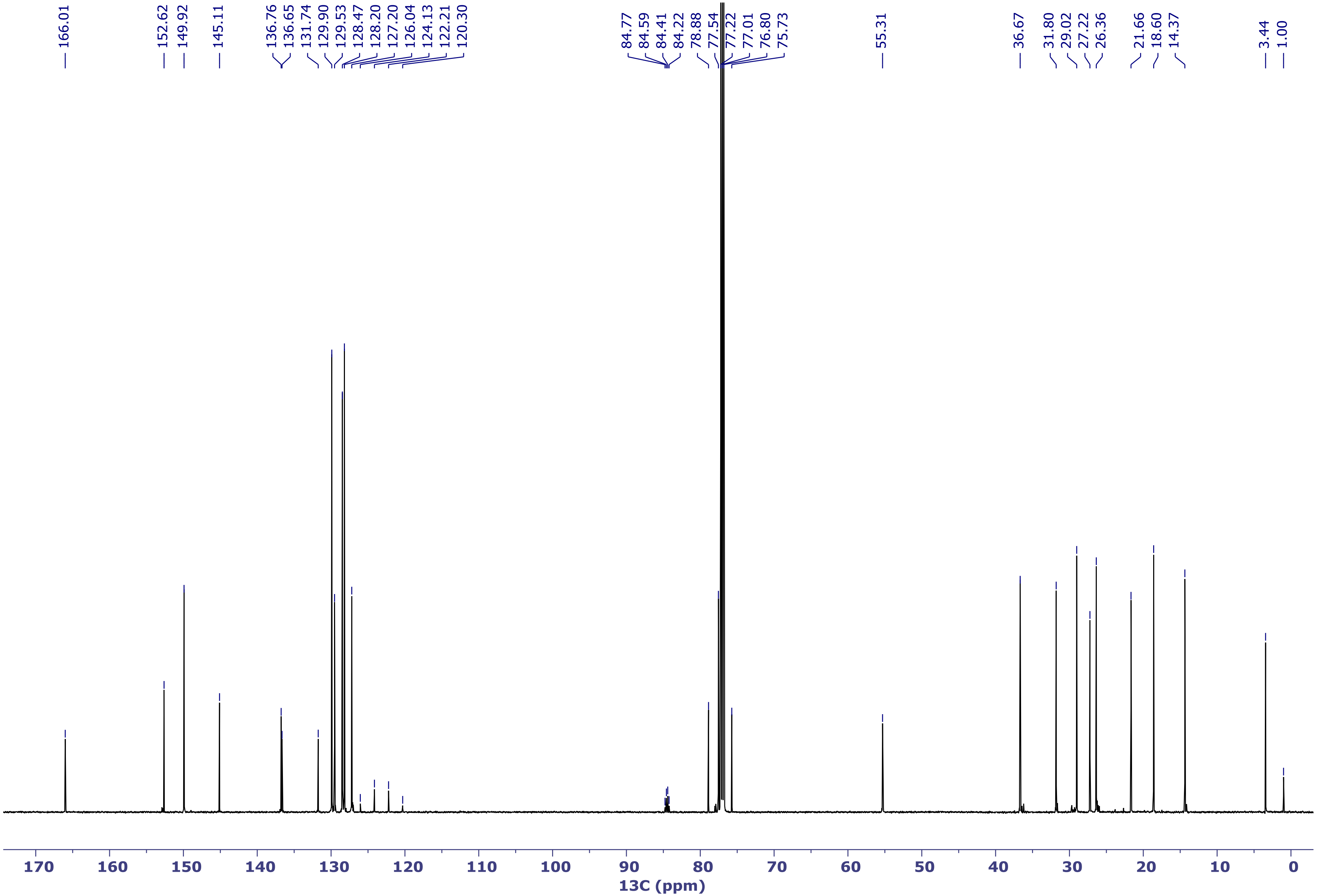

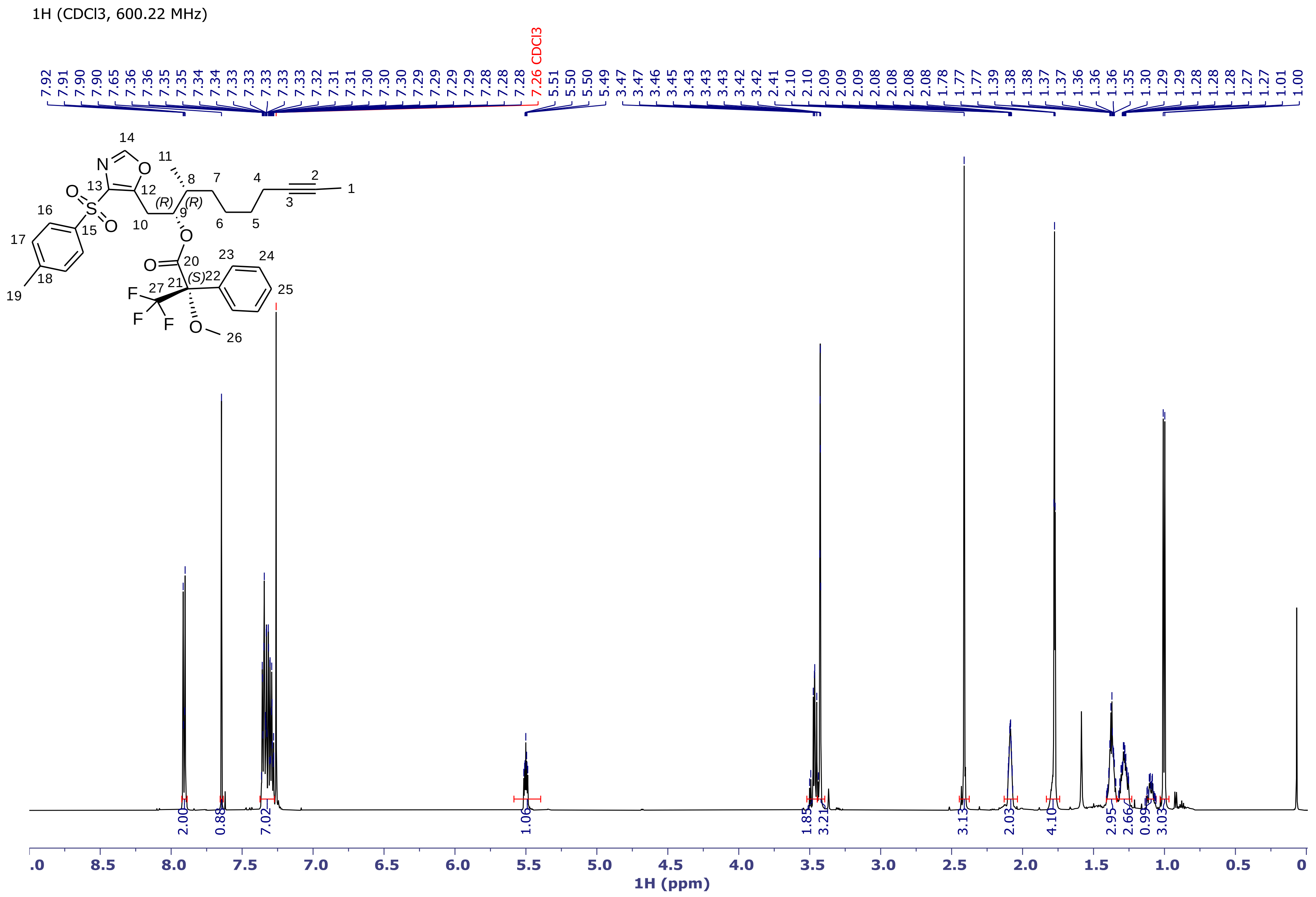
1H (CDCl3, 600.22 MHz)
7.92 7.91 7.90 7.90 7.65 7.36 7.36 7.35 7.35 7.34 7.34 7.33 7.33 7.33 7.33 7.33 7.32 7.31 7.31 7.30 7.30 7.30 7.29 7.29 7.29 7.29 7.28 7.28 7.28
7.26 CDCl3
5.51 5.50 5.50 5.49 3.47 3.47 3.46 3.45 3.43 3.43 3.43 3.42 3.42 2.41 2.10 2.10 2.09 2.09 2.09 2.08 2.08 2.08 1.78 1.77 1.77 1.39 1.38 1.38 1.37 1.37 1.36 1.36 1.36 1.35 1.30 1.29 1.29 1.28 1.28 1.28 1.27 1.27 1.01 1.00
2.00 0.88 7.02 1.06 1.85 3.21 3.13 2.03 4.10 2.95 2.66 0.99 3.03
1H (ppm)

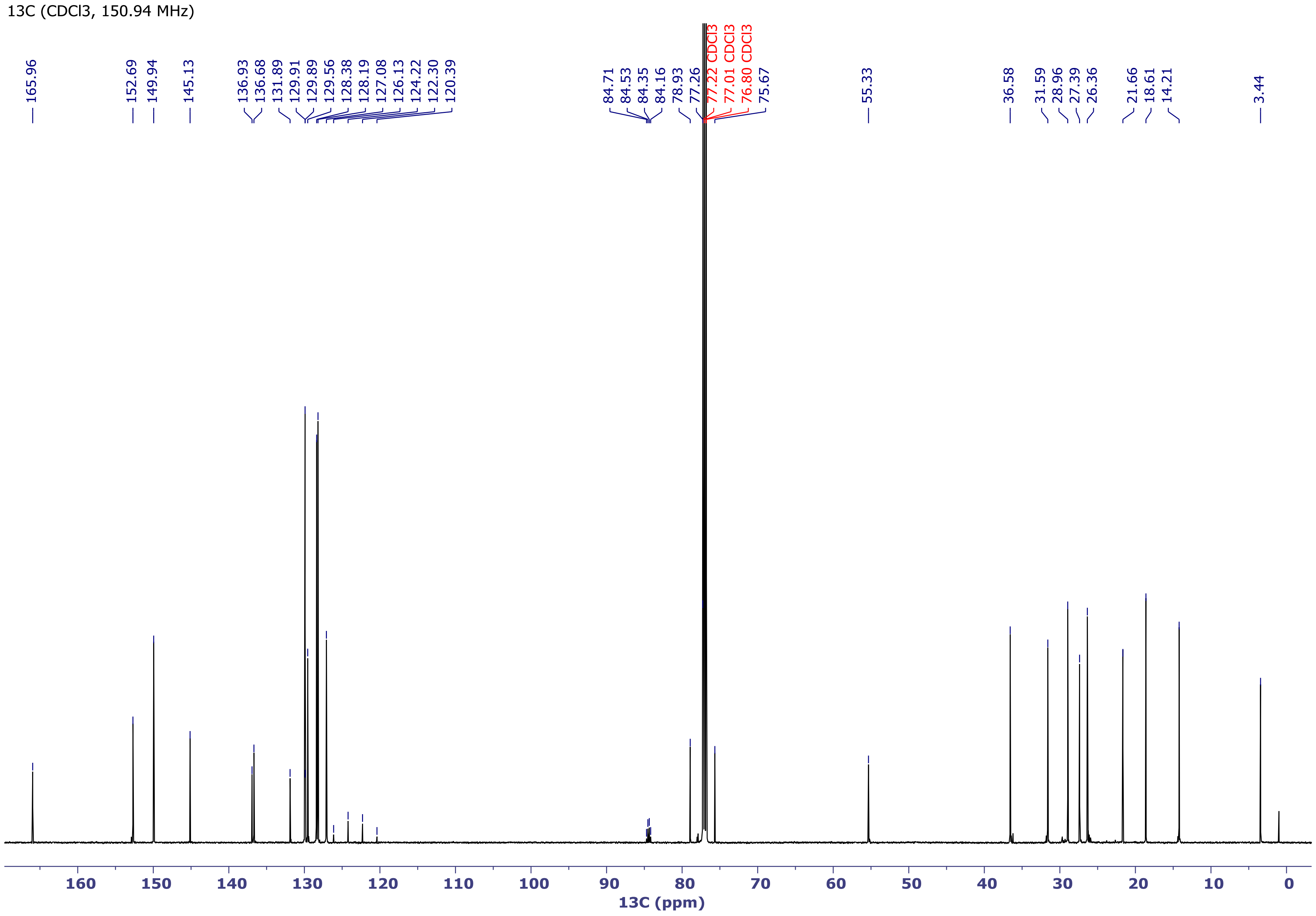
13C (CDCl3, 150.94 MHz)
165.96
152.69
149.94
145.13
136.93
136.68
131.89
129.91
129.89
129.56
128.38
128.19
127.08
126.13
124.22
122.30
120.39
84.71
84.53
84.35
84.16
78.93
77.26
77.22 CDCl3
77.01 CDCl3
76.80 CDCl3
75.67
55.33
36.58
31.59
28.96
27.39
26.36
21.66
18.61
14.21
3.44
160
150
140
130
120
110
100
90
80
70
60
50
40
30
20
10
0
13C (ppm)

Table S-2. Mosher ester analysis of (2*R*,3*R*)-3-methyl-1-(4-tosyloxazol-5-yl)dec-8-yn-2-ol **7b**, arbitrary numbering scheme as shown below.

Assignment	(*S*)-X [ppm]	(*R*)-X [ppm]	Δ(δ(S-R)) [ppm]
10a	3.48	3.47	0.01
10b	3.44	3.32	0.12
8	1.79	1.83	-0.04
7a	1.28	1.43	-0.15
7b	1.09	1.18	-0.09
6a	1.38	1.44	-0.06
6b	1.28	1.35	-0.07
11	1.00	1.04	-0.04

5-((2*R*,3*R*)-2-((*tert*-butyldimethylsilyl)oxy)-3-methyldec-8-yn-1-yl)-4-tosyloxazole (21b)

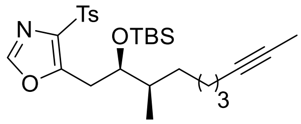

A Schleck tube equipped a magnetic stir bar was charged a solution of 2*R*,3*R*)-3-methyl-1-(4-tosyloxazol-5-yl)dec-8-yn-2-ol **7b** (130 mg, 33.3 µmol) in CH_2Cl_2 (2 mL) and 2,6-lutidine (77.7 µL, 66.8 µmol, 2.0 equiv.). The reaction mixture was cooled to 0 ◦C and *tert*-butyldimethylsilyl triflate (117 µL, 50.0 µmol, 1.5 equiv.) was added dropwise and the resultin mixture was allowed to warm to rt overnight. After that the solvent was removed under reduced pressure and the residue obtained was purified by flash column chromatography (SiO_2, 10 g, 20 mm ø, 0-25% EtOAc/iso-Hexanes, ca. 5 mL). This gave the product as a pale yellow oil (120 mg, 71 %).^{1}H NMR (400 MHz, $CDCl_3$): δ = 7.95 – 7.88 (m, 2H), 7.73 (s, 1H), 7.38 – 7.30 (m, 2H), 4.07 (m, 1H), 3.27 – 3.09 (m, 2H), 2.43 (s, 3H), 2.23 – 1.99 (m, 2H), 1.78 (t, *J* = 2.5 Hz, 3H), 1.49 – 1.25 (m, 6H), 1.19 – 1.03 (m, 1H), 0.97 (d, *J* = 6.8 Hz, 3H), 0.81 (s, 9H), -0.00 (s, 3H), -0.24 (s, 3H); ^{13}C NMR (101 MHz, $CDCl_3$): δ = 155.5, 149.5, 144.8, 137.4, 136.3, 129.9, 128.1, 79.2, 75.5, 74.5, 40.2, 31.8, 29.9, 28.7, 26.9, 25.7, 22.8, 19.3, 17.9, 15.7, 2.6, -4.7, -5.1; $[\alpha]_D^{20}$= + 41.4 (c = 1.08, $CHCl_3$); IR (film): $\tilde{\nu}$ = 3128, 2929, 2857, 1595, 1516, 1495, 1462, 1404, 1385, 1362, 1329, 1305, 1291, 1252, 1224, 1148, 1106, 1080, 1043, 1018, 1006, 920, 863, 837, 812, 776, 736, 706, 697, 661, 601, 540, 493 cm^{-1}; HRMS-ESI+ (*m/z*): calc'd. for $C_{27}H_{41}O_4NSSiNa$ $[M+Na]^+$, 526.2417; found, 526.2416.

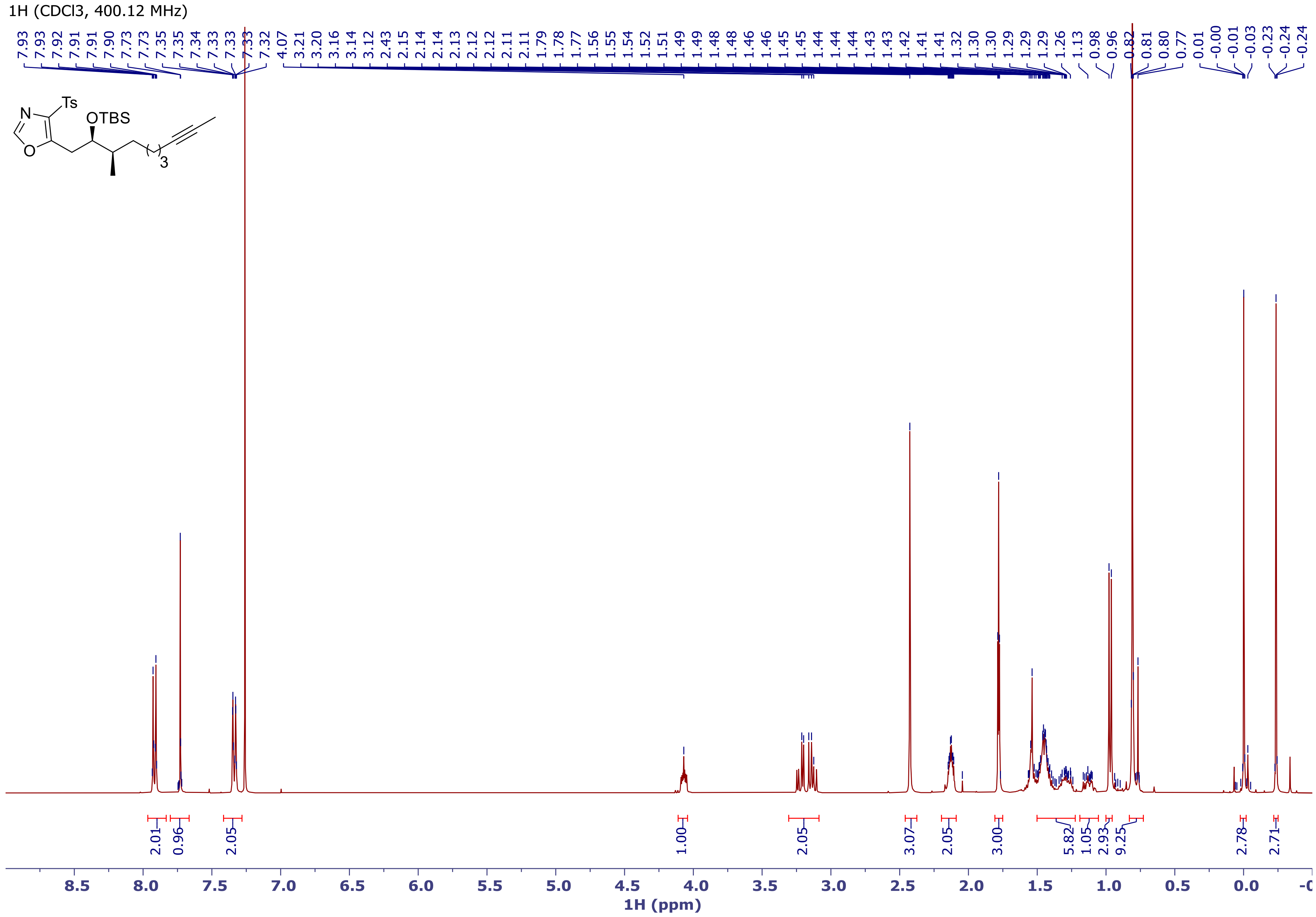
1H (CDCl3, 400.12 MHz)
Ts
N
O
OTBS
3
1H (ppm)

13C (CDCl3, 100.62 MHz)

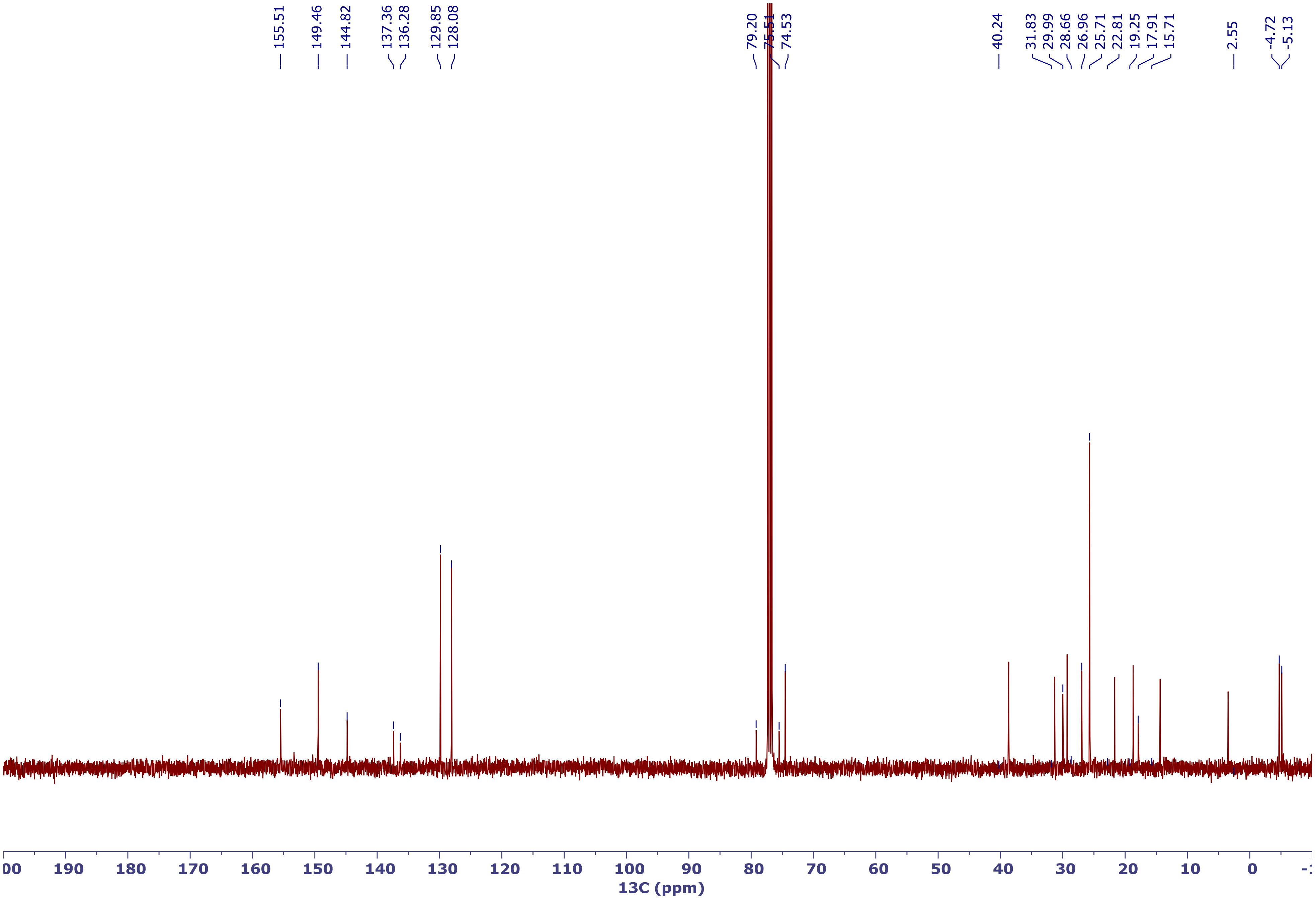

(2*R*,3*R*)-3-methyl-1-(oxazol-5-yl)oct-6-yn-2-yl (*E*)-2-methylhex-2-en-4-ynoate (4):

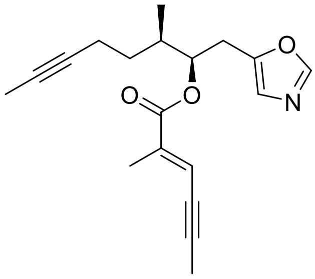

A Schlenk tube was charged with freshly prepared Na-Hg amalgam (5.0 g, 2.56 mmol Na, 10.0 equiv., 10 mol% Na), a solution of (2*R*,3*R*)-3-methyl-1-(4-tosyloxazol-5-yl)oct-6-yn-2-ol **7a** (250 mg, 256 µmol) in EtOH/THF (1:1, 5 mL) and Na_2HPO_4 (145 mg, 1.0 mmol, 4.0 equiv.). The resulting mixture was sonicated for 2 h. The reaction mixture was then diluted with EtOAc (20 mL), and then carefully decanted into water (10 mL). The aqueous phase was extract with EtOAc (2 x 10 mL), The combined organic extracts were dried over $MgSO_4$, filtered and then concentrated under reduced pressure. This gave the crude (2*R*,3*R*)-3-methyl-1-(oxazol-5-yl)oct-6-yn-2-ol **6a** as a cloudy syrup, which was used directly in the next step. A Schlenk tube equipped with a magnetic stir bar was charged with a solution of the crude (2*R*,3*R*)-3-methyl-1-(oxazol-5-yl)oct-6-yn-2-ol in CH_2Cl_2 (3 mL), DMAP (3.0 mg, 26.0 µmol, 10.0 mol%) and (*E*)-2-methylhex-2-en-4-ynoic acid **19** (13 mg, 102 µmol, 0.5 equiv.). The resulting mixture was cooled to 0°C. EDC·HCl (49 mg, 256 µmol, 1.0 equiv.) was added and the reaction mixture was allowed to warm to rt and stirred overnight. The solvent was then evaporated under reduced pressure and the residue obtained was purified by flash column chromatography (SiO_2, 10 g, 20 mm ø, 0-50% EtOAc/iso-Hexanes, ca. 5 mL). This gave the product as a pale yellow oil (24 mg, 73 %). ^{1}H NMR (400 MHz, $CDCl_3$): δ = 7.77 (s, 1H), 6.82 (d, *J* = 1.0 Hz, 1H), 6.57 (th, *J* = 2.6, 1.3 Hz, 1H), 5.17 (ddd, *J* = 7.6, 5.4, 4.0 Hz, 1H), 3.07 – 2.89 (m, 2H), 2.26 – 2.08 (m, 2H), 2.07 (d, *J* = 0.7 Hz, 3H), 2.00 (dd, *J* = 1.4, 0.7 Hz, 3H), 1.90 (dddt, *J* = 8.1, 6.8, 4.0, 1.4 Hz, 1H), 1.74 (t, *J* = 2.5 Hz, 3H), 1.66 – 1.53 (m, 1H), 1.41 – 1.27 (m, 1H), 0.98 (d, *J* = 6.8 Hz, 3H); ^{13}C NMR (101 MHz, $CDCl_3$) δ = 166.7, 150.7, 149.1, 137.8, 124.0, 120.9, 99.7, 78.4, 76.8, 76.3, 74.6, 35.0, 32.2, 27.9, 16.6, 15.2, 14.0, 5.0, 3.6; IR (film): $\tilde{\nu}$ = 2968, 2920, 2850, 2222, 2147, 1709, 1617, 1510, 1438, 1386, 1346, 1256, 1122, 1177, 969, 825, 742, 642, 485, 457, 432, 412 cm^{-1}; $[\alpha]_D^{20}$= + 26.5 (c = 0.78, $CHCl_3$); HRMS-CI (*m/z*): calc'd. for $C_{19}H_{24}NO_3$ $[M+H]^+$, 314.1751; found, 314.1752.

1H (CDCl3, 400.12 MHz)

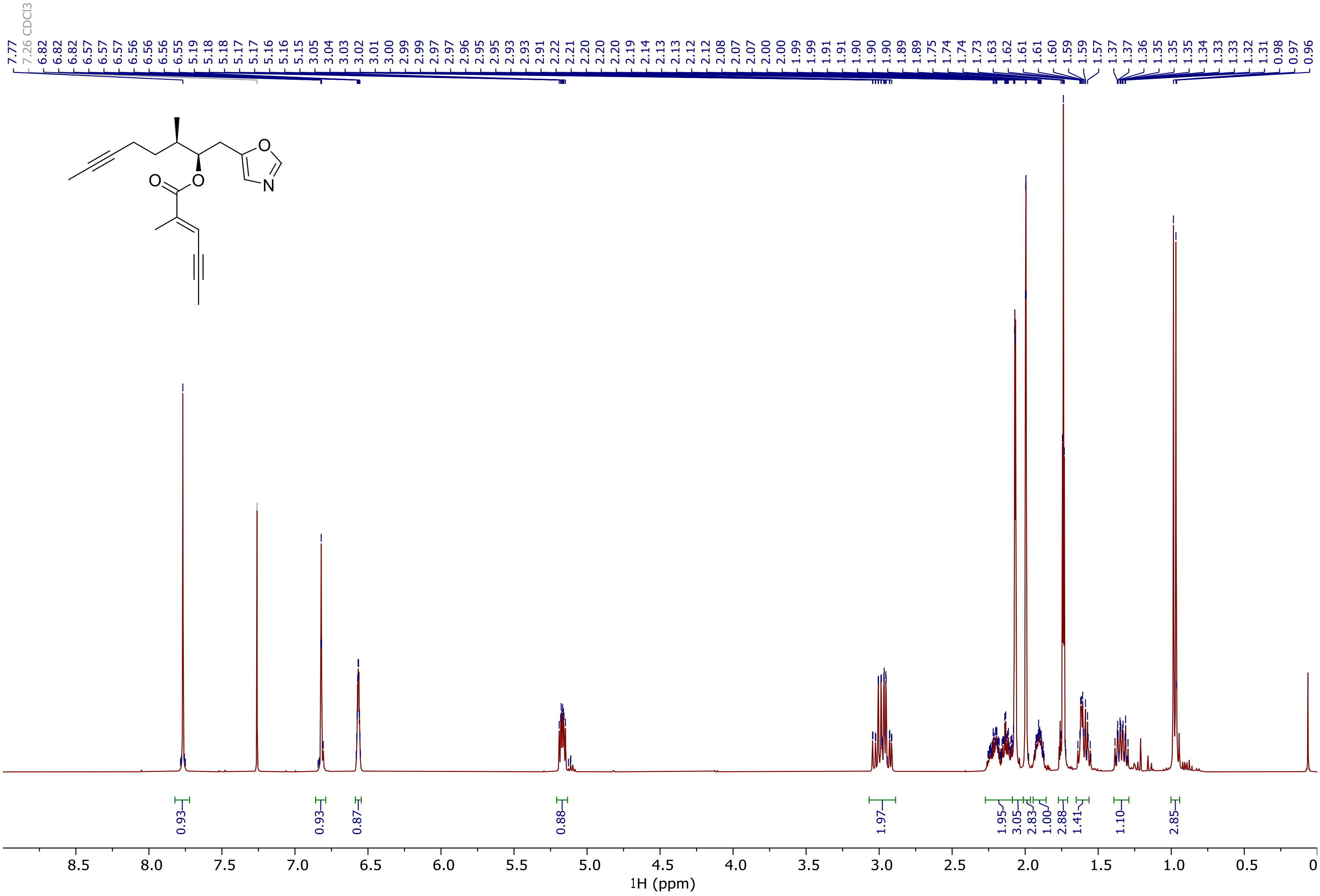

13C (CDCl3, 100.62 MHz)

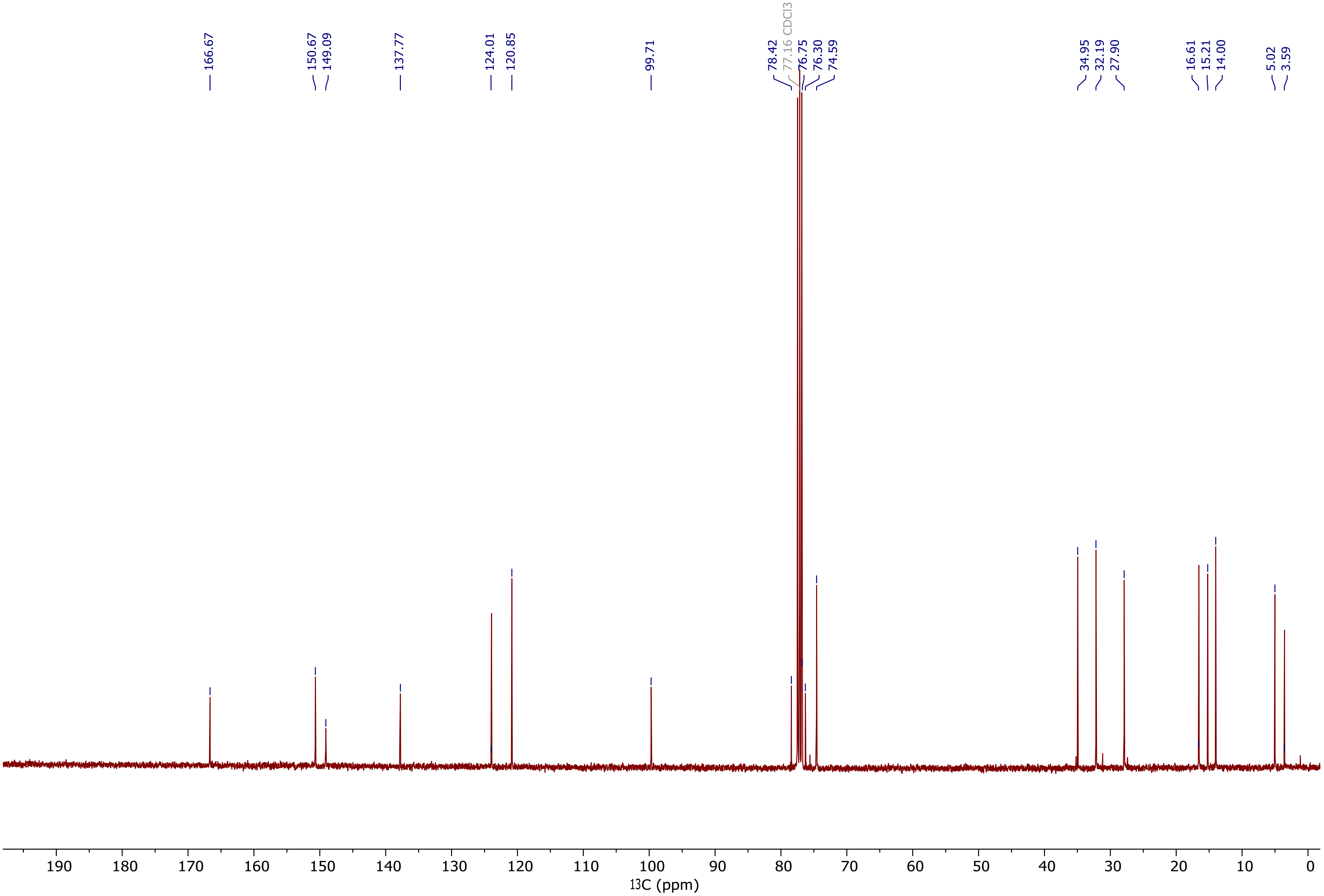

(3*E*,9*R*,10*R*,13*E*,19*R*,20*R*)-3,9,13,19-tetramethyl-10,20-bis(oxazol-5-ylmethyl)-1,11-dioxacycloicosa-3,13-dien-5,15-diyne-2,12-dione (2):

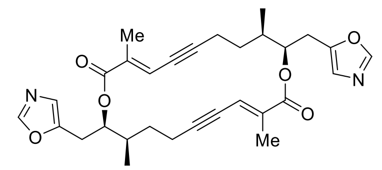

(2*R*,3*R*)-3-Methyl-1-(oxazol-5-yl)oct-6-yn-2-yl-(*E*)-2-methylhex-2-en-4-ynoate **4** (16 mg, 51.0 µmol) was added to a stirred suspension of complex **22a** (8 mg, 10.0 µmol, 20 mol%) and powdered molecular sieves 5Å (325 mg) in toluene (4 mL) at 23 °C under argon atmosphere. The mixture was heated at 60 °C stirred for 1 h, before it was filtered through a short pad of Celite® and rinsed with ethyl acetate (30 mL). The filtrate was evaporated and the residue was purified by by flash column chromatography (SiO_2, 10 g, 20 mm ø, 0-100% EtOAc/iso-Hexanes, ca. 5 mL) to give the product as a white solid (10 mg, 74 %). ^1H NMR (400 MHz, $CDCl_3$): δ = 7.76 (s, 2H), 6.81 (s, 2H), 6.48 (dh, *J* = 2.8, 1.3 Hz, 2H), 5.15 (ddd, *J* = 8.9, 4.8, 3.1 Hz, 2H), 2.98 – 2.83 (m, 4H), 2.63 (ddt, *J* = 17.1, 6.1, 2.8 Hz, 2H), 2.34 (ddt, *J* = 17.1, 11.6, 2.8 Hz, 2H), 2.25 – 2.12 (m, 2H), 1.99 – 1.74 (m, 8H), 1.47 – 1.35 (m, 2H), 1.03 (d, J = 7.1 Hz, 6H); ^{13}C NMR (101 MHz, $CDCl_3$) δ 166.4, 150.6, 149.5, 138.1, 123.7, 120.7, 103.0, 78.2, 75.7, 34.5, 29.8, 24.5, 18.4, 16.6, 15.2; IR (film): ṽ = 3127, 2958, 2923, 2852, 2357, 2209, 2171, 2145, 2056, 1706, 1614, 1511, 1459, 1435, 1385, 1364, 1342, 1257, 1170, 1123, 1097, 1008, 970, 918, 896, 825, 798, 787, 743, 661, 641, 536, 496, 480, 469, 450, 420, 407 cm^{-1}; $[\alpha]_D^{20}$= 11.5 (c = 0.2, $CHCl_3$); HRMS-ESI+ (*m/z*): calc'd. for $C_{30}H_{35}N_2O_6$ $[M+H]^+$, 519.2490; found, 519.2487.

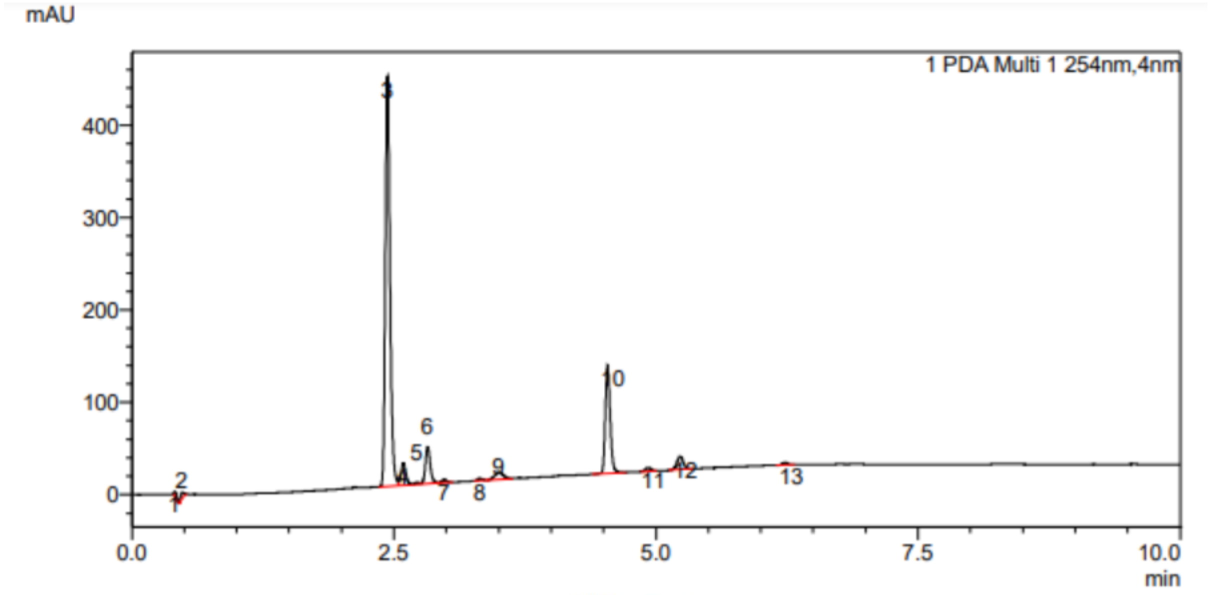

Acquired by : Ke
Sample Name : YIE-YA-367-01
Vial# : 11
Injection Volume : 1
Data File : YIE-YA-367-01-02.lcd
Method File : Yiannakas.lcm

1 µL YIE-YA-367-01 (GS in 1m L Acetonitril, filtriert)
20 µL in 180 µL Acetonitril
50 mm Eclipse Plus C18 1.8 µm, 3.0 mm i.D.,
Methanol / Wasser - Gradient:
70% - 5' - 90% MeOH
0.5 mL / min, 21.5 MPa, 308 K

PDA Ch1 254nm

Peak#	Ret. Time	Area%	
1	0.41	0.53	
2	0.48	0.71	
3	2.44	64.29	→ **2**
4	2.59	3.62	
5	2.72	0.32	
6	2.82	6.06	→ **4**
7	2.98	0.50	
8	3.32	0.36	
9	3.50	2.21	
10	4.54	17.61	→ **L1**
11	4.93	0.59	
12	5.23	2.79	
13	6.24	0.41	
Total		100.00	

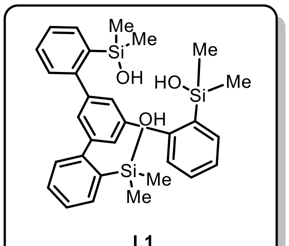

L1

Figure S1. Annotated UHPLC trace of a crude aliquot of one-pot ACM-RCAM reaction of bis(alkyne) **4** after 50 mins at 60 ◦C, 20 mol% **22a**, Reaction Molarity (mM): 30 (Reaction monitored @ 254 nm).

1H (CDCl3, 400.12 MHz)

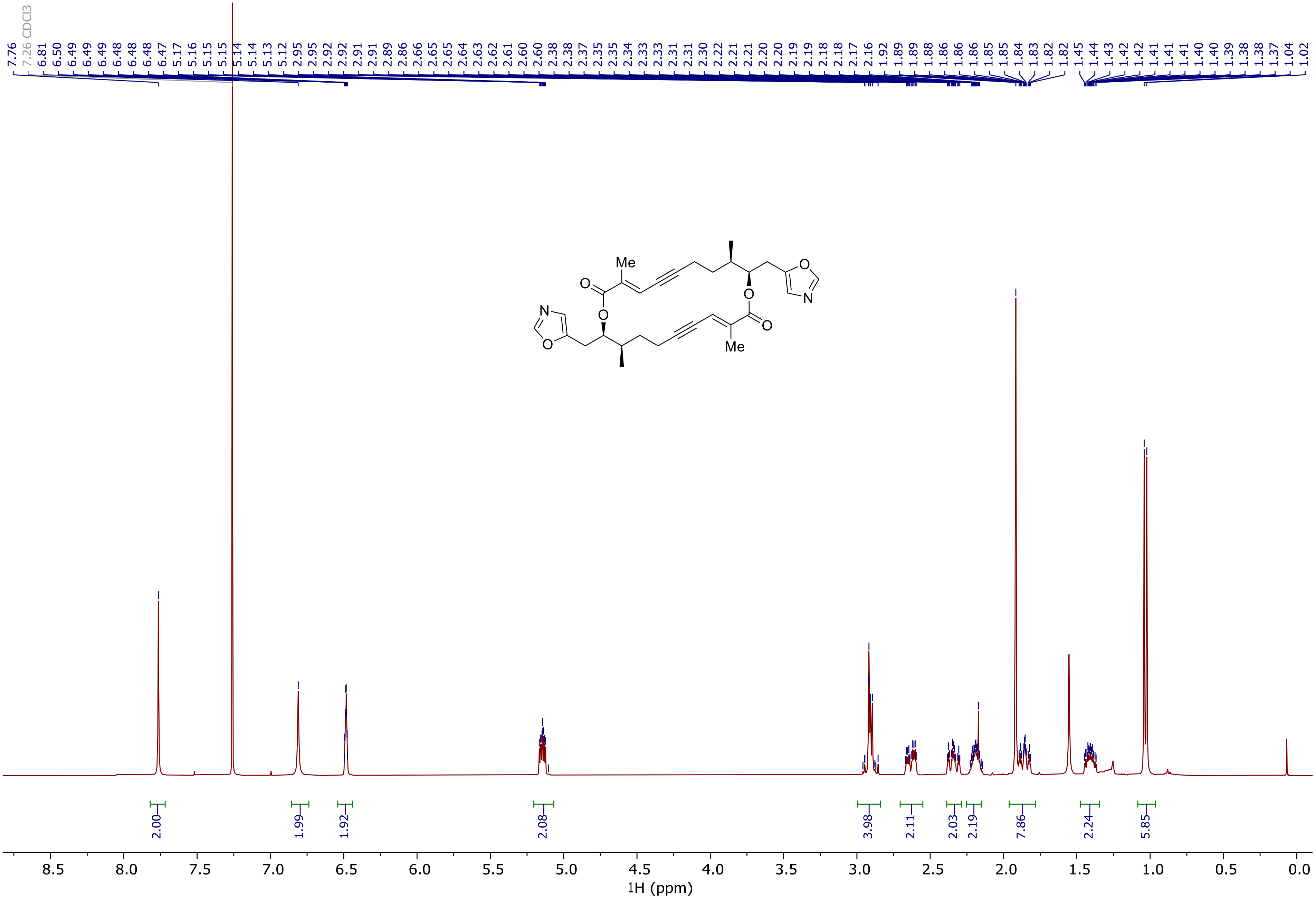

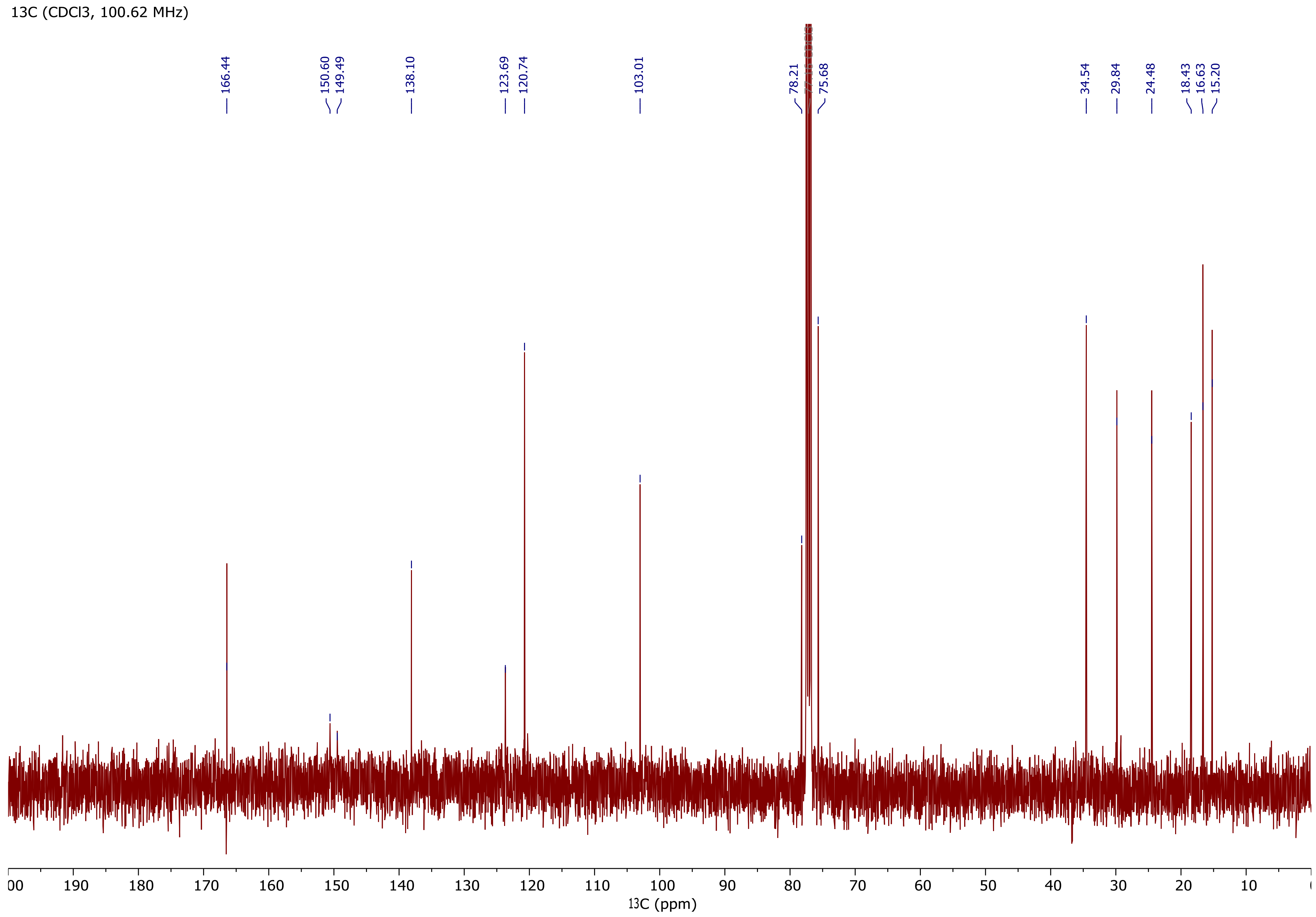
13C (CDCl3, 100.62 MHz)
166.44
150.60
149.49
138.10
123.69
120.74
103.01
78.21
75.68
34.54
29.84
24.48
18.43
16.63
15.20
190
180
170
160
150
140
130
120
110
100
90
80
70
60
50
40
30
20
10
13C (ppm)

Samroiyotmycin A (1)

A solution of macrocycle **2** (6.5 mg, 12.5 µmol) in CH_2Cl_2 (1.0 mL) was added to $[RuCp^*Cl]_4$ (**23**) (1.4 mg, 1.2 µmol, 10 mol%) with vigorous stirring. A solution of Bu_3SnH (8.4 µL, 25.0 µmol, 2.0 equiv.) in CH_2Cl_2 (0.1 mL) was added dropwise and the reaction mixture was stirred at rt for 0.5 h. After that, the solvent was removed under reduced pressure and the residue obtained was dissolved in DMF/MeOH (10:1, 500 µL). $CuPO_2Ph_2$ (**24**) (19.5 µg, 68.4 µmol, 2.0 equiv.) was added and the resulting mixture was stirred for 0.5 h at rt. The solvent was removed under reduced pressure and the residue was purified by preparative HPLC (50 mm Eclipse Plus C18 1.8 µm, 3.0 mm i.d. Methanol / Water-Gradient:70% - 5' - 95% MeOH, 0.5 mL / min, 18.0 MPa, 308 K, UV, 254 nm). This afforded Samroiyotmycin A (**1**) as a white solid (2 mg, 31 %). ^{1}H NMR (600 MHz, $CDCl_3$): δ = 7.76 (s, 1H), 6.94 (dq, *J* = 11.3, 1.3 Hz, 1H), 6.80 (s, 1H), 6.27 (dddd, *J* = 15.2, 11.3, 1.8, 0.8 Hz, 1H), 5.97 (ddd, *J* = 14.9, 9.0, 5.8 Hz, 1H), 5.09 (ddd, *J* = 9.1, 4.2, 3.0 Hz, 1H), 2.92 (ddd, *J* = 15.6, 4.2, 1.0 Hz, 1H), 2.87 (ddd, *J* = 15.5, 9.1, 0.9 Hz, 1H), 2.46 – 2.39 (m, 1H), 2.08 (tdd, *J* = 14.2, 9.2, 1.8 Hz, 1H), 1.86 – 1.74 (m, 5H), 1.44 – 1.38 (m, 1H), 0.99 (d, *J* = 7.1 Hz, 3H); ^{13}C NMR (151 MHz, $CDCl_3$): δ = 167.7, 150.5, 149.6, 142.5, 138.3, 127.6, 125.7, 123.7, 75.3, 34.4, 30.0, 29.9, 25.0, 16.5, 12.8; IR (film): ṽ = 2958, 2922, 2853, 1700, 1634, 1607, 1510, 1460, 1388, 1364, 1312, 1288, 1239, 1102, 1193, 1156, 1102, 1012, 972, 922, 825, 746, 648 cm^{-1}; $[\alpha]_D^{20}$= + 76.7 (c = 0.08, MeOH); HRMS-ESI+ (*m/z*): calc'd. for $C_{30}H_{38}N_2O_6$ $[M+Na]^+$, 545.2622; found, 545.2620.

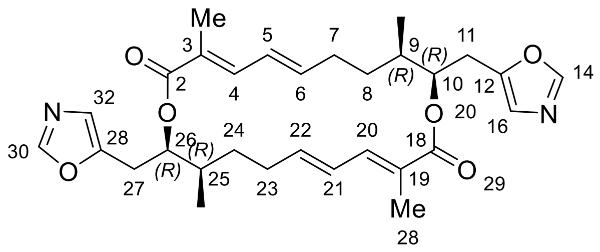

Table S-4. Assignment of ^{1}H & ^{13}C NMR spectra of synthetic *Samroiyotmycin A*.

$[\alpha]_D^{20}$	+76.7°, c = 0.08		
Atom	^{1}H NMR δ (ppm, *J* [Hz])	Atom	^{13}C NMR δ [ppm]
2/18	-	2/18	167.7
3/19	-	3/19	125.7
4/20	6.94 (dq, 11.3, 1.3)	4/20	138.3
5/21	6.27 (dddd, 15.2, 11.3, 1.8, 0.8)	5/21	127.6
6/22	5.97 (ddd, 14.9, 9.0, 5.8)	6/22	142.5
7/23	2.08 (tdd, 14.2, 9.2, 1.8) and 2.46 – 2.39 (m)	7/23	29.9
8/24	1.38 - 1.44 (m) and 2.38 -2.46 (m)	8/24	30.0
9/25	1.73 – 1.85 (m)	9/25	34.4
10/26	5.09 (ddd, 9.10, 4.2, 3.0)	10/26	75.3
11/27	2.87 (ddd, 15.5, 9.1, 1.0) and 2.92 (ddd, 15.6, 4.2, 1.0)	11/27	25.0
12/28	-	12/28	149.6

14/30	7.76 (s)	14/30	150.5
16/32	6.80 (s)	16/32	123.7
3-CH_3	1.82 (s)	3-CH_3	12.8
9-CH_3	0.99 (d, 7.1)	9-CH_3	16.5
25-CH_3	0.99 (d, 7.1)	25-CH_3	16.5
19-CH_3	1.82 (s)	19-CH_3	12.8

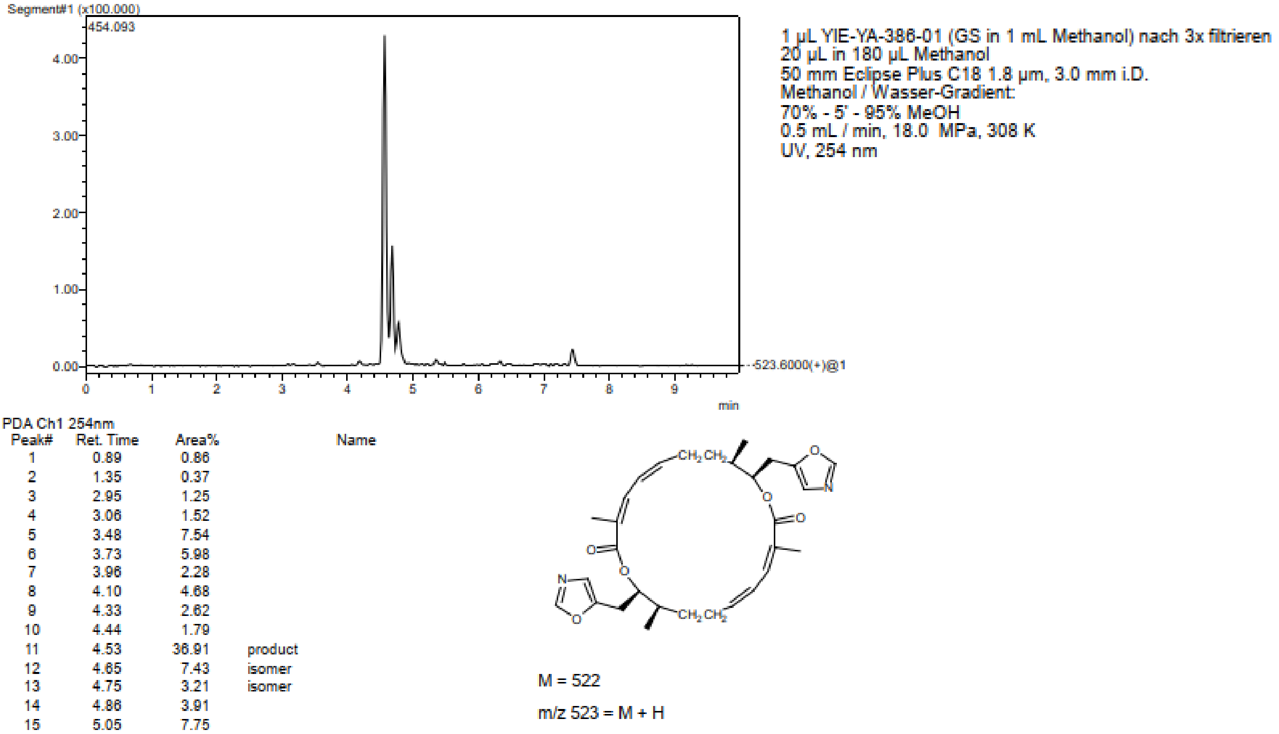

PDA Ch1 254nm

Peak#	Ret. Time	Area%	Name
1	0.89	0.86	
2	1.35	0.37	
3	2.95	1.25	
4	3.06	1.52	
5	3.48	7.54	
6	3.73	5.98	
7	3.96	2.28	
8	4.10	4.68	
9	4.33	2.62	
10	4.44	1.79	
11	4.53	36.91	product
12	4.65	7.43	isomer
13	4.75	3.21	isomer
14	4.86	3.91	
15	5.05	7.75	
16	5.18	1.53	
17	5.32	1.59	
18	5.49	2.44	
19	5.65	1.16	
20	5.85	0.93	
21	6.52	2.33	
22	7.88	1.92	
Total		100.00	

Figure S-3. Evidence for the selectivity of observed during the bis (trans-reduction) of macrocycle **2** during the bis(*trans*-hydrostannylation) – bis (protodestannylation) sequence (78:22; ratio of product **1** to other isomers).

Table S-5. Comparison of NMR data between the isolate and synthetic *Samroiyotmycin A*.

	ISOLATED	SYNTHETIC		ISOLATED	SYNTHETIC
$[\alpha]_D$	+80.9°, c = 0.09	+76.7°, c = 0.08			
Atom	^{1}H NMR δ (ppm, *J* [Hz])		Atom	^{13}C NMR δ [ppm]	
2/18	-	-	2/18	167.7	167.7
3/19	-	-	3/19	125.8	125.7
4/20	6.94 (d, 11.2)	6.94 (dq, 11.3, 1.3)	4/20	138.4	138.3
5/21	6.27 (dd, 14.3, 11.2)	6.27 (dddd, 15.2, 11.3, 1.8, 0.8)	5/21	127.7	127.6
6/22	5.93 – 6.00 (m)	5.97 (ddd, 14.9, 9.0, 5.8)	6/22	142.6	142.5
7/23*	1.76 (d, 12.9) and 2.08 (dd, 12.9, 11.3)	2.08 (tdd, 14.2, 9.2, 1.8) and 2.46 – 2.39 (m)	7/23	29.9	29.9
8/24*	1.36- 1.45 (m) and 2.38 - 2.48 (m)	1.38 - 1.44 (m) and 2.38 -2.46 (m)	8/24	30.1	30.0
9/25	1.78 – 1.88 (m)	1.73 – 1.85 (m)	9/25	34.4	34.4
10/26	5.09 (quin, 3.8)	5.09 (ddd, 9.10, 4.2, 3.0)	10/26	75.3	75.3
11/27	2.83 – 2.95 (m)	2.87 (ddd, 15.5, 9.1, 1.0) and 2.92 (ddd, 15.6, 4.2, 1.0)	11/27	25.1	25.0
12/28	-	-	12/28	149.7	149.6
14/30	7.78 (s)	7.76 (s)	14/30	150.6	150.5
16/32	6.80 (s)	6.80 (s)	16/32	123.8	123.7
3-CH_3	1.88 (s)	1.82 (s)	3-CH_3	12.9	12.8
9-CH_3	0.98 (d, 7.1)	0.99 (d, 7.1)	9-CH_3	16.5	16.5
25-CH_3	0.98 (d, 7.0)	0.99 (d, 7.1)	25-CH_3	16.5	16.5
19-CH_3	1.88 (s)	1.82 (s)	19-CH_3	12.9	12.8

*Peaks were mis-assigned in the original isolation paper.[7]

1H (CDCl3, 600.20 MHz)

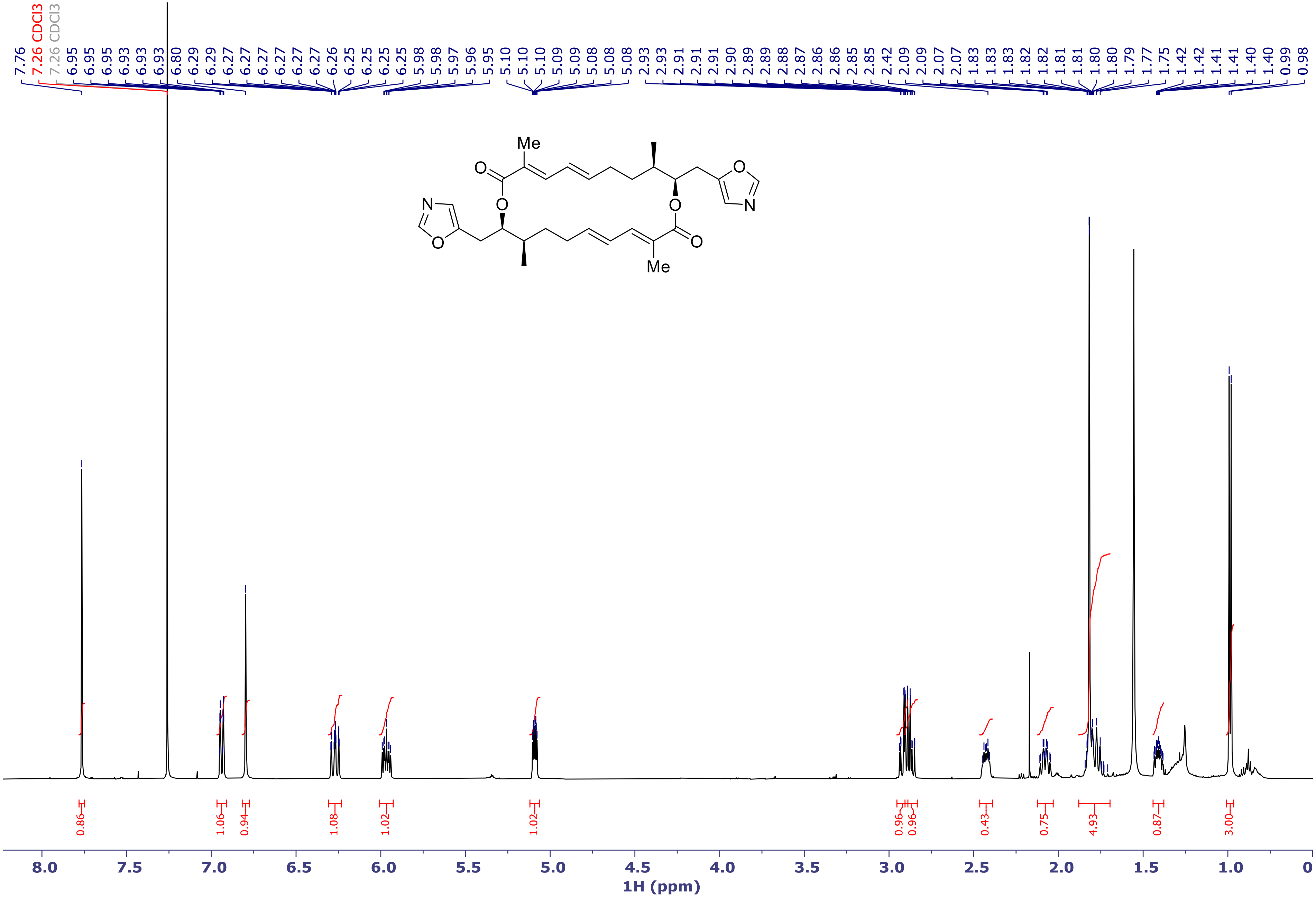

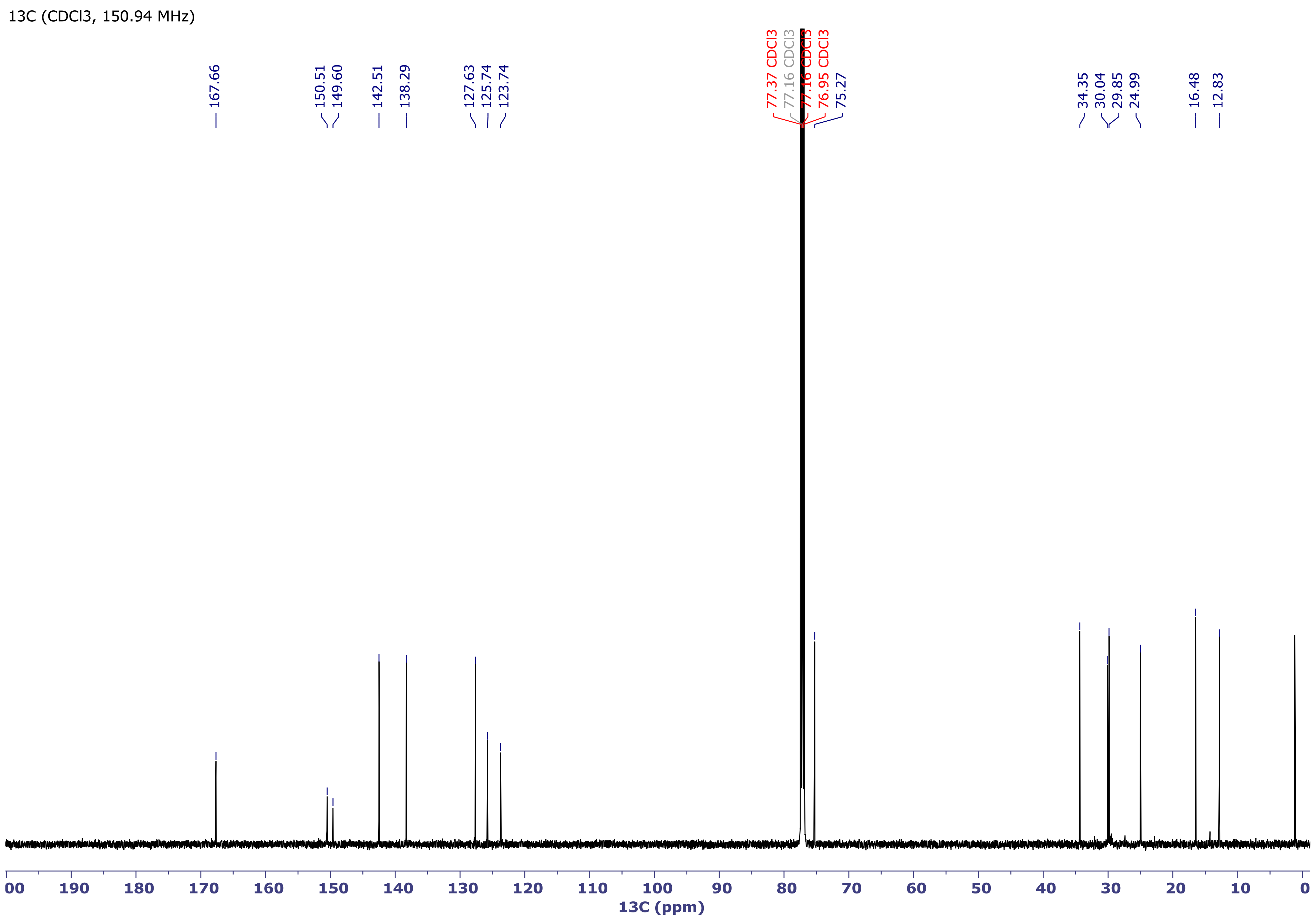
13C (CDCl3, 150.94 MHz)
167.66
150.51
149.60
142.51
138.29
127.63
125.74
123.74
77.37 CDCl3
77.16 CDCl3
77.16 CDCl3
76.95 CDCl3
75.27
34.35
30.04
29.85
24.99
16.48
12.83
00
190
180
170
160
150
140
130
120
110
100
90
80
70
60
50
40
30
20
10
0
13C (ppm)

YIE-YA-386-40 — 1H COSY @ 298K — 1 mg CDCl3 av600neo cryoBBO — 28/10/2020

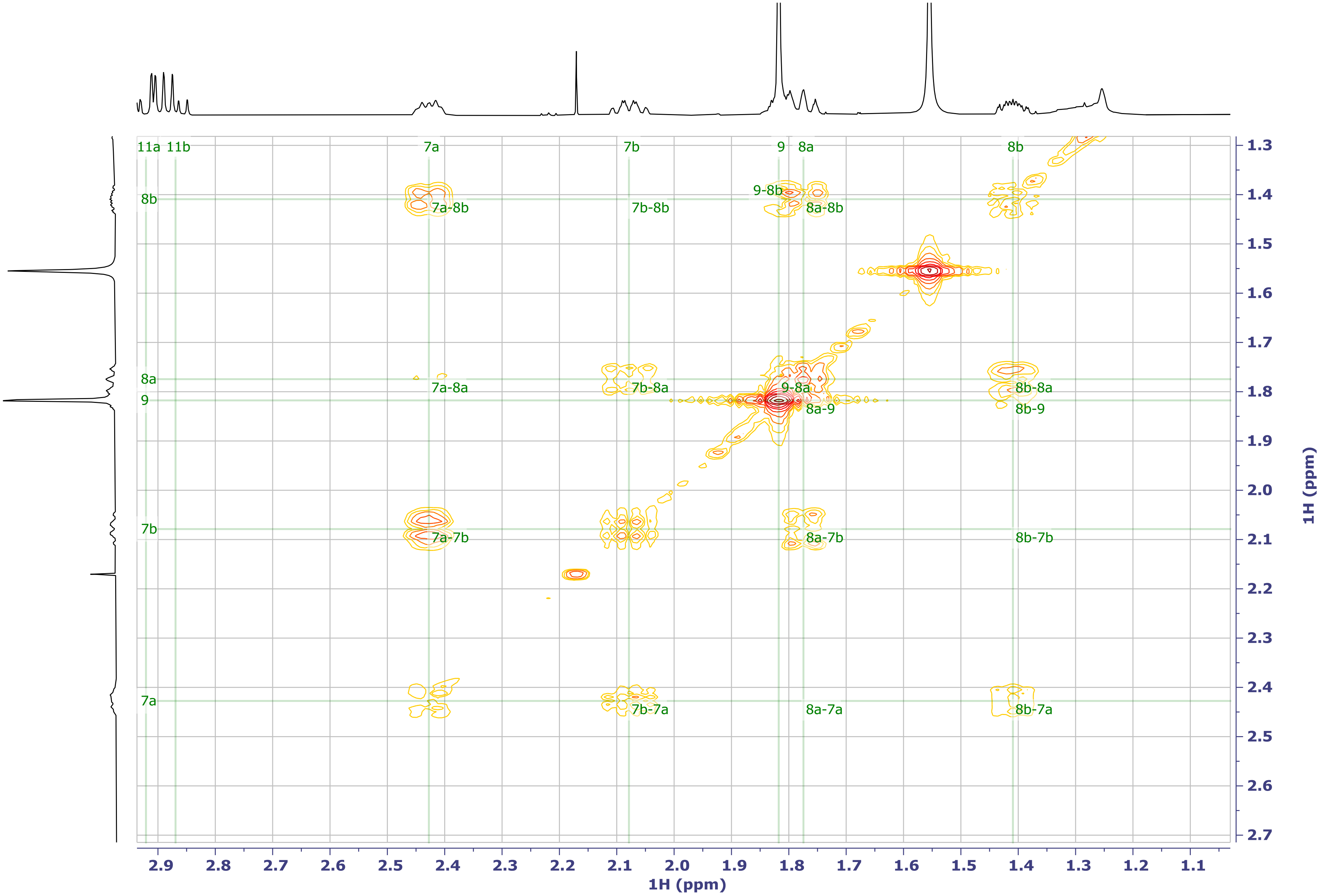

yieya38640.13.1.2rr — YIE-YA-386-40 — 1H COSYPH @ 298K — 1 mg CDCl3 av600neo cryoBBO — 28/10/2020

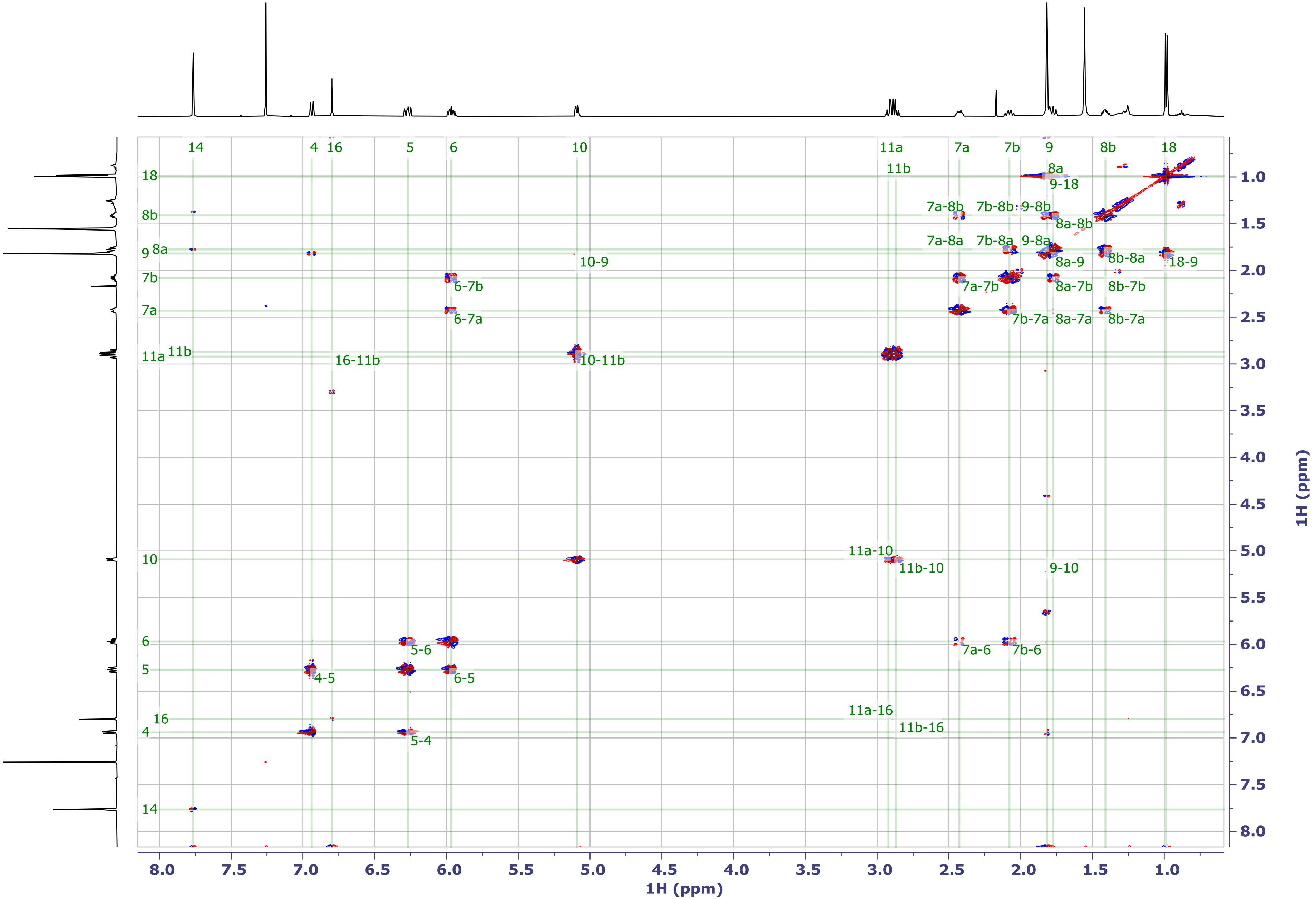

yieya38640.14.1.2rr — YIE-YA-386-40 — 13C-HSQC @ 298K — 1 mg CDCl3 av600neo cryoBBO — 28/10/2020

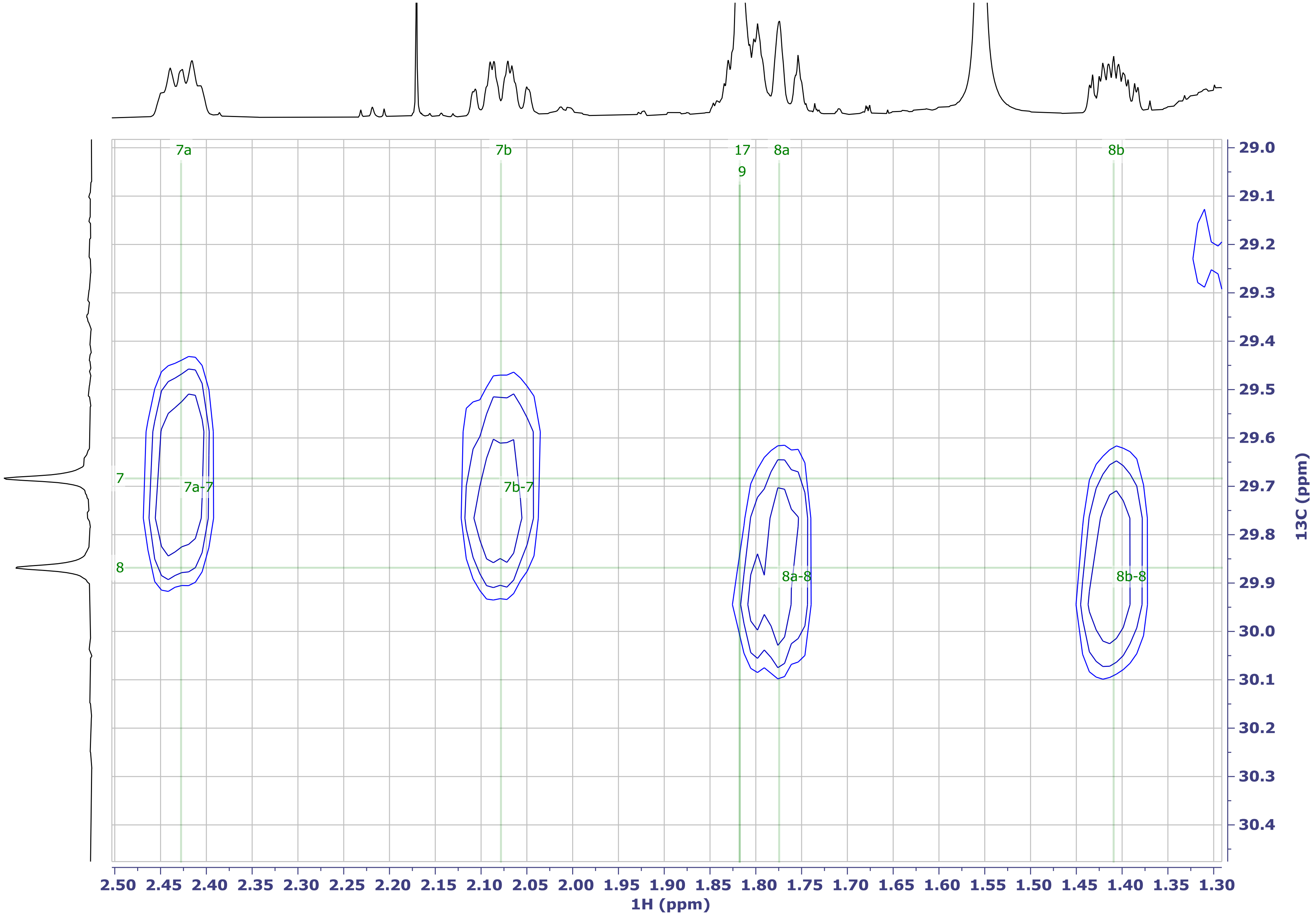

yieya38640.15.1.2rr — YIE-YA-386-40 — 13C-HMBC @ 298K — 1 mg CDCl3 av600neo cryoBBO — 28/10/2020

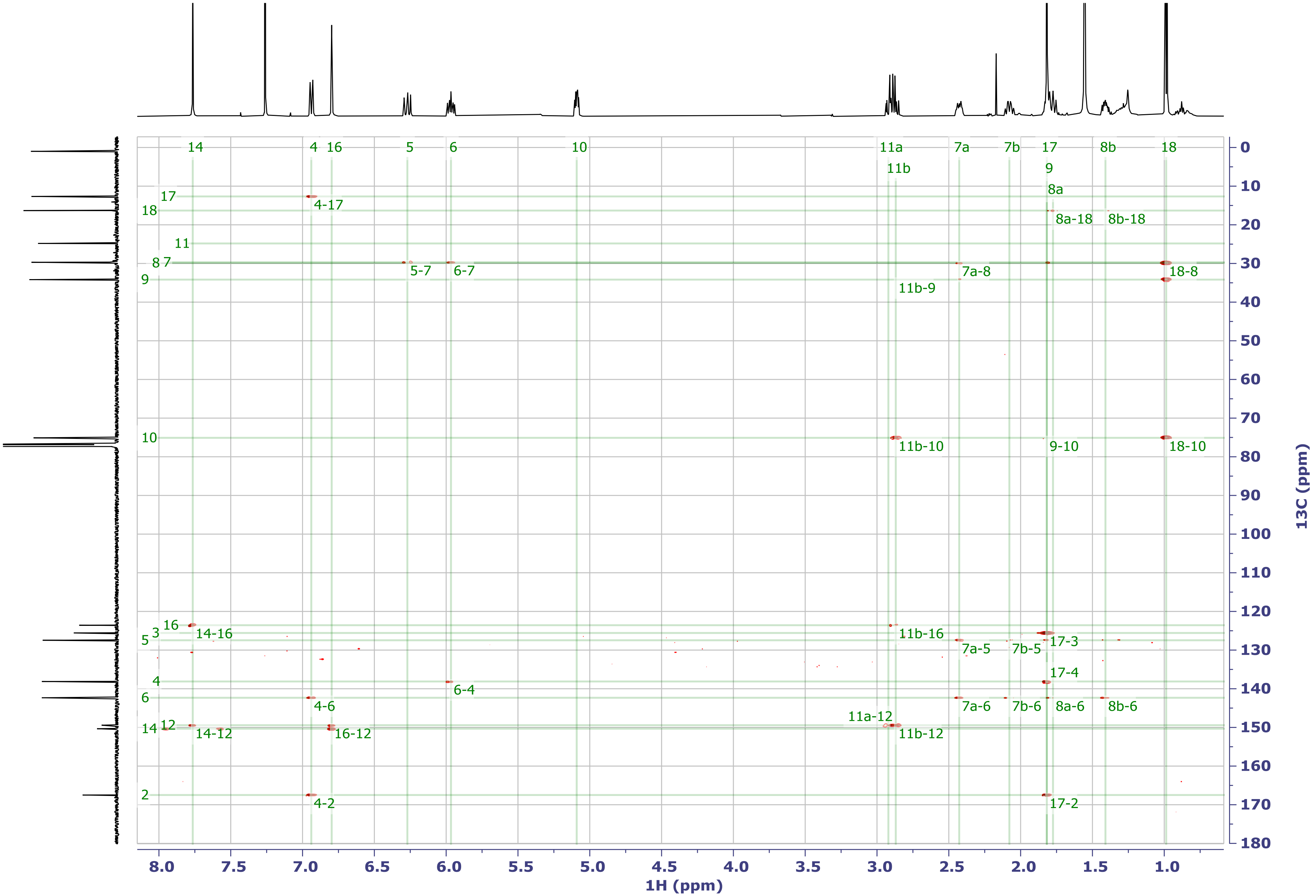

yieya38640.16.1.2rr — YIE-YA-386-40 — 1H NOESY @ 298K — 1 mg CDCl3 av600neo cryoBBO — 28/10/2020

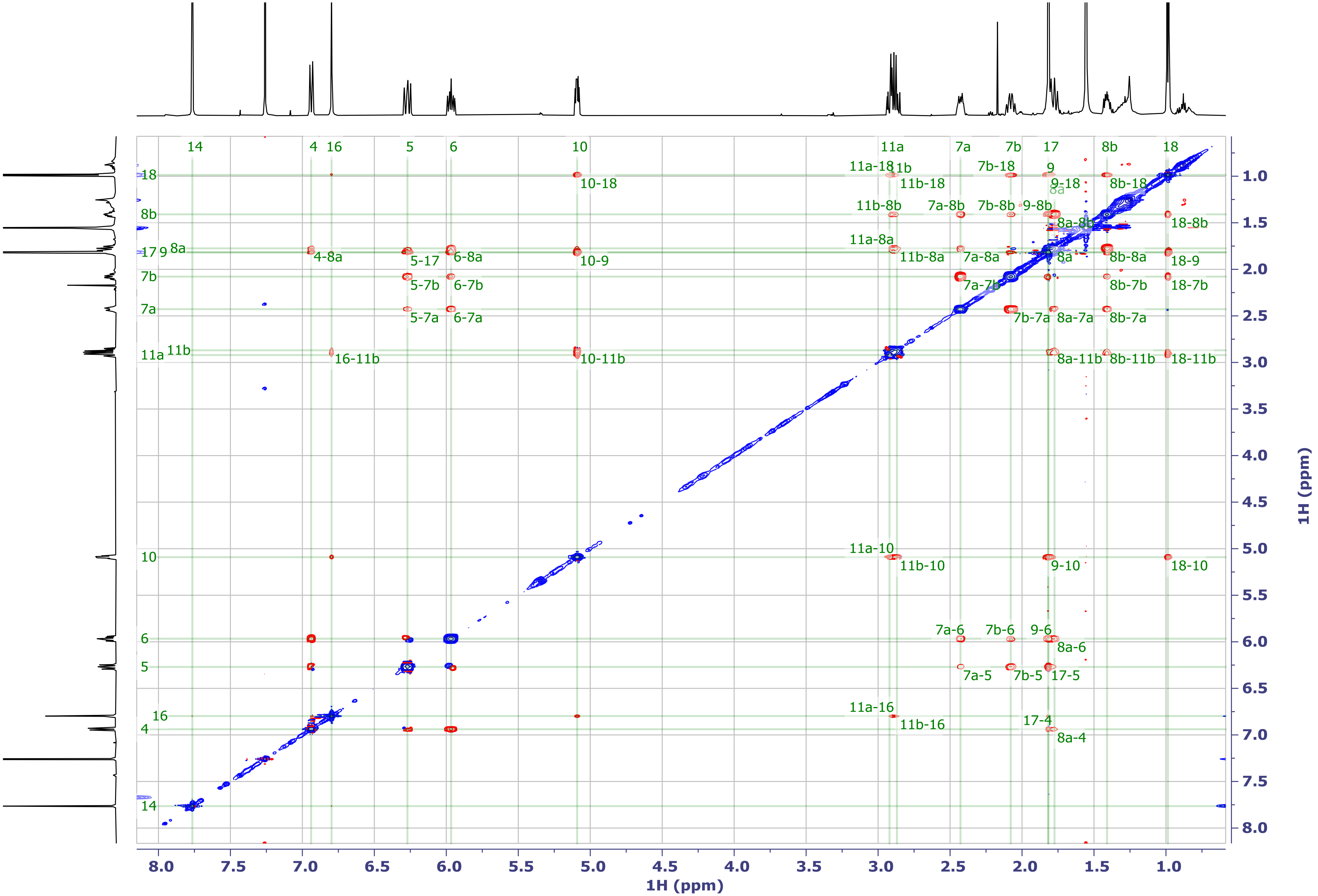

References

[1] W. Fang and B. Breit, *Angew. Chem. Int. Ed.*, **2018**, 57, 14817-14821.
[2] Z. Meng, A. Fürstner, *J. Am. Chem. Soc.* **2020**, 142, 11703–11708.
[3] J. Robinson, H. Flohr, U. Kempe, W. Panhorst, J. Rétey, *Liebigs Ann. Chem.*, **1983**, 2, 181-203.
[4] C. Zhu, X. Shen, S. Nelson, *J. Am. Chem. Soc.,* **2004**, 126, 5352-5353.
[5] D. Mailhol, J. Willwacher, N. Kausch-Busies, E. Rubitski, Z. Sobol, M. Schuler, M. Lam, S. Musto, F. Loganzo, A. Maderna, A. Fürstner, *J. Am. Chem. Soc.*, **2014**, 136, 15719-15729.
[6] S. Saito, S. Nakagawa, T. Koizumi, K. Hirayama and Y. Yamamoto, *J. Org. Chem.*, **1999**, 64, 3975-3978.
[7] A. Dramae, S. Nithithanasilp, W. Choowong, P. Rachtawee, S. Prabpai, P. Kongsaeree and P. Pittayakhajonwut, *Tetrahedron*, **2013**, 69, 8205-8208.
